# The impact of wind energy on plant biomass production in China

**DOI:** 10.1038/s41598-023-49650-9

**Published:** 2023-12-15

**Authors:** Li Gao, Qingyang Wu, Jixiang Qiu, Yingdan Mei, Yiran Yao, Lina Meng, Pengfei Liu

**Affiliations:** 1 School of Economics and Management, China University of Petroleum Beijing, Beijing, 102249 People’s Republic of China; 2 Fielding School of Public Health, University of California, Los Angeles, Los Angeles, CA 90095 USA; 3 School of Applied Economics, Renmin University of China, Beijing, 100872 People’s Republic of China; 4 School of Economics and The Wang Yanan Institute for Studies in Economics, Xiamen University, Xiamen, 361005 Fujian People’s Republic of China; 5 Department of Environmental and Natural Resources Economics, University of Rhode Island, Kingston, RI 02881 USA

**Keywords:** Environmental economics, Environmental economics

## Abstract

Global wind power expansion raises concerns about its potential impact on plant biomass production (PBP). Using a high-dimensional fixed effects model, this study reveals significant PBP reduction due to wind farm construction based on 2404 wind farms, 108,361 wind turbines, and 7,904,352 PBP observations during 2000–2022 in China. Within a 1–10 km buffer, the normalized differential vegetation and enhanced vegetation indices decrease from 0.0097 to 0.0045 and 0.0075 to 0.0028, respectively. Similarly, absorbed photosynthetically active radiation and gross primary productivity decline from 0.0094 to 0.0034% and 0.0003–0.0002 g*C/m^2^ within a 1–7 km buffer. Adverse effects last over three years, magnified in summer and autumn, and are more pronounced at lower altitudes and in plains. Forest carbon sinks decrease by 12,034 tons within a 0–20 km radius, causing an average economic loss of $1.81 million per wind farm. Our findings underscore the balanced mitigation strategies for renewable energy transition when transiting from fossil fuels.

## Introduction

Environmental degradations and extreme weather driven by climate change are significant threats to biodiversity and ecosystems^1^. Fossil fuel consumption has been a major contributor to this problem, leading to various forms of pollution and greenhouse gas emissions. The Paris Agreement encourages legally binding international treaties on climate change and sustainable development by enhancing the implementation of nationally determined contributions. The Agreement also emphasizes the critical role of renewable energy in these efforts^2^.

Traditional fossil fuel-based power generation, especially coal-fired power plants, has significantly affected the health of vegetation. The emissions of sulfur oxides and nitrogen oxides from plants not only lead to acid rain but also lower the pH value of the atmosphere, subsequently damaging soil structure and biological activity^3^. Such acidic conditions inhibit the uptake of vital nutrients in many plants, including calcium, magnesium, and potassium, hindering their growth^4^. Moreover, heavy metals like mercury, cadmium, and lead, released from coal-burning, accumulate in soils, could enter the food chain and pose threats to higher-order organisms^5^. In addition to these direct impacts, emissions of particulates and other gases may alter local microclimates, affecting temperature and humidity^6^. The particulate matter from coal combustion can also impede sunlight penetration, resulting in reduced light intensity or shifts in light quality, which, in turn, affects photosynthesis and other physiological processes in certain plants^7^. Owing to these multifaceted factors, regions under long-term influence of coal-fired power plants might experience a decline in biodiversity. Such pollution could render certain plant species less viable, leading to shifts in ecosystem structure and functionality.

Wind energy is one of the fastest-growing energy sources, playing a vital role in global efforts to mitigate climate change. Wind energy is often favored over photovoltaic solar energy for its superior grid-balancing capabilities^8,9^. However, wind farms might threaten carbon sink and plant biomass production (PBP), a vital component of carbon storage and uptake in ecosystems^10–12^. Wind farm construction may also affect biodiversity by reshaping of trophic cascades^13,14^, introducing new species into given ecological systems^15,16^, altering species migration patterns^17^, killing birds through collision with turbines^18^, habitat degradation or provision^18–22^, chemical element deposition^23^, changing local microclimates^24–26^, and others. Even though these negative impacts may not deter the shift from fossil fuel reliance to cleaner energy sources, policymakers and institutions often prioritize renewable energy transition without considering potential ecological impacts such as PBP conservation, leading to unsustainable resource use and increasing biodiversity destruction.

Plants play a critical role in ecosystem functioning and global biodiversity, and are highly vulnerable to damages caused by climate change^1,28^. Climate change, largely accelerated by fossil fuel use, is making them increasingly vulnerable. Wind farms also have mixed effects on ecosystems, they can alter local surface temperature, humidity, and precipitation^19,22,29^. They may extend regional vegetation growing seasons and increase grassland productivity^27,30^, and turbine bases may provide favorable habitats for some terrestrial species^31^. Previous studies found the spatial impact of wind farms on vegetation ecosystem may extend for 10 km beyond the base of the wind turbines and the tower^32^. Plants in developing countries are particularly vulnerable^33,34^. Existing literature focuses on the effects of wind farm construction on animal diversity, and the potential impacts on vegetation dynamics are understudied. Therefore, a nuanced understanding of the ecological implications of both renewable and non-renewable energy is vital.

In the context of the pressing need to transition away from fossil fuels, China has the largest installed wind energy capacity in the world, following rapid growth in new wind facilities since the first wind farm in 1986^35,36^. As of the end of 2020, the country had, cumulatively, 281 gigawatt (GW) installed wind power capacity, having added 71.6 GW of new capacity that same year; in contrast the United States had installed capacity of 118 GW, with 14 GW of new capacity added in 2020^37^. China also operates almost half of the world’s installed offshore wind power, amounting to 170 GW as of 2021^8,38,39^. This reliance on wind power is likely to grow, as the government announced ambitious carbon emissions goals at the 75th session of the United Nations General Assembly in 2020, pledging to peak before 2030 and achieve carbon neutrality before 2060 (UNGA 75). Leaders further announced the goal of a non-fossil energy share of 25% in primary energy consumption by 2030 and 80% by 2060. China’s 14th 5-Year Plan (2021–2025) aims to reduce the number of wind farm units and investment costs by constructing wind farms with larger capacity turbines, swept areas, and higher hub heights and rotor diameters to maintain low wind power prices. China is one of the wealthiest countries in PBP globally and a signatory to the UN Convention on Biological Diversity^40^. While the emphasis on wind power is in line with global sustainability goals, it’s crucial that these efforts are executed with care for local ecosystems. Balancing renewable energy production with biodiversity conservation remains a challenge and necessary issue to address.

**Figure 1:**
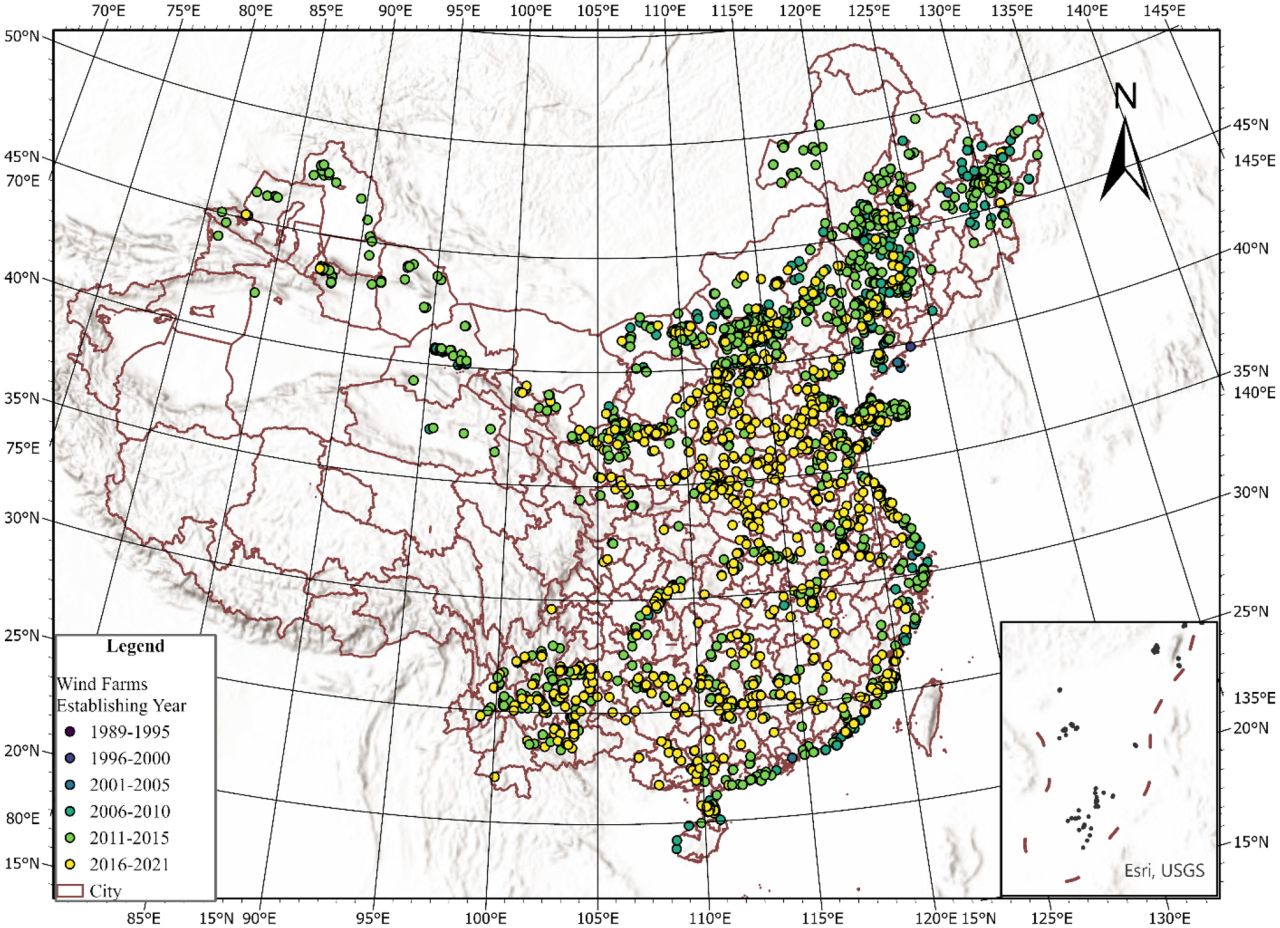
Distribution of wind farms in China. The dots (light to dark) represent wind farms installed in different years. The brown curve indicates the city’s administrative boundary. The figure depicts a total of 2,404 wind farms from the original dataset, with the earliest installation date in 1994 and the latest in 2021. Figure produced using ArcGIS Pro 3.2 (https://www.esri.com/en-us/arcgis/products/arcgis-pro/overview).

**Figure 2:**
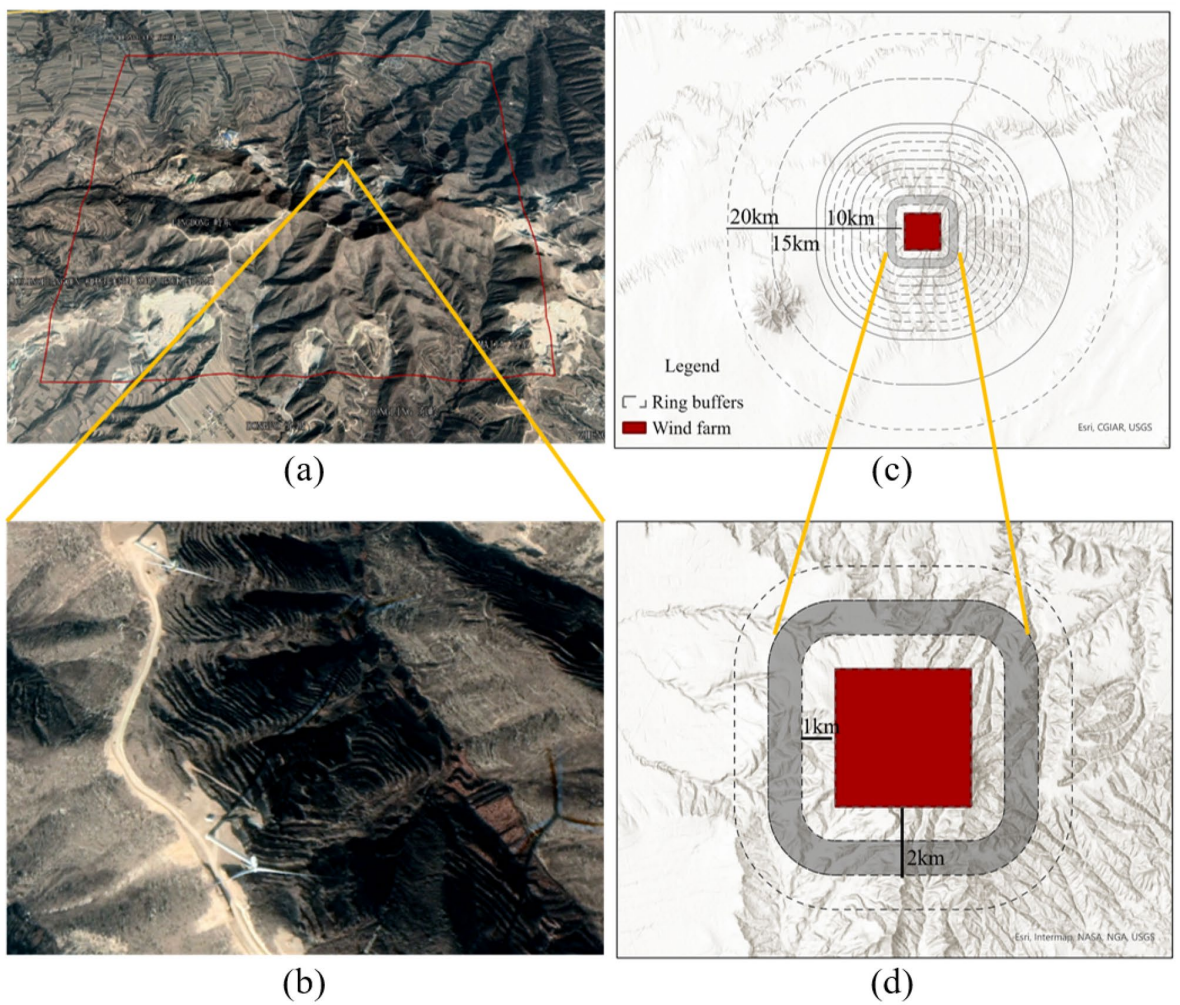
The illustration of field design of empirical evaluation for wind farms. Notes: Examples of wind turbine matrixes in wind farms (**a**) and satellite imagery of individual wind turbines (**b**) are provided in images (**a**, **b**). The red square indicates the area of inference. Images (**a**, **b**) were obtained from Google Earth (version 7.1.5.1557). The experimental design pattern is displayed in the vertical view (**c**, **d**). The experimental annuli were divided along the radius to the center of a wind turbine using dashed circles and curves. The method used to calculate changes in PBP around wind farms is illustrated in images c and d. The mean values of several PBP indicators within a radius of 1 km, 1–2 km, …, 9–10 km, 10–15 km, and 15–20 km from the center of the wind farm were computed for each wind farm (c). Specifically, the mean value of the PBP indicators was computed in an area consisting of 1 km and 2 km from the center of the wind farm, excluding the area within 1 km (the shaded region) to illustrate the approach (d). This approach enabled the comparison of the marginal variation of indicator values within each distance bin. Figure produced using ArcGIS Pro 3.2 (https://www.esri.com/en-us/arcgis/products/arcgis-pro/overview).

Previous studies on the impact of wind farms on vegetation dynamics have relied heavily on numerical simulation models due to data limitations. Such studies are often restricted to single geological types^19,20^, localized areas^41–43^, and/or short observation intervals^42^. This study uses nationwide data on the installation of 2,404 wind farms (Fig. 1) and 108,361 wind turbines in China and monthly remote sensing data on PBP indicators matched with 12 distance buffer zones for each wind farm from January 2000 to October 2022, totaling 7,904,352 month-by-buffer-zone samples (Fig. 2). We assessed the influence of China's rapidly expanding wind farm initiative on vegetation growth while estimating the heterogeneous effects on PBP and carbon sequestration, taking into account controls for natural, environmental, and anthropogenic factors. We examine ten PBP indicators: the normalized differential vegetation index (NDVI), enhanced vegetation index (EVI), the fraction of absorbed photosynthetically active radiation (FPAR), leaf area index (LAI), gross primary productivity (GPP), net photosynthesis (NP), net primary productivity (NPP), percentage of tree cover (PTC), percentage of non-tree vegetation cover (PNTV), and percentage of non-vegetation cover (PNV). We also assess the impact of wind farm construction on forest carbon sinks and economic value loss. Using the accumulation volume expansion method, we estimate the total carbon sink of forest trees based on the Ninth National Forest Inventory of China. We then calculate the forest carbon sink loss within the 20 km range of wind farms and employ the carbon tax method, utilizing the widely used Swedish carbon tax price of 150 USD/t of carbon, to evaluate the economic value loss of carbon sinks. Our findings highlight that wind power development has had profound ecological consequences.

This paper makes three primary contributions. Firstly, it provides a systematic evaluation of the externalities of wind turbines based on the wind power construction dataset and geographic information on PBP. The wind power construction China serves as a natural experiment to evaluate potential negative externalities of wind power on PBP within a 10 km buffer zone. Secondly, we evaluate the temporal dynamic effects of wind turbine construction and captures their heterogeneous effects on season, elevation, land types, and other characteristics. Thirdly, we discuss the potential negative contributions of increasing wind power to low-carbon transformation. Specifically, we estimate the total carbon sink and economic value loss of forest trees from wind turbine construction using the cumulative volume expansion and carbon tax method, respectively, which provides a reference point for policymakers to consider the sustainability of vegetation protection and the ecological environment and achieve low-carbon transformation goals.

## Results

### Changes in PBP due to wind farm construction

**Figure 3:**
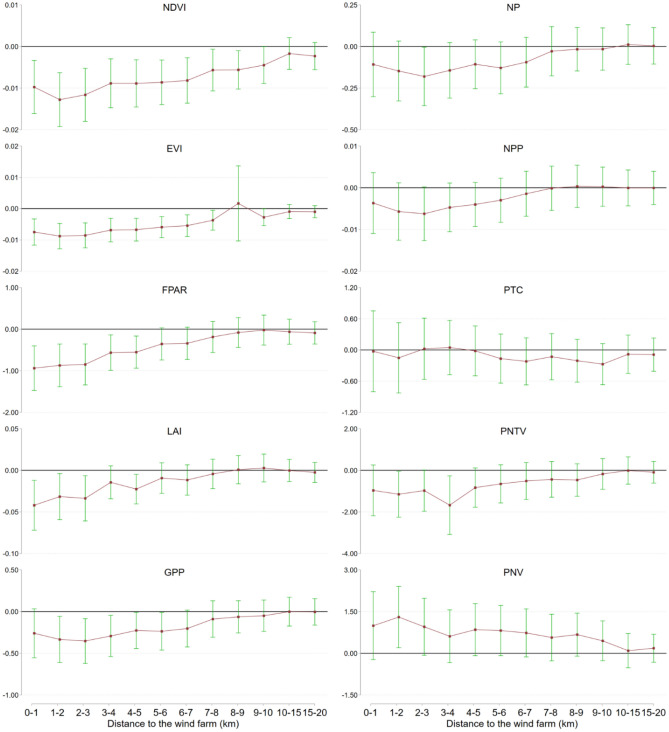
Average impacts of wind farm on PBP by distance. Notes: The graphs depict regression results for 10 PBP indicators, including the normalized difference vegetation index (NDVI), enhanced vegetation index (EVI), fraction of photosynthetically active radiation (FPAR), leaf area index (LAI, %), gross primary product (GPP, gC/m^2^), net photosynthesis (NP, gC/m^2^), net primary productivity (NPP, kg*C/m^2^), percentage of tree cover (PTC, %), percentage of non-tree vegetation (PNTV, %), and percentage of non-vegetation (PNV, %). The red points represent point estimators, and the green spikes show the 95% confidence intervals.

Wind farms could impact 0.08% of China’s terrestrial land area, or approximately 755,216 km^2^ if the impacts extend 10 km from each turbine, which represents an enormous spatial footprint that regional biodiversity threat maps and renewable energy plans fail to recognize. Figure 3 and Supplementary Table 4 present the findings on the impact of wind farms on PBP, measured by a range of indicators across varying distance buffers from 1 to 20 km. Estimations from Eq. (1) indicate the relationship between wind farms and PBP is generally U-shaped. The NDVI within 1 km of the wind farms exhibits a significant decrease of 0.0097 (*P* < 0.01; 95% CI − 0.0161 to − 0.0033), followed by declines of 0.0128 (*P* < 0.001; 95% CI − 0.0193 to − 0.0063) and 0.0116 (*P* < 0.001; 95% CI − 0.0180 to − 0.0052) when the distances are extended to 2 km and 3 km, respectively. The negative effect gradually diminishes and reaches − 0.0089 (*P* < 0.01; 95% CI − 0.0147 to − 0.0030) at around 4 km and is gone by 15 km. The weak U-shaped relationship remains consistent across the set of outcome variables. The negative impact of wind farms on EVI is significant within 1 km to 8 km, with a peak at 2 km and a maximum decrease of − 0.0088 (*P* < 0.001; 95% CI − 0.0128 to − 0.0047). Similarly, the impact on the FPAR is significant within 1 km to 7 km, peaking at 1 km, with a maximum decrease of − 0.0094 (*P* < 0.01; 95% CI − 0.0147 to − 0.0040) of a percentage point. The negative impact on the LAI is significant within a range of 1 km to 3 km, peaking at 1 km, with a maximum decrease of − 0.042 (*P* < 0.01; 95% CI − 0.0719 to − 0.0121). Finally, the negative impact on GPP is significant within a range of 1 km to 7 km, peaking at 3 km, with a maximum decrease of − 0.0004 kg*C/m^2^ (*P* < 0.01; 95% CI − 0.0006 to − 0.0001). Certain PBP indicators show less consistent and pronounced variation after wind farm construction, including NPP, NP, and PTC.

### Dynamic effects of wind farms

**Figure 4:**
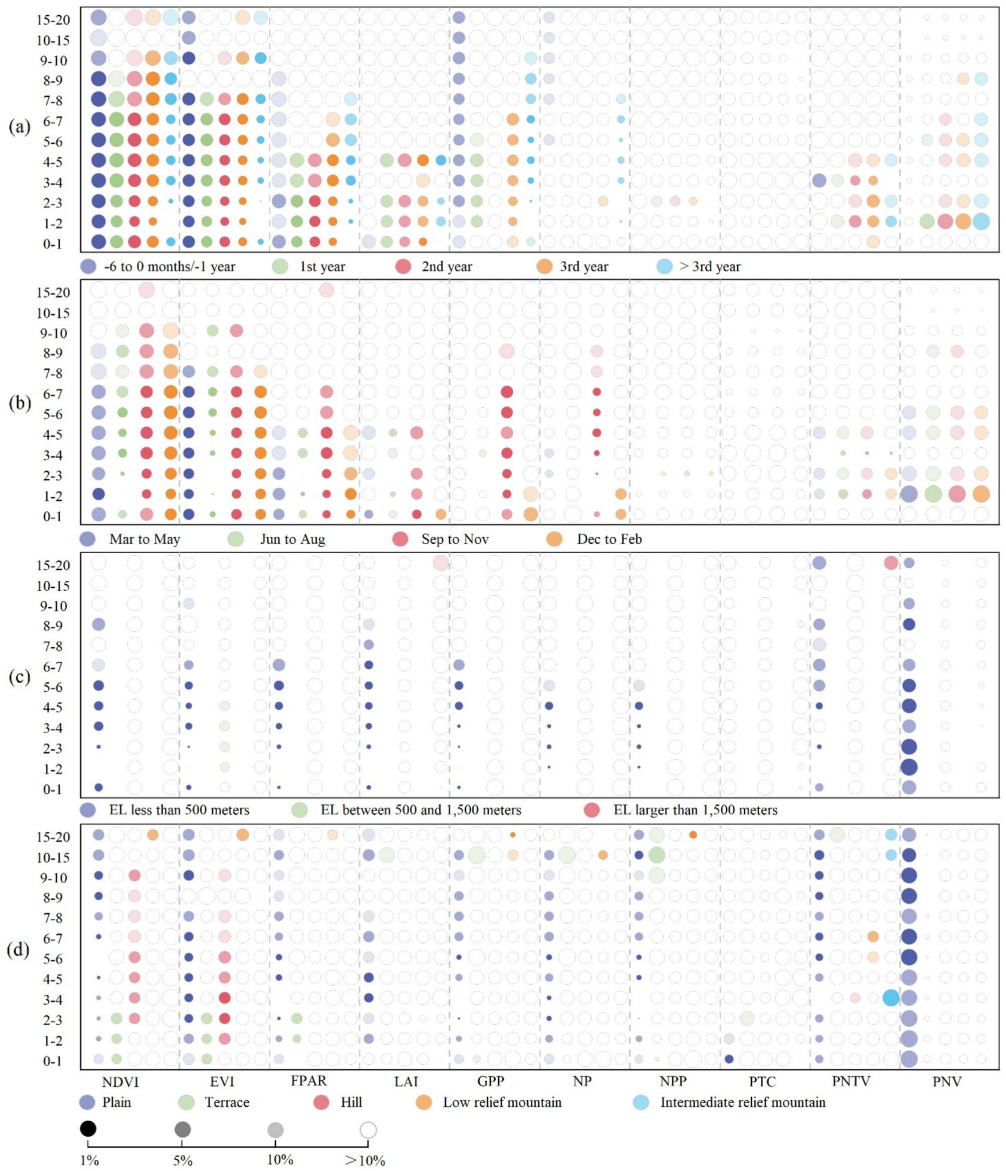
Heterogeneous effects of wind farm on PBP as a function of distance. Note: Vertical axis represents each distance range in kilometers and the horizontal axis represents each index of PBP. (**a**) Depicts the dynamic effects of wind farm on PBP by distance. For each indicator, estimates of five temporal phases are reported by distance: “ − 6 to 0 months” or “ − 1 year,” indicating half a year or one year prior to full wind farm installation; “1st,” “2nd,” “3rd,” and “ > 3 year,” representing one, two, three years, or more than three years post full installation of the wind farm. (**b**) Illustrates the seasonal heterogeneous effects of wind farm on PBP. For each indicator, estimates for the “post” phase in subsamples of the four seasons are reported by distance: “March to May” (Spring), “June to August” (Summer), “September to November” (Autumn), and “December to February” (Winter). (**c**) Represents the heterogeneous effects of wind farm elevation on PBP. For each indicator, estimates for the “post” phase in subsamples of three elevation (EL) ranges are reported by distance: “EL less than 500 m,” “EL between 500 and 1,500 m,” and “EL greater than 1500 m.” (**d**) Denotes the heterogeneous effects of wind farm land type on PBP. For each indicator, estimates for the “post” phase in subsamples of five different land types are reported by distance: “Plain,” “Terrace,” "Hill," "Low relief mountain," and "Intermediate relief mountain." Different bubbles represent normalized [0,1] values of estimated coefficients based on different plant indicators. The coefficients of all heterogeneity groups and 12 zones for each indicator are normalized as one group (and separated by dashed lines), so the size of the coefficients within each group is comparable, while bubbles between indicators are not comparable, and negative coefficients will inevitably have smaller bubbles than positive ones. Bubble color transitions from dark to light signify varying levels of statistical significance; darkest representing significant at 1%, followed by 5%, and 10%; blank indicates non-significance at the 10% statistical level. This figure reveals the heterogeneity of the wind farm’s interstitial effects on each indicator, the differences in distance, and the relative size variations of the coefficients. All regressions include the same explanatory variables, including NDVI, EVI, FPAR, LAI, GPP, NP, NPP, PTC, PNTV, and PNV. Different shades from black to light represent different levels of statistical significance, with black representing a 1% level of statistical significance. The absolute values of the specific effects can be found in the corresponding Supplementary tables.

Figure 4a and Supplementary Table 5 display the changes in PBP before and after the construction of wind farms by plotting the event-study coefficients estimated from Eq. (2) in the methods section. Figure 4a indicates that the PBP changes within 6–12 months before the wind farm begins operations, likely due to related constructions such as preparing the site, installing infrastructure such as roads and transmission lines, and erecting the wind turbines. PBP decreases after the installation, consistent with expectations. In the first year after the construction, NDVI declined by approximately 0.0105 for proximate wind farms. These results remain consistent outside the 3-year evaluation period.

Figure 4 indicates that the construction and operation of wind turbines may have long-term, increasing impacts on plant biodiversity. A potential cause is the impact of human activities related to road construction, building construction, and tourism development^44^. Turbines may also affect the distribution and ecological niche of plants. The noise and vibration turbines generate may damage vegetation and soil. Changes in air flow caused by the rotation of wind turbines may affect the climate and precipitation patterns of nearby areas. In addition to noise, light pollution and landscape alterations may negatively affect plants over time.

### Heterogeneous effects by season

Figure 4b and Supplementary Table 6 reveal that the negative impact of wind farms on plant communities predominantly occurs during the summer (June–August) and autumn (September–November) months, compared to the baseline. In these periods, the establishment of wind turbines reduces the FPAR component of vegetation by an average of 0.9896% (*P* < 0.05) and 0.7190% (*P* < 0.01) at 4 km distance, respectively. In contrast, the effect is only 0.4457% (*P* < 0.10) and 0.3429% (*P* < 0.10) during the spring (March–May) and winter (December–February) months. Similarly, the seasonal response of GPP to wind turbine construction is statistically significant only in the summer and autumn months, whereas the seasonal response of NP is significant only in autumn.

Existing literature indicates that the construction of wind farms alters the growth environment of plant communities by affecting factors such as light, temperature, humidity, and soil conditions. The rotation of wind turbines generates strong winds, resulting in leaf damage and increased evaporation. Additionally, the establishment of wind farms can disrupt the surrounding water cycle, potentially leading to soil aridity or excessive moisture. The most significant impacts of changes in the water cycle and light conditions occur during the summer and autumn seasons, as plants have the highest demand for water and light in these seasons and thus the weakest adaptability to these effects^45–47^. In comparison, the impact during the spring and winter seasons is relatively minor due to lower plant growth and reproductive demands for water and light, and a reduced influence of wind farms during these periods^24,48^. This result has significant implications for the planning and management of wind farm construction to minimize the negative externality to the surrounding natural environment^19,30,49^.

### Heterogeneous effects by elevation

Elevation is a crucial factor influencing the impact of wind farms on vegetation^50^, as Fig. 4c and Supplementary Table 7 suggest. Compared to the reference baseline regression, the construction of wind farms impacts plant communities at elevations lower than 500 m, but not at higher elevations. For plant communities below 500 m, the establishment of wind turbines reduces the FPAR component of vegetation by an average of 1.2792% (P < 0.01) at a distance of 4 km. This effect is only 0.1350% and 0.4287% for plant communities at elevations between 500 and 1,500 m and elevations above 1,500 m, respectively, and is statistically insignificant. Similarly, the installation of wind turbines only influences the GPP and NP of plant communities at altitudes lower than 500 m, while this effect on communities above 500 m is not significant.

At lower elevations, plant communities are likely to be closer to wind turbines and more exposed to strong winds. The rotation of wind turbine blades may generate more disturbances to vegetation, potentially causing tree swaying or leaf damage, subsequently adversely affecting vegetation growth^51,52^. Moreover, at lower elevations, higher relative humidity, fog, and clouds are more common, which increase moisture and humidity on the plant leaf surfaces, thus elevating the risk of plants swaying in high-speed winds^19,53^. In addition to the more dispersed distribution of plant communities at higher elevations, harsher climatic conditions and different vegetation types may result in more resilient ecosystems and a more robust response to external disturbances, including wind farms.

### Heterogeneous effects by land types

In our study, land type strongly influenced the impact of wind turbine installation on vegetation FPAR components within a few kilometers, as Fig. 4d and Supplementary Table 8 suggest. The construction of wind farms had a stable impact on plant communities in plain land types but not plateaus, hills, low-relief mountains, or medium-relief mountains. The establishment of wind turbines led to an average reduction of 1.4859% (*P* < 0.01) in vegetation PAR components at 4 km, and persisted up to 20 km for plain land type. For plateau, the impact of wind turbines was only present within areas smaller than 3 km, with a coefficient size of 0.6969% (*P* < 0.05), while the corresponding response in plant communities of plain land type at the same distance was 1.4034% (*P* < 0.01). The effects of wind turbine installation on gross primary production and net photosynthesis of plant communities were only significant in plain land type, with effect sizes of 0.8392 gC/m^2^ and 0.4822 gC/m^2^ at 4 km, respectively. No significant impact was observed in plant communities of other land types.

Differences in species composition and adaptability of plant communities in different land types may explain the differing impact of wind farms. Plains often have large areas of farmland and grassland, with a relatively simple vegetation structure, making the plant communities more vulnerable to external disturbances and damage. By contrast, plateaus, hills, low and medium-relief mountains have a more complex vegetation structure, including a higher diversity of shrubs, herbaceous plants, and trees that are better adapted to the effects of wind farms^19,54^. Furthermore, plain land types generally have flat terrain and relatively deeper soils, which facilitate the interception of water and nutrients by equipment and structures associated with wind farms, potentially causing damage to the original vegetation^19,55,56^. Plateaus, hills, low-relief mountains, and medium-relief mountains typically exhibit greater elevation differences and slopes, and the plant communities within these areas tend to be more uniform, making it more difficult for wind farm equipment and structures to negatively impact the vegetation^57,58^. These factors contribute to the increased vulnerability of plant communities in plain land types to the adverse effects of wind farm construction, while plant communities in plateaus, hills, low and medium-relief mountains are more resilient to such impacts^59–61^.

## Discussion

Our study focuses on the underrepresented implications of wind energy on vegetation dynamics. Results reveal that wind farm construction has a significantly negative effect on PBP, and the extent of these impacts varies across indicators. On average, wind farm construction leads to a decrease in NDVI and EVI within a range of 1–10 km, from 0.0097 to 0.0045 and from 0.0075 to 0.0028, respectively. It also leads to a decrease in FPAR and GPP within a range of 1–7 km, from 0.0094% to 0.0034% and from 0.0003 to 0.0002 g*C/m^2^, respectively. The study also shows that wind farm effects follow a weak U-shaped relationship with several indicators, with the effects on NDVI and EVI reaching their maximum at 2 km. On average, wind farms result in a decrease in non-tree vegetation cover, and an increase in the proportion of non-vegetation cover, with no significant impact on tree cover, suggesting that vegetation with lower height, such as grassland and shrubs, is more vulnerable.

Our dynamic analysis indicates that certain diversity indicators exhibit a significant decrease within six months before the wind farm operation, suggesting that the construction process itself has some detrimental effects on vegetation. Furthermore, the negative impacts persist and even increase for more than three years after the wind farm become operational. Heterogeneity analysis reveals that the effects of wind farms on plant communities varied according to season, altitude, and land type. Negative impacts on vegetation photosynthesis and production are more pronounced during the summer and autumn months, at lower altitudes, and in plain land types. Different types of plants may exhibit distinct responses under these specific conditions. Regarding the underlying reasons, firstly, summer and autumn often experience higher temperatures and lower precipitation levels. Plants during these periods might be in crucial growth stages, such as flowering or fruit maturation, rendering them more sensitive to environmental factors. Furthermore, summer and autumn are likely peak operational times for wind farms, as increased electricity generation is required during high-temperature weather. Elevated wind turbine activity could lead to more pronounced environmental disturbances. Secondly, lower-altitude regions typically feature warm, arid climates and fertile soils, though they might also be more susceptible to erosion and nutrient loss. Moreover, the plant communities in these areas might be more prone to disturbances due to their adaptation to relatively stable environmental conditions. Lastly, ecosystems in plain regions might be comparatively simple, lacking sufficient biodiversity to withstand environmental disruptions. They often face greater susceptibility to anthropogenic land use changes, such as agriculture and urbanization, which could interact synergistically with wind farm development. These findings highlight the importance of considering the heterogeneous effects of wind farm construction on the surrounding natural environment in planning and management efforts to minimize negative impacts.

While the longstanding damage caused by coal-fired power plants on vegetation has been extensive and alarming, from an economic perspective, the research findings are also valuable to assess the damage to vegetation productivity and corresponding environmental and economic costs from the construction of wind farms in China. According to the *United Nations Framework Convention on Climate Change*, carbon sinks reduce the amount of carbon dioxide in the atmosphere by absorbing and storing carbon dioxide through photosynthesis in forest trees^62–65^. Forest carbon sequestration is more sustainable and stable than other methods of carbon reduction, lasting for hundreds of years, and can effectively mitigate global climate change^66^. However, Holst found that the growing environment of forest trees can influence the capacity of carbon sequestration, and the external effects, such as forest management, forest land use, and other human interference can alter the quality and areas of forest trees, resulting in variation in forest carbon sink^67^. Thus, we calculate the change of forest areas due to the construction of wind farms with the estimated coefficients of the PTC and then compute the change of forest carbon sink to assess the economic value of lost forest carbon sink due to wind farms^68^.

Using the accumulation volume expansion method, we estimate the total carbon sink of forest trees. The procedure (shown in Eq. (1) below) involves calculating forest biomass carbon sequestration based on the volume expansion coefficient, converting the biomass into biomass dry weight using volume density, and determining carbon sequestration by applying the rate of carbon content. The carbon sequestration amounts of understory plants and forest land are then computed using the respective carbon conversion coefficients, resulting in the total carbon sink of forest trees.

1 $$\begin{aligned} C_{F} & = \left( {S \times C} \right) + \alpha \left( {S \times C} \right) + \beta \left( {S \times C} \right) \\ C & = V \times \delta \times \rho \times \gamma \\ \end{aligned}$$

where $C_{F}$ is the total carbon sink of forest trees; $S$ is the forest area in the buffer zone; $C$ is the forest carbon density; $\alpha$ is the understory plants carbon conversion coefficient (taking the value of 0.195); $\beta$ is the forest land carbon conversion coefficient (taking the value of 1.244); $V$ is the per unit area of forest volume (taking the value of 79.66m^3^/hm^2^); $\delta$ is the volume expansion coefficient (taking the value of 1.90); $\rho$ is the volume density (taking the value of 0.5); $\gamma$ is the rate of carbon content (taking the value of 0.5). This method utilizes the per unit area of forest volume data from the Ninth National Forestry Survey, which reports a standard value of 79.66 m^3^ per hectare for China, along with conversion coefficients specified by the Intergovernmental Panel on Climate Change (IPCC)^69^. We obtain the amount of forest carbon sink loss in each buffer zone within the 20 km range of the wind farm by calculating the amount of change in the percentage of tree cover, multiplied by unit area accumulation in China. Furthermore, we employ the carbon tax method to calculate the economic value loss of carbon sinks based on Swedish carbon tax price of 150 USD/t.

According to Supplementary Table 4, within the study area of 0–20 km from the wind farm, the average forest carbon sink decreases by 12,034.21 tons after construction, and the average economic loss per wind farm of carbon sink reaches $1.81 million. The largest decrease in tree cover is observed in the 9–10 km buffer, with an average decrease of 0.27%, and the average carbon sink loss reaches $225,715. However, the average tree cover increases by 0.05% in the 3–4 km buffer, and the average carbon sink economic value increases by $14,027. Overall, except for the average carbon sink increase in the 2–4 km buffer, other buffer zones are showing different degrees of carbon sink loss. According to Supplementary Tables 5, 6, 7 and 8, the loss of forest carbon sink in one year prior to the construction of wind farms is the smallest, with an average carbon sink reduction of 2109.52 tons and an average economic loss of $316,427.44. The loss is the largest after three years of construction, with an average carbon sink reduction of 18,158.28 tons, and an average economic loss of $2.72 million. The total reduction of carbon exceeds 10,000 tons per year, with the largest reduction of 14,284.93 tons and economic loss of $2.24 million in spring, and the smallest reduction in autumn. Wind farms at greater elevation areas have the largest impact on the carbon sink, with an increase of 11,539.22 tons and an average economic gain of $1.73 million, while wind farms at intermediate elevation areas have the smallest impact on the carbon sink with a reduction of 9,622.30 tons and an average economic loss of $1.44 million. Wind farms in plains, hills, and low relief mountains are leading to a loss of forest carbon sinks while wind farms in plateaus and medium-relief mountains are not. The greatest loss is in the hilly areas, amounting to 52,657.06 tons and an average economic loss of $7.90 million.

While coal-fired power plants have historically wreaked havoc on vegetation ecosystems, exacerbating habitat loss and degradation, the loss of economic value of carbon sinks reflects the negative spillovers caused by wind power, which calls for more attention to the impact of green energy on regional ecology while expanding the wind power industry and developing clean energy. To mitigate the potential threats of renewable energy deployment to vegetation ecosystems, a comprehensive monitoring system is needed to assess the impacts of wind farms on plant communities over time. Regular vegetation surveys, remote sensing, and other monitoring techniques should be used to track changes in vegetation succession and distribution. Landscape planning should be conducted before wind farm construction to identify areas of particular importance for plant biodiversity and to avoid placing wind farms in those areas. Offset measures, such as reforestation, habitat restoration, and the creation of wildlife corridors, can be implemented to mitigate the negative externalities of wind farm development. However, it’s crucial to contextualize these findings within the broader initiative to transition from fossil fuels to renewable energy, a move that comes with its own set of significant environmental benefits. Future research should examine the ecological requirements of plant species and develop balanced mitigation strategies for energy transition.

## Methods

### Data

The data primarily consists of two parts. The first part includes wind power data, collected from the National and Provincial Development and Reform Commission, the Energy Bureau, wind power project public bidding documents, wind power project completion environmental impact acceptance reports, and the China Clean Development Mechanism’s official website. The wind power data includes all completed and operational wind farms as of 2022 at the county level, with detailed information on construction completion dates, installed capacity, and the longitude and latitude coordinates of the center of each wind farm. We collected data from 2404 wind farms built between 1994 and 2022, as shown in Fig. 1. Wind farms with incomplete information on construction time and installation data were excluded from the final sample.

We utilized the geographic information system (GIS) to determine the land surface area covered by the turbines of each wind farm in a two-step process. Firstly, we geocoded the latitude and longitude of each wind farm by combining its address with high-resolution remote sensing images. We determined the exact location of each wind farm based on the recorded address and the visibility of wind turbines in the remote sensing images. Secondly, we divided the national land surface into 2 km × 2 km grids. Because the typical distance between two turbines is 0.5 km, and wind turbines are usually equally distributed within a given wind farm, we calculated the number of grids occupied by the wind turbines by dividing the total number of turbines in each wind farm by 16. The grids closest to the wind farm’s location are designated as the land parcels occupied by the wind farm, and we considered the outward boundaries of the grids as the spatial boundaries of each wind farm.

The second dataset includes plant biomass production (PBP) indicators from Google Earth Engine. Ten indices were used to measure PBP, including NDVI, EVI, FPAR, LAI, GPP, NP, NPP, PTC, PNTV, and PNV. NDVI and EVI assess vegetation growth and cover, with values ranging from − 1 to 1. FPAR measures vegetation’s light energy efficiency, with values from 0 to 1 (normalized to [0, 100] in analysis). LAI indicates vegetation growth and canopy structure, with values from 0 to 1. GPP and NP represent organic carbon fixation and net photosynthesis, respectively. NPP measures organic carbon remaining in plants, with GPP and NPP values typically ranging from 0 to 0.3. PTC, PNTV, and PNV describe the percentage of tree cover, non-tree vegetation cover, and non-vegetation cover, respectively, with values from 0 to 100. Supplementary Table 1 provides detailed definitions.

The Terra satellite provides data for the years 2000–2022, using the MODIS (Moderate Resolution Imaging Spectroradiometer) remote sensing data product. We measure NDVI and EVI using the 16-day synthetic vegetation index product MOD13Q1.061, while FPAR and LAI use the 8-day synthetic land leaf area index product MOD15A2H.061. Similarly, we measure GPP and NP using the 8-day synthetic primary productivity product MOD17A2H.006, while NPP is measured using the 1-year synthetic primary productivity product MOD17A3HGF.006. To determine the coverage rate of planting areas, we use the annual data product MOD44B.006 to measure the PTC, PNTV, and PNV indices. Since the initial data covers a global scale, we use the Java Script programming platform to spatially locate the longitude and latitude coordinates of the centers of the 2,404 wind farms and obtain the monthly vegetation data based on specific wind farm locations within each buffer zone.

### Empirical strategy

We divide the study area within a 20 km from the center of each wind farm into 12 buffer zones based on distance, including 1 km, 1–2 km, 2–3 km, 3–4 km, 4–5 km, 5–6 km, 6–7 km, 7–8 km, 8–9 km, 9–10 km, 10–15 km, and 15–20 km from the center of the wind farm, resulting in a total of 3288 month-by-buffer-zone observations (i.e. 274 months from January 2000 to October 2022 multiplied by 12 buffer zones) for a single wind farm. To eliminate the effects of multiple turbine expansions on the analysis, we limit our consideration to 1,936 wind farms that were built after 2002 and have only undergone a single installation (Supplementary Table 2). Consequently, we have a total of 6,365,568 observations for analysis.

### Fixed effects approach

We implement a high-dimensional fixed effects approach to estimate the average effect of wind farm construction on PBP under a certain distance buffer zone, specified as follows:

2 $$Y_{it}^{k,d} = \theta^{k,d} post_{it} + {\mathbf{X}}_{it}^{\prime} {{\varvec{\upgamma}}}^{k,d} + \delta_{j} \times \eta_{y} + \tau_{m} + \mu_{g} + \varepsilon_{it} ,$$

where $Y_{it}^{k,d}$ denotes one of the $k$ PBP indicators of a distance buffer zone $d$ of wind farm $i$ at month $t$. $post_{it}$ equals 1 if it is after the wind farm installation and 0 otherwise. ${\mathbf{X}}_{it}^{\prime}$ is a vector of control variables that may affect PBP, including the number of wind turbines, wind power capacities of the wind farm, precipitation, temperature at two meters above the ground, nighttime light, and elevation. In particular, precipitation and temperature are good measures of surrounding natural or climatic conditions to exclude the effects of global warming issues such as extreme weather events on the vegetation growth; nighttime light data has garnered considerable attention in recent scientific literature as a robust and informative proxy for assessing local economic development^70–72^. This approach allows researchers to capture the extent of anthropogenic activity and effectively control for its associated implications on human–environment interactions^73,74^, particularly concerning vegetation around wind farms. Supplementary Table 1 provides detailed descriptions of all control variables. $\theta^{k,d}$ is the main coefficient of interest, which measures the difference of mean values of a PBP indicator $k$ before and after wind farm installation within buffer zone $d$, and ${{\varvec{\upgamma}}}^{k,d}$ denotes the vector of coefficients for the control variable ${\mathbf{X}}$. In the presented equation, county-by-year fixed effects ($\delta_{j} \times \eta_{y}$) not only represent unobserved attribute changes within the counties *(j*) housing wind farms but also precisely capture and control the economic dynamics of these regions. These dynamics encompass, but are not limited to, variations in Gross Domestic Product, industrial output, and traffic indicators. The comprehensiveness and granularity of these fixed effects effectively mitigate any natural or anthropogenic confounding factors in the estimation of wind farm impacts. Consequently, their incorporation into the study not only bolsters confidence in the accuracy of the estimated benefits of wind farms but also ensures the reliability and precision of the analysis in identifying the impacts of wind farms on local economies and environments. $\tau_{m}$ represents month-of-year fixed effects, capturing any monthly characteristics or seasonal effects such as changes in local wind speed and direction. $\mu_{g}$ represents land type fixed effects, which control for unobservables under different topographic features. We considered seven land type categories, including the plain, terrace, hill, low relief mountain, intermediate relief mountain, high relief mountain, and extremely high relief mountain. $\varepsilon_{it}$ is the idiosyncratic error term. We repeatedly estimated Eq. (2) in the methods section for all distance buffer zones ($d = 1,2, \ldots ,12$) and for all PBP indicators ($k = 1,2, \ldots ,10$). All regressions employ identical specifications. We also clustered the standard errors at the wind farm level, which accounts for correlations between observations around the same wind farm.

### Dynamic treatment effect

To examine the dynamic effects of wind farm construction, based on Eq. (2), we replace the dummy $post_{it}$ with a set of dummy variables indicating different time periods to explicitly estimate the impact of wind farms six months before, the first year, the second year, the third year, and more than three years after the installation of the wind farm. The dynamic model is specified as follows:

3 $$Y_{it}^{k,d} = \theta_{ - 1}^{k,d} lag_{1it} + \mathop \sum \limits_{q = 1}^{4} \theta_{ + q}^{k,d} lead_{qit} + {\mathbf{X}}_{it}^{\prime} {{\varvec{\upgamma}}}^{k,d} + \delta_{j} \times \eta_{y} + \tau_{m} + \mu_{g} + \varepsilon_{it} ,$$

where $Y_{it}^{k,d}$ denotes one of the $k$ PBP indicators of a distance buffer zone $d$ of wind farm $i$ at month $t$. $lag_{1}$ is a dummy that equals 1 if it is at least six months before installation and 0 otherwise; $lead_{q}$ is a dummy indicating one year ($q = 1$), two years ($q = 2$), three years ($q = 3$), and more than three years ($q = 4$) after installation. $\theta_{ - 1}^{k,d}$ and $\theta_{ + q}^{k,d}$’s are coefficients measuring the dynamic impacts of wind farm installation. The remaining terms are identical to Eq. (2). Since NPP, PTC, PNTV, and PNY are measured by year, $lag_{1}$ indicates one year, other than half a year, before installation for the regressions of these four indicators. For all regressions, “more than half a year (or one year) before installation” serves as the reference group.

### Heterogeneous effects

We also estimate Eq. (4) below with different groups of subsamples to explore the heterogeneous effects across season, elevation, and land type.

4 $$Y_{it}^{k,d,h} = \theta^{k,d,h} post_{it} + {\mathbf{X}}_{it}^{\prime} {{\varvec{\upgamma}}}^{k,d,h} + \delta_{j} \times \eta_{y} + \tau_{m} + \mu_{g} + \varepsilon_{it} ,$$

where $Y_{it}^{k,d,h}$ denotes one of the $k$ PBP indicators within a distance buffer zone $d$ of wind farm $i$ at month $t$. $h$ denotes one of the three groups of subsamples. For seasonal heterogeneous effects ($h = 1$), we considered four subsamples—spring (March to May), summer (June to August), autumn (September to November), and winter (December to February), which results in 480 (10 × 12 × 4) regressions and $\theta^{k,d,h}$’s. For heterogeneous effects of elevation ($h = 2$), we considered three subsamples—elevation less than 500 m, elevation between 500 and 1,500 m, and elevation larger than 1,500 m, which results in 360 (10 × 12 × 3) regressions and $\theta^{k,d,h}$’s. For heterogeneous effects of land type ($h = 3$), we considered five subsamples—plain, terrace, hill, low relief mountain, intermediate relief mountain, resulting in 600 (10 × 12 × 5) regressions and $\theta^{k,d,h}$’s.

We determine which subsample of the elevation group and land type group each wind farm belongs to by the mean values of elevation (DEM) and topographic (Geomor) indicators within the 1 km buffer zone for the wind farm. For the seasonal subsample, it is determined directly from the specific month recorded for each observation. In addition, in the land type group, the five available subsamples did not cover the entire sample. Due to the small number of wind farms distributed in the two types of land type—high relief mountain and extremely high relief mountain—we did not include estimates of these two subsamples.

### Supplementary Information

Supplementary Tables.

## Data Availability

The datasets used and/or analysed during the current study are available from the corresponding author on reasonable request.
