# Supplementary material for: The impact of wind energy on plant biomass production in China
Source: Sci Rep. 2023 Dec 15;13:22366. doi: 10.1038/s41598-023-49650-9 (PMC10724281; doi:10.1038/s41598-023-49650-9)
Supplement: Supplementary file 1 — Supplementary Tables. [file 41598_2023_49650_MOESM1_ESM.docx]

**Supplementary Table 1. Variable definitions**

| **Variables** | **Full name** | **Definitions** | **Ranges** | **Precision** |
| --- | --- | --- | --- | --- |
| Panel A: Plant diversity indicators | | | | |
| NDVI | Normalized Differential Vegetation Index | Reflect the growth status and coverage of vegetation. | [-1, 1] | 16-day |
| EVI | Enhanced Vegetation Index | Reflect vegetation coverage, and is more sensitive to high biomass vegetation. | [-1, 1] | 16-day |
| FPAR | The Fraction of Absorbed Photosynthetically Active Radiation | The proportion of absorbable photosynthetic active radiation in photosynthetic active radiation. | [0, 100] % | 8-day |
| LAI | Leaf Area Index | Reflect the growth status and vegetation cover thickness of vegetation communities. | [0, 10] | 8-day |
| GPP | Gross Primary Productivity | The organic matter and energy fixed by vegetation through photosynthesis and entering the ecosystem. | [0, 300] g*C/m^2^ | 8-day |
| NP | Net Photosynthesis | The total amount of photosynthesis occurring in plants minus the amount of respiration. | [-300, 300] g*C/m^2^ | 8-day |
| NPP | Net Primary Productivity | The organic matter and energy fixed by vegetation through net photosynthesis and used for its own growth and reproduction. | [-3, 3] kg*C/m^2^ | 1-year |
| PTC | Percentage of Tree Cover | The percentage of each pixel covered by the tree canopy. | [0, 100] % | 1-year |
| PNTV | Percentage of Non-Tree Vegetation Cover | The percent of each pixel covered by non-tree vegetation canopy. | [0, 100] % | 1-year |
| PNV | Percentage of Non- Vegetation Cover | The percent of each pixel that is not covered by vegetation. | [0, 100] % | 1-year |
| Panel B: Other variables | | | | |
| Post | Post Wind Farm Installation | =1 if it is after the wind farm installation; =0 otherwise. |  |  |
| Turbines | Number of Wind Turbines | Current number of wind turbines in the wind farm. |  |  |
| Capacities | Wind Power Capacities | Current installed wind power capacities. |  |  |
| Precipitation | Precipitation | Monthly average of precipitation. | mm | 1-month |
| Temperature | 2-meter Temperature | Monthly average of air temperature at a height of 2 meters from the Earth’s surface. | ℃ | 1-month |
| Light | Nighttime Lights Remote Sensing | Refers to the intensity of nighttime lights, reflecting the industrial production, commercial activities and energy consumption and other activities of human society. | nW/cm^2^/sr | 1-year |
| DEM | Digital Elevation Model | Refers to the vertical distance from a point on the ground to a certain horizontal plane. | m | 2022 update |
| Geomor | Distribution of Geomorphology and Topography. | Refers to the relief morphology of the Earth’s surface. |  | 2009 update |

**Supplementary Table 2. Number of wind farms in the sample (N=2,404)**

|  | Installed only once | Installed more than once | Total |
| --- | --- | --- | --- |
| First installation posterior to 2002 | 1,936 | 442 | 2,378 |
| First installation anterior to 2002 | 7 | 19 | 26 |
| Total | 1,943 | 461 | 2,404 |

Note: To avoid the influence of multiple turbine expansions on the estimation results and given that the available plant diversity data started from 2000, wind farms built after 2002 and featuring only one turbine installation were included in the empirical models. Consequently, the regression analysis was conducted using a total of 1,936 wind farms.

**Supplementary Table 3. Summary statistics of selected variables**

| Variable | Obs. | Mean | Std. Dev. | Min. | Max. |
| --- | --- | --- | --- | --- | --- |
| Before installation (Post = 0) | | | | | |
| NDVI | 3,688,729 | 0.343 | 0 | -0.200 | 0.927 |
| EVI | 3,688,534 | 0.206 | 0.137 | -0.122 | 0.851 |
| FPAR | 3,313,684 | 27.844 | 18.818 | 0 | 154.025 |
| LAI | 3,313,684 | 0.784 | 0.871 | 0 | 12.945 |
| GPP | 3,332,978 | 14.008 | 14.015 | 0 | 87.209 |
| NP | 3,332,991 | 9.959 | 9.440 | -26.860 | 66.873 |
| NPP | 3,351,204 | 0.404 | 0.239 | 0.014 | 1.560 |
| PTC | 3,729,853 | 10.885 | 13.152 | 0 | 80.149 |
| PNTV | 3,716,688 | 49.067 | 14.326 | 0 | 90.591 |
| PNV | 3,729,853 | 39.972 | 19.343 | 0.784 | 99.446 |
| Turbines | 4,277,872 | 0 | 0 | 0 | 0 |
| Capacities | 4,277,872 | 0 | 0 | 0 | 0 |
| Precipitation | 3,166,087 | 52.053 | 64.712 | 0 | 719.700 |
| Temperature | 3,166,087 | 9.799 | 11.948 | -31.480 | 32.983 |
| Elevation | 4,276,295 | 851.621 | 774.470 | -25.000 | 3782.105 |
| Light | 3,474,358 | 0.136 | 0.950 | 0 | 68.641 |
| After installation (Post == 1) | | | | | |
| NDVI | 2,155,283 | 0.345 | 0.221 | -0.200 | 0.948 |
| EVI | 2,155,172 | 0.212 | 0.146 | -0.119 | 0.833 |
| FPAR | 1,929,082 | 29.100 | 19.732 | 0 | 126.428 |
| LAI | 1,929,082 | 0.809 | 0.895 | 0 | 10.349 |
| GPP | 1,929,134 | 15.247 | 15.490 | 0 | 88.257 |
| NP | 1,948,467 | 10.720 | 10.292 | -27.833 | 65.593 |
| NPP | 1,754,028 | 0.420 | 0.225 | 0.013 | 1.513 |
| PTC | 1,723,427 | 10.715 | 13.437 | 0 | 78.372 |
| PNTV | 1,716,372 | 48.719 | 14.803 | 0 | 89.368 |
| PNV | 1,723,427 | 40.512 | 19.815 | 0.731 | 99.594 |
| Turbines | 2,504,176 | 38.344 | 31.431 | 1 | 434 |
| Capacities | 2,504,176 | 62.207 | 53.691 | 1 | 700 |
| Precipitation | 1,681,049 | 53.198 | 66.053 | 0 | 650.691 |
| Temperature | 1,681,049 | 9.621 | 12.229 | -32.180 | 33.802 |
| Elevation | 2,520,549 | 803.684 | 751.606 | -25.000 | 3782.105 |
| Light | 1,604,042 | 0.374 | 1.588 | 0 | 69.395 |

**Supplementary Table 4. Average impacts of wind farm on plant diversity by distance.**

|  | (1) | (2) | (3) | (4) | (5) | (6) | (7) | (8) | (9) | (10) | (11) | (12) |
| --- | --- | --- | --- | --- | --- | --- | --- | --- | --- | --- | --- | --- |
|  | 0-1 km | 1-2 km | 2-3 km | 3-4 km | 4-5 km | 5-6 km | 6-7 km | 7-8 km | 8-9 km | 9-10 km | 10-15 km | 15-20 km |
| Panel A: NDVI (normalized difference vegetation index) | | | | | | | | | | | | |
| Post WF installation | -0.0097*** | -0.0128*** | -0.0116*** | -0.0089*** | -0.0089*** | -0.0086*** | -0.0082*** | -0.0057** | -0.0056** | -0.0045** | -0.0017 | -0.0023 |
|  | (0.0033) | (0.0033) | (0.0032) | (0.0030) | (0.0029) | (0.0027) | (0.0028) | (0.0026) | (0.0023) | (0.0023) | (0.0019) | (0.0017) |
| N | 371,333 | 375,510 | 378,003 | 379,844 | 380,814 | 381,970 | 382,898 | 382,898 | 383,540 | 383,754 | 384,824 | 385,466 |
| Adj. R^2^ | 0.7575 | 0.7644 | 0.7709 | 0.7768 | 0.7803 | 0.7837 | 0.7851 | 0.7875 | 0.7900 | 0.7923 | 0.7983 | 0.8034 |
| Panel B: EVI (enhanced vegetation index) | | | | | | | | | | | | |
| Post WF installation | -0.0075*** | -0.0088*** | -0.0086*** | -0.0069*** | -0.0067*** | -0.0059*** | -0.0054*** | -0.0037** | 0.0017 | -0.0028** | -0.0009 | -0.0010 |
|  | (0.0021) | (0.0021) | (0.0020) | (0.0019) | (0.0019) | (0.0017) | (0.0018) | (0.0016) | (0.0061) | (0.0014) | (0.0012) | (0.0010) |
| N | 371,333 | 375,510 | 378,003 | 379,844 | 380,814 | 381,970 | 382,898 | 382,898 | 383,248 | 383,754 | 384,824 | 385,466 |
| Adj. R^2^ | 0.7506 | 0.7585 | 0.7651 | 0.7720 | 0.7769 | 0.7807 | 0.7821 | 0.7847 | 0.5496 | 0.7895 | 0.7970 | 0.8030 |
| Panel C: FPAR (fraction of photosynthetically active radiation) | | | | | | | | | | | | |
| Post WF installation | -0.9384*** | -0.8702*** | -0.8482*** | -0.5636*** | -0.5522*** | -0.3558* | -0.3406* | -0.1872 | -0.0811 | -0.0221 | -0.0623 | -0.0910 |
|  | (0.2731) | (0.2608) | (0.2509) | (0.2169) | (0.1968) | (0.1953) | (0.1972) | (0.1907) | (0.1828) | (0.1835) | (0.1530) | (0.1363) |
| N | 351,154 | 356,284 | 360,032 | 362,960 | 365,316 | 367,470 | 368,398 | 369,898 | 371,540 | 374,004 | 377,824 | 381,966 |
| Adj. R^2^ | 0.6907 | 0.7084 | 0.7156 | 0.7243 | 0.7269 | 0.7281 | 0.7302 | 0.7314 | 0.7340 | 0.7355 | 0.7426 | 0.7488 |
| Panel D: LAI (leaf area index, %) | | | | | | | | | | | | |
| Post WF installation | -0.0420*** | -0.0316** | -0.0337** | -0.0145 | -0.0226** | -0.0093 | -0.0117 | -0.0043 | 0.0007 | 0.0028 | -0.0002 | -0.0025 |
|  | (0.0152) | (0.0141) | (0.0139) | (0.0101) | (0.0090) | (0.0093) | (0.0093) | (0.0090) | (0.0086) | (0.0085) | (0.0068) | (0.0062) |
| N | 351,154 | 356,284 | 360,032 | 362,960 | 365,316 | 367,470 | 368,398 | 369,898 | 371,540 | 374,004 | 377,824 | 381,966 |
| Adj. R^2^ | 0.6562 | 0.6950 | 0.6992 | 0.7204 | 0.7192 | 0.7170 | 0.7238 | 0.7215 | 0.7260 | 0.7253 | 0.7359 | 0.7421 |
| Panel E: GPP (gross primary product, g*C/m^2^) | | | | | | | | | | | | |
| Post WF installation | -0.2599* | -0.3338** | -0.3528*** | -0.2926** | -0.2260** | -0.2361** | -0.2033* | -0.0897 | -0.0636 | -0.0496 | -0.0012 | -0.0036 |
|  | (0.1497) | (0.1410) | (0.1364) | (0.1259) | (0.1101) | (0.1144) | (0.1126) | (0.1113) | (0.0984) | (0.0959) | (0.0879) | (0.0807) |
| N | 352,553 | 357,704 | 361,468 | 364,408 | 366,774 | 368,937 | 369,869 | 371,375 | 373,024 | 375,498 | 379,334 | 383,493 |
| Adj. R^2^ | 0.7314 | 0.7408 | 0.7490 | 0.7535 | 0.7583 | 0.7603 | 0.7613 | 0.7631 | 0.7649 | 0.7659 | 0.7702 | 0.7744 |
| Panel F: NP (net photosynthesis, g*C/m^2^) | | | | | | | | | | | | |
| Post WF installation | -0.1078 | -0.1474 | -0.1803** | -0.1435* | -0.1067 | -0.1287 | -0.0941 | -0.0284 | -0.0165 | -0.0154 | 0.0115 | 0.0037 |
|  | (0.0987) | (0.0916) | (0.0896) | (0.0849) | (0.0747) | (0.0797) | (0.0762) | (0.0754) | (0.0665) | (0.0648) | (0.0608) | (0.0559) |
| N | 352,553 | 357,704 | 361,468 | 364,408 | 366,774 | 368,937 | 369,869 | 371,375 | 373,024 | 375,498 | 379,334 | 383,493 |
| Adj. R^2^ | 0.6727 | 0.6835 | 0.6922 | 0.6959 | 0.7003 | 0.7016 | 0.7022 | 0.7040 | 0.7052 | 0.7067 | 0.7098 | 0.7129 |
| Panel G: NPP (net primary productivity, kg*C/m^2^) | | | | | | | | | | | | |
| Post WF installation | -0.0037 | -0.0057 | -0.0062* | -0.0047 | -0.0040 | -0.0030 | -0.0014 | -0.0001 | 0.0003 | 0.0003 | -0.0000 | -0.0000 |
|  | (0.0037) | (0.0035) | (0.0033) | (0.0030) | (0.0027) | (0.0027) | (0.0027) | (0.0027) | (0.0026) | (0.0024) | (0.0022) | (0.0020) |
| N | 353,916 | 359,076 | 362,868 | 365,856 | 368,232 | 370,404 | 371,340 | 372,852 | 374,508 | 376,956 | 380,844 | 385,020 |
| Adj. R^2^ | 0.9620 | 0.9662 | 0.9718 | 0.9761 | 0.9801 | 0.9806 | 0.9805 | 0.9817 | 0.9826 | 0.9840 | 0.9853 | 0.9883 |
| Panel H: PTC (percentage of tree cover, %) | | | | | | | | | | | | |
| Post WF installation | -0.0257 | -0.1521 | 0.0226 | 0.0461 | -0.0176 | -0.1665 | -0.2195 | -0.1294 | -0.2066 | -0.2733 | -0.0825 | -0.0896 |
|  | (0.3962) | (0.3442) | (0.3001) | (0.2656) | (0.2447) | (0.2405) | (0.2305) | (0.2262) | (0.2098) | (0.2015) | (0.1878) | (0.1630) |
| N | 374,712 | 379,212 | 381,348 | 382,980 | 383,856 | 385,020 | 385,956 | 385,956 | 386,604 | 386,820 | 387,900 | 388,548 |
| Adj. R^2^ | 0.8940 | 0.9087 | 0.9217 | 0.9321 | 0.9420 | 0.9450 | 0.9467 | 0.9485 | 0.9509 | 0.9546 | 0.9570 | 0.9632 |
| Panel I: PNTV (percentage of non-tree vegetation, %) | | | | | | | | | | | | |
| Post WF installation | -0.9665 | -1.1507** | -0.9753* | -1.6743** | -0.8322* | -0.6515 | -0.5135 | -0.4379 | -0.4656 | -0.1749 | -0.0114 | -0.0937 |
|  | (0.6229) | (0.5615) | (0.5046) | (0.7175) | (0.4822) | (0.4678) | (0.4521) | (0.4355) | (0.3976) | (0.3752) | (0.3333) | (0.2653) |
| N | 374,712 | 379,212 | 381,348 | 362,760 | 383,856 | 385,020 | 385,956 | 385,956 | 386,604 | 386,820 | 387,900 | 388,548 |
| Adj. R^2^ | 0.7969 | 0.8379 | 0.8561 | 0.5913 | 0.8808 | 0.8879 | 0.8930 | 0.8992 | 0.9049 | 0.9091 | 0.9224 | 0.9359 |
| Panel J: PNV (percentage of non-vegetation, %) | | | | | | | | | | | | |
| Post WF installation | 0.9922 | 1.3027** | 0.9527* | 0.6121 | 0.8499* | 0.8181* | 0.7330* | 0.5674 | 0.6721* | 0.4483 | 0.0939 | 0.1834 |
|  | (0.6217) | (0.5627) | (0.5227) | (0.4834) | (0.4764) | (0.4598) | (0.4406) | (0.4274) | (0.3947) | (0.3647) | (0.3137) | (0.2549) |
| N | 374,712 | 379,212 | 381,348 | 382,980 | 383,856 | 385,020 | 385,956 | 385,956 | 386,604 | 386,820 | 387,900 | 388,548 |
| Adj. R^2^ | 0.8960 | 0.9150 | 0.9224 | 0.9306 | 0.9363 | 0.9389 | 0.9417 | 0.9452 | 0.9485 | 0.9514 | 0.9596 | 0.9663 |
|  |  |  |  |  |  |  |  |  |  |  |  |  |
| Controls | × | × | × | × | × | × | × | × | × | × | × | × |
| Year×County FE | × | × | × | × | × | × | × | × | × | × | × | × |
| Month FE | × | × | × | × | × | × | × | × | × | × | × | × |
| Terrain FE | × | × | × | × | × | × | × | × | × | × | × | × |

Note: The symbols ∗, ∗∗, and ∗∗∗ indicate significance levels at 10%, 5%, and 1%, respectively. "FE" stands for "fixed effects". Clustered standard errors at the wind farm level are shown in parentheses. Each panel depicts a single regression at the plant-year level, utilizing a buffer difference model to estimate the causal effect of wind farms on plant diversity indicators. The buffer model controls for the spatial dependence of observations, allowing for more accurate estimates of the treatment effect.

**Supplementary Table 5. Dynamic effects of wind farm on plant diversity by distance.**

|  | (1) | (2) | (3) | (4) | (5) | (6) | (7) | (8) | (9) | (10) | (11) | (12) |
| --- | --- | --- | --- | --- | --- | --- | --- | --- | --- | --- | --- | --- |
|  | 0-1 km | 1-2 km | 2-3 km | 3-4 km | 4-5 km | 5-6 km | 6-7 km | 7-8 km | 8-9 km | 9-10 km | 10-15 km | 15-20 km |
| Panel A: NDVI (normalized difference vegetation index) | | | | | | | | | | | | |
| -6 to 0 months | -0.0055*** | -0.0068*** | -0.0066*** | -0.0057*** | -0.0056*** | -0.0060*** | -0.0059*** | -0.0053*** | -0.0050*** | -0.0044** | -0.0032* | -0.0034** |
|  | (0.0021) | (0.0021) | (0.0021) | (0.0020) | (0.0020) | (0.0019) | (0.0019) | (0.0019) | (0.0018) | (0.0018) | (0.0017) | (0.0016) |
| 1st year | -0.0082*** | -0.0105*** | -0.0091*** | -0.0072*** | -0.0067*** | -0.0067*** | -0.0062*** | -0.0043** | -0.0039* | -0.0027 | -0.0016 | -0.0021 |
|  | (0.0028) | (0.0028) | (0.0027) | (0.0025) | (0.0025) | (0.0023) | (0.0023) | (0.0021) | (0.0020) | (0.0019) | (0.0016) | (0.0014) |
| 2nd year | -0.0085*** | -0.0119*** | -0.0102*** | -0.0083*** | -0.0080*** | -0.0080*** | -0.0077*** | -0.0058** | -0.0053** | -0.0037* | -0.0018 | -0.0027* |
|  | (0.0031) | (0.0032) | (0.0031) | (0.0029) | (0.0028) | (0.0026) | (0.0027) | (0.0024) | (0.0023) | (0.0022) | (0.0019) | (0.0016) |
| 3rd year | -0.0101*** | -0.0141*** | -0.0123*** | -0.0098*** | -0.0097*** | -0.0098*** | -0.0094*** | -0.0073*** | -0.0067*** | -0.0051** | -0.0024 | -0.0033* |
|  | (0.0035) | (0.0036) | (0.0035) | (0.0033) | (0.0032) | (0.0030) | (0.0030) | (0.0027) | (0.0026) | (0.0024) | (0.0021) | (0.0018) |
| > 3rd year | -0.0135*** | -0.0180*** | -0.0166*** | -0.0128*** | -0.0131*** | -0.0133*** | -0.0129*** | -0.0105*** | -0.0097*** | -0.0078** | -0.0034 | -0.0042* |
|  | (0.0043) | (0.0045) | (0.0045) | (0.0041) | (0.0040) | (0.0037) | (0.0038) | (0.0035) | (0.0033) | (0.0031) | (0.0027) | (0.0023) |
| N | 371,333 | 375,510 | 378,003 | 379,844 | 380,814 | 381,970 | 382,898 | 382,898 | 383,540 | 383,754 | 384,824 | 385,466 |
| Adj. R^2^ | 0.7575 | 0.7644 | 0.7710 | 0.7769 | 0.7803 | 0.7838 | 0.7852 | 0.7876 | 0.7900 | 0.7923 | 0.7983 | 0.8034 |
| Panel B: EVI (enhanced vegetation index) | | | | | | | | | | | | |
| -6 to 0 months | -0.0043*** | -0.0049*** | -0.0050*** | -0.0045*** | -0.0044*** | -0.0044*** | -0.0043*** | -0.0038*** | 0.0024 | -0.0031*** | -0.0023** | -0.0024** |
|  | (0.0013) | (0.0013) | (0.0013) | (0.0013) | (0.0012) | (0.0012) | (0.0012) | (0.0011) | (0.0027) | (0.0011) | (0.0010) | (0.0010) |
| 1st year | -0.0058*** | -0.0068*** | -0.0064*** | -0.0054*** | -0.0051*** | -0.0046*** | -0.0041*** | -0.0028** | 0.0018 | -0.0016 | -0.0008 | -0.0010 |
|  | (0.0019) | (0.0018) | (0.0017) | (0.0016) | (0.0016) | (0.0015) | (0.0015) | (0.0014) | (0.0050) | (0.0012) | (0.0010) | (0.0008) |
| 2nd year | -0.0068*** | -0.0084*** | -0.0077*** | -0.0066*** | -0.0063*** | -0.0057*** | -0.0053*** | -0.0040** | 0.0019 | -0.0024* | -0.0011 | -0.0014 |
|  | (0.0021) | (0.0020) | (0.0020) | (0.0019) | (0.0018) | (0.0017) | (0.0017) | (0.0015) | (0.0058) | (0.0013) | (0.0011) | (0.0009) |
| 3rd year | -0.0081*** | -0.0100*** | -0.0094*** | -0.0079*** | -0.0077*** | -0.0071*** | -0.0066*** | -0.0050*** | 0.0023 | -0.0033** | -0.0015 | -0.0018* |
|  | (0.0023) | (0.0022) | (0.0022) | (0.0021) | (0.0020) | (0.0019) | (0.0019) | (0.0017) | (0.0066) | (0.0015) | (0.0013) | (0.0010) |
| > 3rd year | -0.0106*** | -0.0126*** | -0.0125*** | -0.0103*** | -0.0103*** | -0.0095*** | -0.0090*** | -0.0071*** | 0.0032 | -0.0050*** | -0.0023 | -0.0024* |
|  | (0.0027) | (0.0028) | (0.0027) | (0.0026) | (0.0025) | (0.0023) | (0.0024) | (0.0021) | (0.0086) | (0.0019) | (0.0016) | (0.0013) |
| N | 371,333 | 375,510 | 378,003 | 379,844 | 380,814 | 381,970 | 382,898 | 382,898 | 383,248 | 383,754 | 384,824 | 385,466 |
| Adj. R^2^ | 0.7506 | 0.7586 | 0.7652 | 0.7720 | 0.7769 | 0.7807 | 0.7822 | 0.7848 | 0.5496 | 0.7895 | 0.7970 | 0.8030 |
| Panel C: FPAR (fraction of photosynthetically active radiation) | | | | | | | | | | | | |
| -6 to 0 months | -0.4567** | -0.3643* | -0.4111** | -0.3659* | -0.3454* | -0.3063* | -0.3025* | -0.3140* | -0.2886* | -0.2541 | -0.2036 | -0.2079 |
|  | (0.2188) | (0.2036) | (0.1936) | (0.1869) | (0.1840) | (0.1815) | (0.1789) | (0.1770) | (0.1746) | (0.1727) | (0.1669) | (0.1592) |
| 1st year | -0.7070*** | -0.6849*** | -0.6315*** | -0.4309** | -0.4218** | -0.2434 | -0.1905 | -0.0586 | 0.0121 | 0.0403 | -0.0637 | -0.1028 |
|  | (0.2225) | (0.2158) | (0.2114) | (0.1811) | (0.1658) | (0.1614) | (0.1661) | (0.1581) | (0.1517) | (0.1523) | (0.1264) | (0.1128) |
| 2nd year | -0.7838*** | -0.7483*** | -0.6948*** | -0.4613** | -0.4444** | -0.2613 | -0.2111 | -0.0752 | -0.0095 | 0.0365 | -0.0055 | -0.0588 |
|  | (0.2549) | (0.2465) | (0.2388) | (0.2078) | (0.1900) | (0.1855) | (0.1889) | (0.1813) | (0.1738) | (0.1730) | (0.1449) | (0.1274) |
| 3rd year | -0.9780*** | -0.9496*** | -0.8868*** | -0.6529*** | -0.6524*** | -0.4383** | -0.4005* | -0.2567 | -0.1920 | -0.1413 | -0.1380 | -0.1795 |
|  | (0.2893) | (0.2762) | (0.2638) | (0.2312) | (0.2097) | (0.2072) | (0.2092) | (0.2022) | (0.1922) | (0.1902) | (0.1623) | (0.1435) |
| > 3rd year | -1.3166*** | -1.2211*** | -1.2238*** | -0.8739*** | -0.8975*** | -0.6768** | -0.6598** | -0.5136* | -0.3848 | -0.3181 | -0.2325 | -0.2288 |
|  | (0.3844) | (0.3592) | (0.3380) | (0.2991) | (0.2730) | (0.2744) | (0.2743) | (0.2682) | (0.2526) | (0.2489) | (0.2145) | (0.1895) |
| N | 351,154 | 356,284 | 360,032 | 362,960 | 365,316 | 367,470 | 368,398 | 369,898 | 371,540 | 374,004 | 377,824 | 381,966 |
| Adj. R^2^ | 0.6907 | 0.7084 | 0.7156 | 0.7243 | 0.7269 | 0.7281 | 0.7302 | 0.7314 | 0.7340 | 0.7355 | 0.7426 | 0.7488 |
| Panel D: LAI (leaf area index, %) | | | | | | | | | | | | |
| -6 to 0 months | -0.0177* | -0.0084 | -0.0129 | -0.0105 | -0.0125 | -0.0078 | -0.0094 | -0.0100 | -0.0095 | -0.0091 | -0.0061 | -0.0081 |
|  | (0.0107) | (0.0095) | (0.0088) | (0.0081) | (0.0078) | (0.0080) | (0.0078) | (0.0077) | (0.0074) | (0.0073) | (0.0070) | (0.0066) |
| 1st year | -0.0290** | -0.0266** | -0.0253** | -0.0107 | -0.0173** | -0.0056 | -0.0047 | 0.0012 | 0.0034 | 0.0037 | -0.0006 | -0.0035 |
|  | (0.0122) | (0.0116) | (0.0118) | (0.0085) | (0.0075) | (0.0074) | (0.0077) | (0.0073) | (0.0071) | (0.0067) | (0.0054) | (0.0049) |
| 2nd year | -0.0328** | -0.0272** | -0.0263** | -0.0100 | -0.0174** | -0.0048 | -0.0039 | 0.0025 | 0.0044 | 0.0048 | 0.0030 | -0.0004 |
|  | (0.0141) | (0.0135) | (0.0131) | (0.0096) | (0.0086) | (0.0087) | (0.0088) | (0.0085) | (0.0081) | (0.0076) | (0.0063) | (0.0056) |
| 3rd year | -0.0432*** | -0.0346** | -0.0343** | -0.0188* | -0.0289*** | -0.0138 | -0.0139 | -0.0072 | -0.0049 | -0.0052 | -0.0041 | -0.0071 |
|  | (0.0162) | (0.0151) | (0.0142) | (0.0107) | (0.0096) | (0.0100) | (0.0098) | (0.0096) | (0.0090) | (0.0085) | (0.0073) | (0.0065) |
| > 3rd year | -0.0577*** | -0.0393** | -0.0476** | -0.0230 | -0.0343*** | -0.0183 | -0.0204 | -0.0144 | -0.0098 | -0.0109 | -0.0073 | -0.0087 |
|  | (0.0218) | (0.0192) | (0.0186) | (0.0140) | (0.0128) | (0.0137) | (0.0131) | (0.0129) | (0.0120) | (0.0114) | (0.0097) | (0.0088) |
| N | 351,154 | 356,284 | 360,032 | 362,960 | 365,316 | 367,470 | 368,398 | 369,898 | 371,540 | 374,004 | 377,824 | 381,966 |
| Adj. R^2^ | 0.6562 | 0.6950 | 0.6992 | 0.7204 | 0.7192 | 0.7170 | 0.7238 | 0.7215 | 0.7260 | 0.7253 | 0.7359 | 0.7421 |
| Panel E: GPP (gross primary product, g*C/m^2^) | | | | | | | | | | | | |
| -6 to 0 months | -0.2175* | -0.2338* | -0.2639** | -0.2628** | -0.2495** | -0.2861** | -0.2689** | -0.2773** | -0.2598** | -0.2327** | -0.2155** | -0.2238** |
|  | (0.1307) | (0.1250) | (0.1209) | (0.1172) | (0.1146) | (0.1120) | (0.1106) | (0.1095) | (0.1085) | (0.1076) | (0.1046) | (0.1013) |
| 1st year | -0.1850 | -0.2626** | -0.2550** | -0.2170** | -0.1909** | -0.1850* | -0.1382 | -0.0939 | -0.0537 | -0.0395 | -0.0325 | -0.0601 |
|  | (0.1220) | (0.1164) | (0.1139) | (0.1030) | (0.0923) | (0.0956) | (0.0951) | (0.0914) | (0.0834) | (0.0823) | (0.0772) | (0.0695) |
| 2nd year | -0.1278 | -0.1991 | -0.1805 | -0.1324 | -0.1074 | -0.1084 | -0.0554 | -0.0036 | 0.0336 | 0.0656 | 0.0912 | 0.0629 |
|  | (0.1391) | (0.1316) | (0.1286) | (0.1177) | (0.1059) | (0.1085) | (0.1073) | (0.1028) | (0.0933) | (0.0919) | (0.0849) | (0.0762) |
| 3rd year | -0.2715* | -0.3796** | -0.3582** | -0.3076** | -0.2807** | -0.2860** | -0.2354** | -0.1711 | -0.1366 | -0.0989 | -0.0506 | -0.0639 |
|  | (0.1580) | (0.1478) | (0.1433) | (0.1315) | (0.1181) | (0.1222) | (0.1197) | (0.1150) | (0.1045) | (0.1024) | (0.0940) | (0.0849) |
| > 3rd year | -0.4111* | -0.5621*** | -0.5518*** | -0.4652*** | -0.4339*** | -0.4753*** | -0.4128*** | -0.3323** | -0.2672** | -0.2166* | -0.1230 | -0.1082 |
|  | (0.2097) | (0.1952) | (0.1829) | (0.1690) | (0.1531) | (0.1574) | (0.1530) | (0.1477) | (0.1339) | (0.1313) | (0.1187) | (0.1084) |
| N | 352,553 | 357,704 | 361,468 | 364,408 | 366,774 | 368,937 | 369,869 | 371,375 | 373,024 | 375,498 | 379,334 | 383,493 |
| Adj. R^2^ | 0.7314 | 0.7408 | 0.7490 | 0.7535 | 0.7583 | 0.7603 | 0.7613 | 0.7631 | 0.7649 | 0.7659 | 0.7702 | 0.7744 |
| Panel F: NP (net photosynthesis, g*C/m^2^) | | | | | | | | | | | | |
| -6 to 0 months | -0.1023 | -0.1134 | -0.1234 | -0.1271 | -0.1159 | -0.1398* | -0.1308 | -0.1443* | -0.1390* | -0.1313* | -0.1288* | -0.1345* |
|  | (0.0928) | (0.0890) | (0.0863) | (0.0848) | (0.0832) | (0.0818) | (0.0809) | (0.0798) | (0.0794) | (0.0790) | (0.0771) | (0.0752) |
| 1st year | -0.0687 | -0.1120 | -0.1236 | -0.0996 | -0.0889 | -0.0978 | -0.0637 | -0.0385 | -0.0186 | -0.0167 | -0.0134 | -0.0325 |
|  | (0.0821) | (0.0768) | (0.0762) | (0.0699) | (0.0636) | (0.0675) | (0.0655) | (0.0640) | (0.0578) | (0.0569) | (0.0552) | (0.0503) |
| 2nd year | 0.0026 | -0.0422 | -0.0526 | -0.0197 | -0.0115 | -0.0276 | 0.0093 | 0.0405 | 0.0588 | 0.0737 | 0.0854 | 0.0601 |
|  | (0.0923) | (0.0859) | (0.0843) | (0.0785) | (0.0717) | (0.0754) | (0.0727) | (0.0702) | (0.0630) | (0.0617) | (0.0591) | (0.0537) |
| 3rd year | -0.0825 | -0.1561 | -0.1588* | -0.1199 | -0.1070 | -0.1276 | -0.0873 | -0.0480 | -0.0361 | -0.0222 | 0.0007 | -0.0190 |
|  | (0.1044) | (0.0968) | (0.0945) | (0.0887) | (0.0808) | (0.0857) | (0.0818) | (0.0789) | (0.0712) | (0.0697) | (0.0656) | (0.0594) |
| > 3rd year | -0.2009 | -0.2842** | -0.2925** | -0.2368** | -0.2258** | -0.2729** | -0.2199** | -0.1723* | -0.1423 | -0.1200 | -0.0641 | -0.0655 |
|  | (0.1371) | (0.1265) | (0.1193) | (0.1128) | (0.1038) | (0.1094) | (0.1041) | (0.1001) | (0.0901) | (0.0886) | (0.0813) | (0.0739) |
| N | 352,553 | 357,704 | 361,468 | 364,408 | 366,774 | 368,937 | 369,869 | 371,375 | 373,024 | 375,498 | 379,334 | 383,493 |
| Adj. R^2^ | 0.6727 | 0.6835 | 0.6922 | 0.6959 | 0.7003 | 0.7016 | 0.7022 | 0.7040 | 0.7052 | 0.7067 | 0.7098 | 0.7129 |
| Panel G: NPP (net primary productivity, kg*C/m^2^) | | | | | | | | | | | | |
| -6 to 0 months | -0.0007 | -0.0012 | -0.0018 | -0.0013 | -0.0017 | -0.0020 | -0.0015 | -0.0010 | -0.0010 | -0.0006 | 0.0002 | 0.0006 |
|  | (0.0017) | (0.0016) | (0.0015) | (0.0014) | (0.0013) | (0.0012) | (0.0012) | (0.0012) | (0.0011) | (0.0011) | (0.0010) | (0.0009) |
| 1st year | -0.0026 | -0.0044 | -0.0052* | -0.0037 | -0.0034 | -0.0025 | -0.0012 | -0.0000 | 0.0002 | 0.0005 | -0.0001 | -0.0003 |
|  | (0.0032) | (0.0031) | (0.0029) | (0.0026) | (0.0024) | (0.0023) | (0.0024) | (0.0023) | (0.0022) | (0.0021) | (0.0019) | (0.0018) |
| 2nd year | -0.0033 | -0.0050 | -0.0057* | -0.0042 | -0.0036 | -0.0030 | -0.0015 | -0.0003 | -0.0001 | 0.0003 | 0.0000 | -0.0003 |
|  | (0.0036) | (0.0034) | (0.0033) | (0.0030) | (0.0027) | (0.0026) | (0.0027) | (0.0026) | (0.0024) | (0.0023) | (0.0021) | (0.0020) |
| 3rd year | -0.0030 | -0.0058 | -0.0066* | -0.0048 | -0.0043 | -0.0037 | -0.0021 | -0.0007 | -0.0008 | -0.0004 | -0.0003 | -0.0005 |
|  | (0.0041) | (0.0039) | (0.0037) | (0.0034) | (0.0031) | (0.0030) | (0.0030) | (0.0029) | (0.0027) | (0.0026) | (0.0024) | (0.0022) |
| > 3rd year | -0.0056 | -0.0082 | -0.0088* | -0.0065 | -0.0064 | -0.0061 | -0.0045 | -0.0030 | -0.0027 | -0.0018 | -0.0009 | -0.0003 |
|  | (0.0054) | (0.0051) | (0.0046) | (0.0043) | (0.0039) | (0.0039) | (0.0039) | (0.0038) | (0.0035) | (0.0034) | (0.0031) | (0.0028) |
| N | 353,916 | 359,076 | 362,868 | 365,856 | 368,232 | 370,404 | 371,340 | 372,852 | 374,508 | 376,956 | 380,844 | 385,020 |
| Adj. R^2^ | 0.9620 | 0.9662 | 0.9718 | 0.9761 | 0.9801 | 0.9806 | 0.9805 | 0.9817 | 0.9826 | 0.9840 | 0.9853 | 0.9883 |
| Panel H: PTC (percentage of tree cover, %) | | | | | | | | | | | | |
| -6 to 0 months | -0.0277 | -0.0043 | 0.0405 | 0.0869 | 0.0490 | -0.0697 | -0.1157 | -0.1029 | -0.1253 | -0.1131 | 0.0006 | -0.0027 |
|  | (0.2050) | (0.1779) | (0.1521) | (0.1367) | (0.1291) | (0.1293) | (0.1239) | (0.1217) | (0.1151) | (0.1126) | (0.1017) | (0.0845) |
| 1st year | -0.1300 | -0.2223 | -0.0392 | 0.0076 | -0.0148 | -0.1485 | -0.1587 | -0.0841 | -0.1266 | -0.1866 | -0.0926 | -0.0737 |
|  | (0.3368) | (0.2967) | (0.2585) | (0.2281) | (0.2060) | (0.2043) | (0.1946) | (0.1892) | (0.1758) | (0.1681) | (0.1568) | (0.1367) |
| 2nd year | -0.0069 | -0.1248 | 0.0494 | 0.1115 | 0.0358 | -0.1585 | -0.1700 | -0.1095 | -0.1785 | -0.2433 | -0.0745 | -0.0685 |
|  | (0.3907) | (0.3418) | (0.2969) | (0.2610) | (0.2380) | (0.2367) | (0.2255) | (0.2214) | (0.2063) | (0.1975) | (0.1834) | (0.1587) |
| 3rd year | 0.0733 | -0.0817 | 0.0953 | 0.1275 | 0.0375 | -0.1868 | -0.2372 | -0.2046 | -0.2921 | -0.3407 | -0.0882 | -0.1008 |
|  | (0.4566) | (0.3947) | (0.3421) | (0.2999) | (0.2760) | (0.2742) | (0.2611) | (0.2564) | (0.2398) | (0.2306) | (0.2135) | (0.1836) |
| > 3rd year | 0.0743 | -0.1380 | 0.1118 | 0.2275 | 0.1144 | -0.2135 | -0.3404 | -0.3096 | -0.4190 | -0.4730 | -0.1071 | -0.1405 |
|  | (0.6112) | (0.5233) | (0.4537) | (0.4020) | (0.3787) | (0.3771) | (0.3632) | (0.3574) | (0.3339) | (0.3247) | (0.2999) | (0.2552) |
| N | 374,712 | 379,212 | 381,348 | 382,980 | 383,856 | 385,020 | 385,956 | 385,956 | 386,604 | 386,820 | 387,900 | 388,548 |
| Adj. R^2^ | 0.8940 | 0.9087 | 0.9217 | 0.9321 | 0.9420 | 0.9450 | 0.9467 | 0.9486 | 0.9509 | 0.9547 | 0.9570 | 0.9632 |
| Panel I: PNTV (percentage of non-tree vegetation, %) | | | | | | | | | | | | |
| -6 to 0 months | -0.2069 | -0.3975 | -0.2334 | -0.7900** | -0.2523 | -0.1188 | -0.0657 | -0.1053 | -0.0752 | -0.0203 | -0.0088 | -0.0036 |
|  | (0.2757) | (0.2506) | (0.2400) | (0.3067) | (0.2109) | (0.2024) | (0.1952) | (0.1849) | (0.1757) | (0.1694) | (0.1459) | (0.1215) |
| 1st year | -0.7522 | -1.0193* | -0.7492 | -1.3798* | -0.6977 | -0.5911 | -0.4453 | -0.3917 | -0.3906 | -0.1533 | -0.0043 | -0.1039 |
|  | (0.6296) | (0.5527) | (0.4879) | (0.7261) | (0.4645) | (0.4507) | (0.4342) | (0.4177) | (0.3756) | (0.3511) | (0.3070) | (0.2365) |
| 2nd year | -0.9951 | -1.2373** | -1.0222* | -1.6920** | -0.9052* | -0.7224 | -0.5962 | -0.5030 | -0.4932 | -0.2020 | -0.0554 | -0.1609 |
|  | (0.6590) | (0.5850) | (0.5284) | (0.7513) | (0.4981) | (0.4868) | (0.4683) | (0.4501) | (0.4106) | (0.3855) | (0.3382) | (0.2649) |
| 3rd year | -1.1439* | -1.3751** | -1.1398** | -1.7889** | -1.0567* | -0.7389 | -0.5689 | -0.4839 | -0.4702 | -0.1617 | -0.0271 | -0.1391 |
|  | (0.6950) | (0.6308) | (0.5752) | (0.7942) | (0.5389) | (0.5248) | (0.5071) | (0.4861) | (0.4463) | (0.4209) | (0.3727) | (0.2973) |
| > 3rd year | -1.3000 | -1.5144** | -1.2588* | -2.5707*** | -1.1924* | -0.7905 | -0.6071 | -0.5547 | -0.5369 | -0.1579 | 0.0236 | -0.0418 |
|  | (0.7964) | (0.7301) | (0.6725) | (0.8933) | (0.6290) | (0.6116) | (0.5952) | (0.5721) | (0.5349) | (0.5131) | (0.4606) | (0.3783) |
| N | 374,712 | 379,212 | 381,348 | 362,760 | 383,856 | 385,020 | 385,956 | 385,956 | 386,604 | 386,820 | 387,900 | 388,548 |
| Adj. R^2^ | 0.7969 | 0.8379 | 0.8561 | 0.5915 | 0.8809 | 0.8879 | 0.8930 | 0.8992 | 0.9049 | 0.9091 | 0.9224 | 0.9359 |
| Panel J: PNV (percentage of non-vegetation, %) | | | | | | | | | | | | |
| -6 to 0 months | 0.2347 | 0.4019 | 0.1929 | 0.1518 | 0.2033 | 0.1885 | 0.1814 | 0.2082 | 0.2005 | 0.1334 | 0.0082 | 0.0063 |
|  | (0.2662) | (0.2473) | (0.2433) | (0.2233) | (0.2079) | (0.1983) | (0.1905) | (0.1855) | (0.1787) | (0.1711) | (0.1454) | (0.1246) |
| 1st year | 0.8822 | 1.2416** | 0.7884 | 0.5657 | 0.7125 | 0.7396* | 0.6040 | 0.4757 | 0.5171 | 0.3400 | 0.0969 | 0.1775 |
|  | (0.6311) | (0.5552) | (0.5053) | (0.4696) | (0.4658) | (0.4489) | (0.4285) | (0.4121) | (0.3763) | (0.3452) | (0.2914) | (0.2290) |
| 2nd year | 1.0020 | 1.3621** | 0.9728* | 0.6719 | 0.8694* | 0.8808* | 0.7662* | 0.6125 | 0.6717 | 0.4454 | 0.1299 | 0.2295 |
|  | (0.6606) | (0.5893) | (0.5480) | (0.5087) | (0.4988) | (0.4828) | (0.4622) | (0.4457) | (0.4122) | (0.3804) | (0.3236) | (0.2589) |
| 3rd year | 1.0706 | 1.4568** | 1.0445* | 0.7152 | 1.0192* | 0.9257* | 0.8061 | 0.6884 | 0.7623* | 0.5024 | 0.1152 | 0.2399 |
|  | (0.6914) | (0.6315) | (0.5925) | (0.5480) | (0.5337) | (0.5154) | (0.4954) | (0.4777) | (0.4440) | (0.4112) | (0.3538) | (0.2888) |
| > 3rd year | 1.2257 | 1.6524** | 1.1470* | 0.6886 | 1.0780* | 1.0040* | 0.9475* | 0.8642 | 0.9559* | 0.6309 | 0.0836 | 0.1823 |
|  | (0.7799) | (0.7227) | (0.6898) | (0.6359) | (0.6118) | (0.5882) | (0.5675) | (0.5510) | (0.5237) | (0.4923) | (0.4296) | (0.3638) |
| N | 374,712 | 379,212 | 381,348 | 382,980 | 383,856 | 385,020 | 385,956 | 385,956 | 386,604 | 386,820 | 387,900 | 388,548 |
| Adj. R^2^ | 0.8960 | 0.9150 | 0.9224 | 0.9306 | 0.9363 | 0.9389 | 0.9417 | 0.9452 | 0.9486 | 0.9514 | 0.9596 | 0.9663 |
|  |  |  |  |  |  |  |  |  |  |  |  |  |
| Controls | × | × | × | × | × | × | × | × | × | × | × | × |
| Year×County FE | × | × | × | × | × | × | × | × | × | × | × | × |
| Month FE | × | × | × | × | × | × | × | × | × | × | × | × |
| Terrain FE | × | × | × | × | × | × | × | × | × | × | × | × |

Note: The symbols ∗, ∗∗, and ∗∗∗ indicate significance levels at 10%, 5%, and 1%, respectively. "FE" stands for "fixed effects". Clustered standard errors at the wind farm level are shown in parentheses. Each panel depicts a single regression at the plant-year level, utilizing a buffer difference model to estimate the causal effect of wind farms on plant diversity indicators. The buffer model controls for the spatial dependence of observations, allowing for more accurate estimates of the treatment effect.

**Supplementary Table 6. Seasonal heterogeneous effects of wind farm on plant diversity by distance.**

|  | (1) | (2) | (3) | (4) | (5) | (6) | (7) | (8) | (9) | (10) | (11) | (12) |
| --- | --- | --- | --- | --- | --- | --- | --- | --- | --- | --- | --- | --- |
|  | 0-1 km | 1-2 km | 2-3 km | 3-4 km | 4-5 km | 5-6 km | 6-7 km | 7-8 km | 8-9 km | 9-10 km | 10-15 km | 15-20 km |
| Panel A: NDVI (normalized difference vegetation index) | | | | | | | | | | | | |
| Mar to May | -0.0070** | -0.0098*** | -0.0086** | -0.0065** | -0.0069** | -0.0069** | -0.0069** | -0.0048* | -0.0045* | -0.0035 | -0.0009 | -0.0024 |
|  | (0.0033) | (0.0034) | (0.0034) | (0.0031) | (0.0030) | (0.0028) | (0.0029) | (0.0026) | (0.0024) | (0.0023) | (0.0019) | (0.0017) |
| N | 93,572 | 94,624 | 95,253 | 95,716 | 95,964 | 96,255 | 96,489 | 96,489 | 96,651 | 96,705 | 96,975 | 97,137 |
| Adj. R^2^ | 0.8144 | 0.8227 | 0.8276 | 0.8356 | 0.8400 | 0.8447 | 0.8469 | 0.8504 | 0.8532 | 0.8564 | 0.8648 | 0.8699 |
| Jun to Aug | -0.0147** | -0.0188*** | -0.0178*** | -0.0142*** | -0.0145*** | -0.0129*** | -0.0115** | -0.0088* | -0.0090** | -0.0081* | -0.0045 | -0.0039 |
|  | (0.0061) | (0.0058) | (0.0056) | (0.0054) | (0.0053) | (0.0050) | (0.0049) | (0.0046) | (0.0044) | (0.0043) | (0.0039) | (0.0033) |
| N | 93,582 | 94,629 | 95,270 | 95,728 | 95,963 | 96,255 | 96,489 | 96,489 | 96,651 | 96,705 | 96,975 | 97,137 |
| Adj. R^2^ | 0.8142 | 0.8216 | 0.8305 | 0.8362 | 0.8407 | 0.8473 | 0.8484 | 0.8520 | 0.8564 | 0.8594 | 0.8682 | 0.8764 |
| Sep to Nov | -0.0086** | -0.0135*** | -0.0124*** | -0.0093*** | -0.0096*** | -0.0100*** | -0.0096*** | -0.0066** | -0.0065** | -0.0052** | -0.0028 | -0.0036* |
|  | (0.0035) | (0.0038) | (0.0037) | (0.0034) | (0.0032) | (0.0031) | (0.0031) | (0.0029) | (0.0026) | (0.0026) | (0.0022) | (0.0019) |
| N | 93,547 | 94,623 | 95,247 | 95,711 | 95,964 | 96,255 | 96,489 | 96,489 | 96,651 | 96,705 | 96,975 | 97,137 |
| Adj. R^2^ | 0.8264 | 0.8319 | 0.8391 | 0.8472 | 0.8516 | 0.8568 | 0.8588 | 0.8620 | 0.8657 | 0.8686 | 0.8750 | 0.8809 |
| Dec to Feb | -0.0105*** | -0.0119*** | -0.0105*** | -0.0081*** | -0.0072*** | -0.0072*** | -0.0073*** | -0.0051** | -0.0050** | -0.0037* | -0.0010 | -0.0018 |
|  | (0.0029) | (0.0027) | (0.0027) | (0.0024) | (0.0023) | (0.0023) | (0.0025) | (0.0022) | (0.0021) | (0.0019) | (0.0017) | (0.0016) |
| N | 90,600 | 91,633 | 92,233 | 92,689 | 92,923 | 93,205 | 93,431 | 93,431 | 93,587 | 93,639 | 93,899 | 94,055 |
| Adj. R^2^ | 0.8365 | 0.8444 | 0.8509 | 0.8596 | 0.8628 | 0.8661 | 0.8679 | 0.8703 | 0.8725 | 0.8755 | 0.8801 | 0.8846 |
| Panel B: EVI (enhanced vegetation index) | | | | | | | | | | | | |
| Mar to May | -0.0059*** | -0.0073*** | -0.0070*** | -0.0056*** | -0.0057*** | -0.0051*** | -0.0048*** | -0.0033** | 0.0041 | -0.0022 | -0.0005 | -0.0012 |
|  | (0.0021) | (0.0021) | (0.0022) | (0.0020) | (0.0019) | (0.0018) | (0.0018) | (0.0017) | (0.0070) | (0.0014) | (0.0012) | (0.0010) |
| N | 93,572 | 94,624 | 95,253 | 95,716 | 95,964 | 96,255 | 96,489 | 96,489 | 96,578 | 96,705 | 96,975 | 97,137 |
| Adj. R^2^ | 0.7618 | 0.7722 | 0.7782 | 0.7887 | 0.7952 | 0.8005 | 0.8029 | 0.8067 | 0.3400 | 0.8139 | 0.8258 | 0.8333 |
| Jun to Aug | -0.0128*** | -0.0151*** | -0.0154*** | -0.0130*** | -0.0127*** | -0.0105*** | -0.0093*** | -0.0071** | -0.0080 | -0.0061** | -0.0031 | -0.0023 |
|  | (0.0044) | (0.0041) | (0.0039) | (0.0039) | (0.0037) | (0.0035) | (0.0034) | (0.0032) | (0.0097) | (0.0030) | (0.0026) | (0.0023) |
| N | 93,582 | 94,629 | 95,270 | 95,728 | 95,963 | 96,255 | 96,489 | 96,489 | 96,578 | 96,705 | 96,975 | 97,137 |
| Adj. R^2^ | 0.7747 | 0.7854 | 0.7963 | 0.8031 | 0.8089 | 0.8163 | 0.8181 | 0.8211 | 0.3140 | 0.8275 | 0.8368 | 0.8452 |
| Sep to Nov | -0.0067*** | -0.0087*** | -0.0083*** | -0.0064*** | -0.0065*** | -0.0062*** | -0.0057*** | -0.0037** | 0.0028 | -0.0029** | -0.0014 | -0.0016 |
|  | (0.0021) | (0.0022) | (0.0021) | (0.0020) | (0.0019) | (0.0018) | (0.0019) | (0.0017) | (0.0071) | (0.0014) | (0.0011) | (0.0010) |
| N | 93,547 | 94,623 | 95,247 | 95,711 | 95,964 | 96,255 | 96,489 | 96,489 | 96,574 | 96,705 | 96,975 | 97,137 |
| Adj. R^2^ | 0.8317 | 0.8384 | 0.8461 | 0.8563 | 0.8622 | 0.8683 | 0.8701 | 0.8734 | 0.3806 | 0.8801 | 0.8889 | 0.8956 |
| Dec to Feb | -0.0064*** | -0.0064*** | -0.0059*** | -0.0047*** | -0.0043*** | -0.0039*** | -0.0039*** | -0.0025* | 0.0070 | -0.0017 | -0.0003 | -0.0005 |
|  | (0.0016) | (0.0015) | (0.0014) | (0.0013) | (0.0013) | (0.0013) | (0.0015) | (0.0013) | (0.0053) | (0.0010) | (0.0009) | (0.0009) |
| N | 90,600 | 91,633 | 92,233 | 92,689 | 92,923 | 93,205 | 93,431 | 93,431 | 93,518 | 93,639 | 93,899 | 94,055 |
| Adj. R^2^ | 0.8071 | 0.8188 | 0.8275 | 0.8393 | 0.8440 | 0.8476 | 0.8486 | 0.8515 | 0.2610 | 0.8592 | 0.8658 | 0.8709 |
| Panel C: FPAR (fraction of photosynthetically active radiation) | | | | | | | | | | | | |
| Mar to May | -0.7939** | -0.6133** | -0.6649** | -0.4457* | -0.5031** | -0.2774 | -0.2988 | -0.1799 | -0.0584 | -0.0115 | -0.0396 | -0.1118 |
|  | (0.3231) | (0.3050) | (0.3042) | (0.2527) | (0.2330) | (0.2231) | (0.2212) | (0.2124) | (0.2057) | (0.2014) | (0.1645) | (0.1451) |
| N | 88,488 | 89,781 | 90,726 | 91,464 | 92,058 | 92,601 | 92,835 | 93,213 | 93,627 | 94,248 | 95,211 | 96,255 |
| Adj. R^2^ | 0.7627 | 0.7889 | 0.7941 | 0.8086 | 0.8137 | 0.8161 | 0.8203 | 0.8218 | 0.8252 | 0.8288 | 0.8402 | 0.8488 |
| Jun to Aug | -1.1150** | -1.3290*** | -1.4107*** | -0.9896** | -0.9429** | -0.6630 | -0.5086 | -0.3678 | -0.2021 | -0.1272 | -0.2077 | -0.2071 |
|  | (0.4843) | (0.4686) | (0.4510) | (0.4317) | (0.4055) | (0.4032) | (0.3997) | (0.3935) | (0.3765) | (0.3801) | (0.3400) | (0.3204) |
| N | 88,488 | 89,781 | 90,726 | 91,464 | 92,058 | 92,601 | 92,835 | 93,213 | 93,627 | 94,248 | 95,211 | 96,255 |
| Adj. R^2^ | 0.7257 | 0.7458 | 0.7515 | 0.7598 | 0.7660 | 0.7693 | 0.7682 | 0.7712 | 0.7747 | 0.7775 | 0.7907 | 0.7978 |
| Sep to Nov | -1.0528*** | -1.0681*** | -0.9881*** | -0.7190*** | -0.7029*** | -0.5329** | -0.5419** | -0.3265 | -0.2280 | -0.1577 | -0.2604 | -0.2960* |
|  | (0.3202) | (0.3110) | (0.2908) | (0.2587) | (0.2336) | (0.2328) | (0.2264) | (0.2217) | (0.2136) | (0.2134) | (0.1853) | (0.1748) |
| N | 88,488 | 89,781 | 90,726 | 91,464 | 92,058 | 92,601 | 92,835 | 93,213 | 93,627 | 94,248 | 95,211 | 96,255 |
| Adj. R^2^ | 0.7652 | 0.7840 | 0.7920 | 0.8014 | 0.8058 | 0.8087 | 0.8108 | 0.8128 | 0.8174 | 0.8203 | 0.8284 | 0.8349 |
| Dec to Feb | -0.9966*** | -0.6923*** | -0.5819** | -0.3429* | -0.2950* | -0.1589 | -0.2438 | -0.1121 | -0.0539 | -0.0011 | 0.0358 | 0.0531 |
|  | (0.2940) | (0.2604) | (0.2433) | (0.1860) | (0.1748) | (0.1761) | (0.1901) | (0.1809) | (0.1745) | (0.1699) | (0.1397) | (0.1314) |
| N | 85,690 | 86,941 | 87,854 | 88,568 | 89,142 | 89,667 | 89,893 | 90,259 | 90,659 | 91,260 | 92,191 | 93,201 |
| Adj. R^2^ | 0.8134 | 0.8386 | 0.8457 | 0.8604 | 0.8628 | 0.8613 | 0.8641 | 0.8614 | 0.8651 | 0.8662 | 0.8711 | 0.8755 |
| Panel D: LAI (leaf area index, %) | | | | | | | | | | | | |
| Mar to May | -0.0433** | -0.0258 | -0.0292* | -0.0102 | -0.0194* | -0.0049 | -0.0079 | -0.0022 | 0.0037 | 0.0058 | 0.0013 | -0.0050 |
|  | (0.0191) | (0.0175) | (0.0170) | (0.0115) | (0.0101) | (0.0101) | (0.0100) | (0.0097) | (0.0094) | (0.0092) | (0.0071) | (0.0064) |
| N | 88,488 | 89,781 | 90,726 | 91,464 | 92,058 | 92,601 | 92,835 | 93,213 | 93,627 | 94,248 | 95,211 | 96,255 |
| Adj. R^2^ | 0.7071 | 0.7667 | 0.7704 | 0.8082 | 0.8138 | 0.8124 | 0.8230 | 0.8202 | 0.8257 | 0.8261 | 0.8450 | 0.8549 |
| Jun to Aug | -0.0503* | -0.0566** | -0.0643** | -0.0417* | -0.0483** | -0.0305 | -0.0318 | -0.0225 | -0.0122 | -0.0097 | -0.0093 | -0.0054 |
|  | (0.0279) | (0.0257) | (0.0249) | (0.0215) | (0.0201) | (0.0201) | (0.0199) | (0.0194) | (0.0186) | (0.0184) | (0.0166) | (0.0150) |
| N | 88,488 | 89,781 | 90,726 | 91,464 | 92,058 | 92,601 | 92,835 | 93,213 | 93,627 | 94,248 | 95,211 | 96,255 |
| Adj. R^2^ | 0.7161 | 0.7519 | 0.7560 | 0.7752 | 0.7793 | 0.7787 | 0.7804 | 0.7837 | 0.7889 | 0.7896 | 0.8033 | 0.8122 |
| Sep to Nov | -0.0420*** | -0.0312** | -0.0297** | -0.0107 | -0.0214** | -0.0089 | -0.0122 | -0.0023 | 0.0011 | 0.0021 | -0.0031 | -0.0071 |
|  | (0.0154) | (0.0144) | (0.0138) | (0.0103) | (0.0095) | (0.0099) | (0.0096) | (0.0093) | (0.0088) | (0.0087) | (0.0074) | (0.0072) |
| N | 88,488 | 89,781 | 90,726 | 91,464 | 92,058 | 92,601 | 92,835 | 93,213 | 93,627 | 94,248 | 95,211 | 96,255 |
| Adj. R^2^ | 0.7715 | 0.8193 | 0.8258 | 0.8493 | 0.8546 | 0.8544 | 0.8622 | 0.8603 | 0.8673 | 0.8672 | 0.8802 | 0.8890 |
| Dec to Feb | -0.0370** | -0.0171 | -0.0171 | 0.0008 | -0.0062 | 0.0038 | 0.0013 | 0.0056 | 0.0072 | 0.0098 | 0.0066 | 0.0036 |
|  | (0.0145) | (0.0134) | (0.0126) | (0.0079) | (0.0069) | (0.0072) | (0.0076) | (0.0076) | (0.0070) | (0.0071) | (0.0051) | (0.0046) |
| N | 85,690 | 86,941 | 87,854 | 88,568 | 89,142 | 89,667 | 89,893 | 90,259 | 90,659 | 91,260 | 92,191 | 93,201 |
| Adj. R^2^ | 0.7322 | 0.7954 | 0.7883 | 0.8214 | 0.8226 | 0.8106 | 0.8203 | 0.8102 | 0.8171 | 0.8104 | 0.8214 | 0.8248 |
| Panel E: GPP (gross primary product, g*C/m^2^) | | | | | | | | | | | | |
| Mar to May | -0.2421 | -0.2614 | -0.3125 | -0.2703 | -0.2327 | -0.2321 | -0.2268 | -0.1127 | -0.0624 | -0.0417 | 0.0142 | -0.0297 |
|  | (0.2040) | (0.1908) | (0.1904) | (0.1654) | (0.1492) | (0.1496) | (0.1424) | (0.1359) | (0.1199) | (0.1142) | (0.0976) | (0.0861) |
| N | 88,488 | 89,781 | 90,726 | 91,464 | 92,058 | 92,601 | 92,835 | 93,213 | 93,627 | 94,248 | 95,211 | 96,255 |
| Adj. R^2^ | 0.7907 | 0.8038 | 0.8132 | 0.8209 | 0.8282 | 0.8313 | 0.8339 | 0.8365 | 0.8392 | 0.8425 | 0.8498 | 0.8561 |
| Jun to Aug | -0.2775 | -0.5395 | -0.6631** | -0.5539* | -0.4261 | -0.4266 | -0.3083 | -0.1422 | -0.0804 | -0.0666 | -0.0334 | 0.0142 |
|  | (0.3562) | (0.3346) | (0.3213) | (0.3116) | (0.2877) | (0.2909) | (0.2895) | (0.2890) | (0.2716) | (0.2690) | (0.2553) | (0.2345) |
| N | 88,488 | 89,781 | 90,726 | 91,464 | 92,058 | 92,601 | 92,835 | 93,213 | 93,627 | 94,248 | 95,211 | 96,255 |
| Adj. R^2^ | 0.7195 | 0.7375 | 0.7465 | 0.7514 | 0.7593 | 0.7620 | 0.7617 | 0.7655 | 0.7688 | 0.7726 | 0.7833 | 0.7945 |
| Sep to Nov | -0.3981** | -0.4687*** | -0.4437*** | -0.3843*** | -0.2886** | -0.3264*** | -0.3107*** | -0.1753 | -0.1641* | -0.1404 | -0.1028 | -0.0968 |
|  | (0.1579) | (0.1505) | (0.1427) | (0.1321) | (0.1140) | (0.1203) | (0.1127) | (0.1116) | (0.0987) | (0.0985) | (0.0883) | (0.0877) |
| N | 88,488 | 89,781 | 90,726 | 91,464 | 92,058 | 92,601 | 92,835 | 93,213 | 93,627 | 94,248 | 95,211 | 96,255 |
| Adj. R^2^ | 0.8385 | 0.8461 | 0.8536 | 0.8590 | 0.8640 | 0.8664 | 0.8674 | 0.8698 | 0.8726 | 0.8741 | 0.8788 | 0.8836 |
| Dec to Feb | -0.1961** | -0.1529* | -0.0956 | -0.0640 | -0.0432 | -0.0493 | -0.0734 | -0.0254 | -0.0331 | -0.0332 | 0.0202 | 0.0196 |
|  | (0.0945) | (0.0824) | (0.0749) | (0.0571) | (0.0524) | (0.0526) | (0.0689) | (0.0650) | (0.0576) | (0.0478) | (0.0418) | (0.0426) |
| N | 87,089 | 88,361 | 89,290 | 90,016 | 90,600 | 91,134 | 91,364 | 91,736 | 92,143 | 92,754 | 93,701 | 94,728 |
| Adj. R^2^ | 0.8634 | 0.8747 | 0.8880 | 0.8976 | 0.9009 | 0.9024 | 0.9021 | 0.9020 | 0.9048 | 0.9067 | 0.9084 | 0.9129 |
| Panel F: NP (net photosynthesis, g*C/m^2^) | | | | | | | | | | | | |
| Mar to May | -0.1439 | -0.1665 | -0.2351* | -0.2092* | -0.1769 | -0.1856 | -0.1655 | -0.0773 | -0.0365 | -0.0312 | 0.0183 | -0.0106 |
|  | (0.1465) | (0.1368) | (0.1399) | (0.1256) | (0.1135) | (0.1168) | (0.1069) | (0.1005) | (0.0884) | (0.0854) | (0.0747) | (0.0650) |
| N | 88,488 | 89,781 | 90,726 | 91,464 | 92,058 | 92,601 | 92,835 | 93,213 | 93,627 | 94,248 | 95,211 | 96,255 |
| Adj. R^2^ | 0.7801 | 0.7927 | 0.8008 | 0.8065 | 0.8134 | 0.8163 | 0.8187 | 0.8216 | 0.8240 | 0.8273 | 0.8337 | 0.8393 |
| Jun to Aug | 0.1172 | 0.0043 | -0.1154 | -0.0676 | -0.0220 | -0.0665 | 0.0316 | 0.0867 | 0.0942 | 0.0789 | 0.0510 | 0.0540 |
|  | (0.2294) | (0.2120) | (0.2054) | (0.1990) | (0.1891) | (0.1904) | (0.1888) | (0.1909) | (0.1790) | (0.1735) | (0.1658) | (0.1537) |
| N | 88,488 | 89,781 | 90,726 | 91,464 | 92,058 | 92,601 | 92,835 | 93,213 | 93,627 | 94,248 | 95,211 | 96,255 |
| Adj. R^2^ | 0.7124 | 0.7320 | 0.7390 | 0.7435 | 0.7502 | 0.7522 | 0.7519 | 0.7552 | 0.7574 | 0.7621 | 0.7728 | 0.7844 |
| Sep to Nov | -0.2711** | -0.3295*** | -0.3215*** | -0.2809*** | -0.2114*** | -0.2450*** | -0.2218*** | -0.1384* | -0.1274* | -0.1095 | -0.0833 | -0.0746 |
|  | (0.1123) | (0.1073) | (0.1012) | (0.0968) | (0.0818) | (0.0872) | (0.0809) | (0.0780) | (0.0704) | (0.0715) | (0.0640) | (0.0625) |
| N | 88,488 | 89,781 | 90,726 | 91,464 | 92,058 | 92,601 | 92,835 | 93,213 | 93,627 | 94,248 | 95,211 | 96,255 |
| Adj. R^2^ | 0.8085 | 0.8171 | 0.8243 | 0.8284 | 0.8337 | 0.8355 | 0.8362 | 0.8390 | 0.8411 | 0.8428 | 0.8470 | 0.8513 |
| Dec to Feb | -0.1584** | -0.1281** | -0.0851 | -0.0582 | -0.0422 | -0.0464 | -0.0631 | -0.0243 | -0.0297 | -0.0335 | 0.0126 | 0.0121 |
|  | (0.0745) | (0.0642) | (0.0590) | (0.0462) | (0.0424) | (0.0431) | (0.0545) | (0.0520) | (0.0459) | (0.0383) | (0.0337) | (0.0344) |
| N | 87,089 | 88,361 | 89,290 | 90,016 | 90,600 | 91,134 | 91,364 | 91,736 | 92,143 | 92,754 | 93,701 | 94,728 |
| Adj. R^2^ | 0.8662 | 0.8763 | 0.8878 | 0.8963 | 0.8995 | 0.9007 | 0.9004 | 0.9004 | 0.9028 | 0.9044 | 0.9060 | 0.9099 |
| Panel G: NPP (net primary productivity, kg*C/m^2^) | | | | | | | | | | | | |
| Mar to May | -0.0040 | -0.0061 | -0.0068* | -0.0051 | -0.0041 | -0.0031 | -0.0015 | -0.0002 | 0.0003 | 0.0002 | 0.0000 | -0.0000 |
|  | (0.0041) | (0.0039) | (0.0036) | (0.0033) | (0.0030) | (0.0030) | (0.0030) | (0.0030) | (0.0028) | (0.0026) | (0.0024) | (0.0022) |
| N | 88,479 | 89,769 | 90,717 | 91,464 | 92,058 | 92,601 | 92,835 | 93,213 | 93,627 | 94,239 | 95,211 | 96,255 |
| Adj. R^2^ | 0.9579 | 0.9626 | 0.9688 | 0.9736 | 0.9780 | 0.9786 | 0.9784 | 0.9797 | 0.9807 | 0.9824 | 0.9838 | 0.9871 |
| Jun to Aug | -0.0035 | -0.0057 | -0.0063* | -0.0047 | -0.0041 | -0.0030 | -0.0015 | -0.0002 | 0.0003 | 0.0001 | -0.0002 | -0.0002 |
|  | (0.0040) | (0.0038) | (0.0036) | (0.0032) | (0.0029) | (0.0029) | (0.0030) | (0.0029) | (0.0028) | (0.0026) | (0.0024) | (0.0022) |
| N | 88,479 | 89,769 | 90,717 | 91,464 | 92,058 | 92,601 | 92,835 | 93,213 | 93,627 | 94,239 | 95,211 | 96,255 |
| Adj. R^2^ | 0.9579 | 0.9626 | 0.9688 | 0.9736 | 0.9780 | 0.9786 | 0.9784 | 0.9797 | 0.9807 | 0.9824 | 0.9838 | 0.9871 |
| Sep to Nov | -0.0036 | -0.0057 | -0.0061* | -0.0047 | -0.0041 | -0.0032 | -0.0016 | -0.0002 | 0.0003 | 0.0003 | -0.0000 | 0.0001 |
|  | (0.0040) | (0.0038) | (0.0036) | (0.0032) | (0.0029) | (0.0029) | (0.0030) | (0.0029) | (0.0028) | (0.0026) | (0.0024) | (0.0022) |
| N | 88,479 | 89,769 | 90,717 | 91,464 | 92,058 | 92,601 | 92,835 | 93,213 | 93,627 | 94,239 | 95,211 | 96,255 |
| Adj. R^2^ | 0.9579 | 0.9626 | 0.9688 | 0.9736 | 0.9780 | 0.9786 | 0.9785 | 0.9798 | 0.9808 | 0.9824 | 0.9838 | 0.9872 |
| Dec to Feb | -0.0038 | -0.0058 | -0.0064* | -0.0048 | -0.0041 | -0.0031 | -0.0016 | -0.0002 | 0.0002 | 0.0002 | -0.0001 | -0.0000 |
|  | (0.0039) | (0.0036) | (0.0034) | (0.0031) | (0.0028) | (0.0028) | (0.0028) | (0.0028) | (0.0027) | (0.0025) | (0.0023) | (0.0021) |
| N | 88,479 | 89,769 | 90,717 | 91,464 | 92,058 | 92,601 | 92,835 | 93,213 | 93,627 | 94,239 | 95,211 | 96,255 |
| Adj. R^2^ | 0.9579 | 0.9626 | 0.9688 | 0.9736 | 0.9779 | 0.9786 | 0.9784 | 0.9797 | 0.9807 | 0.9823 | 0.9837 | 0.9871 |
| Panel H: PTC (percentage of tree cover, %) | | | | | | | | | | | | |
| Mar to May | -0.0801 | -0.2007 | -0.0221 | 0.0277 | -0.0245 | -0.1821 | -0.2382 | -0.1525 | -0.2364 | -0.2963 | -0.0915 | -0.1143 |
|  | (0.4376) | (0.3790) | (0.3299) | (0.2916) | (0.2692) | (0.2653) | (0.2544) | (0.2497) | (0.2314) | (0.2222) | (0.2078) | (0.1807) |
| N | 93,678 | 94,803 | 95,337 | 95,745 | 95,964 | 96,255 | 96,489 | 96,489 | 96,651 | 96,705 | 96,975 | 97,137 |
| Adj. R^2^ | 0.8830 | 0.8993 | 0.9136 | 0.9251 | 0.9360 | 0.9393 | 0.9412 | 0.9433 | 0.9458 | 0.9500 | 0.9526 | 0.9594 |
| Jun to Aug | 0.0486 | -0.1100 | 0.0602 | 0.0722 | -0.0036 | -0.1566 | -0.2045 | -0.1048 | -0.1857 | -0.2671 | -0.0746 | -0.0816 |
|  | (0.4333) | (0.3750) | (0.3274) | (0.2892) | (0.2666) | (0.2609) | (0.2494) | (0.2450) | (0.2281) | (0.2189) | (0.2031) | (0.1761) |
| N | 93,678 | 94,803 | 95,337 | 95,745 | 95,964 | 96,255 | 96,489 | 96,489 | 96,651 | 96,705 | 96,975 | 97,137 |
| Adj. R^2^ | 0.8830 | 0.8993 | 0.9137 | 0.9251 | 0.9360 | 0.9394 | 0.9412 | 0.9433 | 0.9458 | 0.9500 | 0.9526 | 0.9594 |
| Sep to Nov | -0.0076 | -0.1148 | 0.0841 | 0.0983 | 0.0094 | -0.1549 | -0.2196 | -0.1196 | -0.1989 | -0.2781 | -0.0716 | -0.0716 |
|  | (0.4318) | (0.3752) | (0.3263) | (0.2886) | (0.2667) | (0.2613) | (0.2499) | (0.2453) | (0.2280) | (0.2195) | (0.2032) | (0.1762) |
| N | 93,678 | 94,803 | 95,337 | 95,745 | 95,964 | 96,255 | 96,489 | 96,489 | 96,651 | 96,705 | 96,975 | 97,137 |
| Adj. R^2^ | 0.8830 | 0.8993 | 0.9137 | 0.9252 | 0.9361 | 0.9394 | 0.9413 | 0.9433 | 0.9459 | 0.9500 | 0.9527 | 0.9595 |
| Dec to Feb | -0.0657 | -0.1855 | -0.0208 | 0.0027 | -0.0461 | -0.1885 | -0.2422 | -0.1583 | -0.2328 | -0.2826 | -0.0947 | -0.0972 |
|  | (0.4092) | (0.3557) | (0.3105) | (0.2751) | (0.2532) | (0.2496) | (0.2402) | (0.2356) | (0.2179) | (0.2093) | (0.1957) | (0.1697) |
| N | 93,678 | 94,803 | 95,337 | 95,745 | 95,964 | 96,255 | 96,489 | 96,489 | 96,651 | 96,705 | 96,975 | 97,137 |
| Adj. R^2^ | 0.8830 | 0.8992 | 0.9136 | 0.9251 | 0.9360 | 0.9393 | 0.9412 | 0.9433 | 0.9458 | 0.9500 | 0.9526 | 0.9594 |
| Panel I: PNTV (percentage of non-tree vegetation, %) | | | | | | | | | | | | |
| Mar to May | -0.9455 | -1.1397* | -0.9932* | -1.7927** | -0.8646* | -0.6513 | -0.5189 | -0.4227 | -0.4457 | -0.1396 | 0.0273 | -0.0692 |
|  | (0.6753) | (0.6078) | (0.5481) | (0.7749) | (0.5230) | (0.5082) | (0.4910) | (0.4729) | (0.4331) | (0.4094) | (0.3640) | (0.2898) |
| N | 93,678 | 94,803 | 95,337 | 90,690 | 95,964 | 96,255 | 96,489 | 96,489 | 96,651 | 96,705 | 96,975 | 97,137 |
| Adj. R^2^ | 0.7762 | 0.8215 | 0.8417 | 0.5488 | 0.8690 | 0.8769 | 0.8825 | 0.8893 | 0.8955 | 0.9002 | 0.9150 | 0.9299 |
| Jun to Aug | -1.0610 | -1.2236** | -1.0371* | -1.6330** | -0.8887* | -0.7145 | -0.5724 | -0.4963 | -0.5270 | -0.2292 | -0.0635 | -0.1426 |
|  | (0.6686) | (0.6050) | (0.5461) | (0.7731) | (0.5182) | (0.5025) | (0.4851) | (0.4683) | (0.4290) | (0.4052) | (0.3583) | (0.2861) |
| N | 93,678 | 94,803 | 95,337 | 90,690 | 95,964 | 96,255 | 96,489 | 96,489 | 96,651 | 96,705 | 96,975 | 97,137 |
| Adj. R^2^ | 0.7759 | 0.8212 | 0.8414 | 0.5487 | 0.8686 | 0.8765 | 0.8822 | 0.8890 | 0.8953 | 0.8999 | 0.9147 | 0.9296 |
| Sep to Nov | -1.0111 | -1.2207** | -1.0245* | -1.7179** | -0.8676* | -0.6891 | -0.5345 | -0.4794 | -0.5201 | -0.2225 | -0.0671 | -0.1342 |
|  | (0.6720) | (0.6070) | (0.5468) | (0.7731) | (0.5196) | (0.5028) | (0.4858) | (0.4676) | (0.4271) | (0.4029) | (0.3560) | (0.2846) |
| N | 93,678 | 94,803 | 95,337 | 90,690 | 95,964 | 96,255 | 96,489 | 96,489 | 96,651 | 96,705 | 96,975 | 97,137 |
| Adj. R^2^ | 0.7759 | 0.8213 | 0.8414 | 0.5488 | 0.8686 | 0.8765 | 0.8821 | 0.8891 | 0.8953 | 0.9000 | 0.9148 | 0.9297 |
| Dec to Feb | -0.9311 | -1.1231* | -0.9401* | -1.7326** | -0.7993 | -0.6222 | -0.4882 | -0.4123 | -0.4282 | -0.1447 | 0.0253 | -0.0558 |
|  | (0.6506) | (0.5837) | (0.5226) | (0.7460) | (0.5018) | (0.4869) | (0.4710) | (0.4524) | (0.4120) | (0.3882) | (0.3458) | (0.2734) |
| N | 93,678 | 94,803 | 95,337 | 90,690 | 95,964 | 96,255 | 96,489 | 96,489 | 96,651 | 96,705 | 96,975 | 97,137 |
| Adj. R^2^ | 0.7759 | 0.8212 | 0.8413 | 0.5488 | 0.8686 | 0.8764 | 0.8821 | 0.8889 | 0.8951 | 0.8998 | 0.9145 | 0.9294 |
| Panel J: PNV (percentage of non-vegetation, %) | | | | | | | | | | | | |
| Mar to May | 1.0255 | 1.3404** | 1.0153* | 0.6334 | 0.8891* | 0.8334* | 0.7571 | 0.5752 | 0.6820 | 0.4359 | 0.0641 | 0.1836 |
|  | (0.6735) | (0.6085) | (0.5667) | (0.5235) | (0.5155) | (0.4982) | (0.4775) | (0.4635) | (0.4289) | (0.3969) | (0.3411) | (0.2765) |
| N | 93,678 | 94,803 | 95,337 | 95,745 | 95,964 | 96,255 | 96,489 | 96,489 | 96,651 | 96,705 | 96,975 | 97,137 |
| Adj. R^2^ | 0.8855 | 0.9065 | 0.9147 | 0.9238 | 0.9300 | 0.9329 | 0.9361 | 0.9399 | 0.9436 | 0.9468 | 0.9558 | 0.9632 |
| Jun to Aug | 1.0124 | 1.3335** | 0.9769* | 0.6280 | 0.8923* | 0.8711* | 0.7769 | 0.6011 | 0.7127* | 0.4963 | 0.1381 | 0.2242 |
|  | (0.6664) | (0.6052) | (0.5650) | (0.5223) | (0.5123) | (0.4949) | (0.4736) | (0.4608) | (0.4269) | (0.3955) | (0.3395) | (0.2777) |
| N | 93,678 | 94,803 | 95,337 | 95,745 | 95,964 | 96,255 | 96,489 | 96,489 | 96,651 | 96,705 | 96,975 | 97,137 |
| Adj. R^2^ | 0.8853 | 0.9063 | 0.9145 | 0.9236 | 0.9298 | 0.9327 | 0.9358 | 0.9397 | 0.9434 | 0.9466 | 0.9556 | 0.9630 |
| Sep to Nov | 1.0186 | 1.3356** | 0.9404* | 0.6159 | 0.8582* | 0.8440* | 0.7541 | 0.5990 | 0.7190* | 0.5006 | 0.1387 | 0.2058 |
|  | (0.6671) | (0.6075) | (0.5656) | (0.5215) | (0.5124) | (0.4928) | (0.4716) | (0.4576) | (0.4227) | (0.3910) | (0.3353) | (0.2750) |
| N | 93,678 | 94,803 | 95,337 | 95,745 | 95,964 | 96,255 | 96,489 | 96,489 | 96,651 | 96,705 | 96,975 | 97,137 |
| Adj. R^2^ | 0.8854 | 0.9064 | 0.9146 | 0.9237 | 0.9299 | 0.9327 | 0.9359 | 0.9398 | 0.9435 | 0.9467 | 0.9558 | 0.9632 |
| Dec to Feb | 0.9967 | 1.3086** | 0.9609* | 0.6270 | 0.8455* | 0.8106* | 0.7304 | 0.5706 | 0.6609 | 0.4273 | 0.0693 | 0.1529 |
|  | (0.6500) | (0.5850) | (0.5415) | (0.5005) | (0.4954) | (0.4782) | (0.4585) | (0.4435) | (0.4084) | (0.3762) | (0.3238) | (0.2606) |
| N | 93,678 | 94,803 | 95,337 | 95,745 | 95,964 | 96,255 | 96,489 | 96,489 | 96,651 | 96,705 | 96,975 | 97,137 |
| Adj. R^2^ | 0.8853 | 0.9062 | 0.9144 | 0.9235 | 0.9297 | 0.9326 | 0.9358 | 0.9396 | 0.9433 | 0.9465 | 0.9555 | 0.9629 |
|  |  |  |  |  |  |  |  |  |  |  |  |  |
| Controls | × | × | × | × | × | × | × | × | × | × | × | × |
| Year×County FE | × | × | × | × | × | × | × | × | × | × | × | × |
| Month FE | × | × | × | × | × | × | × | × | × | × | × | × |
| Terrain FE | × | × | × | × | × | × | × | × | × | × | × | × |

Note: The symbols ∗, ∗∗, and ∗∗∗ indicate significance levels at 10%, 5%, and 1%, respectively. "FE" stands for "fixed effects". Clustered standard errors at the wind farm level are shown in parentheses. Each panel depicts a single regression at the plant-year level, utilizing a buffer difference model to estimate the causal effect of wind farms on plant diversity indicators. The buffer model controls for the spatial dependence of observations, allowing for more accurate estimates of the treatment effect.

**Supplementary Table 7. Heterogeneous effects of elevation of wind farm on plant diversity by distance.**

|  | (1) | (2) | (3) | (4) | (5) | (6) | (7) | (8) | (9) | (10) | (11) | (12) |
| --- | --- | --- | --- | --- | --- | --- | --- | --- | --- | --- | --- | --- |
|  | 0-1 km | 1-2 km | 2-3 km | 3-4 km | 4-5 km | 5-6 km | 6-7 km | 7-8 km | 8-9 km | 9-10 km | 10-15 km | 15-20 km |
| Panel A: NDVI (normalized difference vegetation index) | | | | | | | | | | | | |
| EV less than 500m | -0.0160*** | -0.0219*** | -0.0203*** | -0.0139*** | -0.0146*** | -0.0124*** | -0.0087* | -0.0052 | -0.0078** | -0.0061 | -0.0016 | -0.0031 |
|  | (0.0051) | (0.0057) | (0.0057) | (0.0053) | (0.0050) | (0.0046) | (0.0047) | (0.0043) | (0.0039) | (0.0038) | (0.0032) | (0.0026) |
| N | 169,583 | 173,760 | 176,253 | 178,094 | 179,064 | 180,220 | 181,148 | 181,148 | 181,790 | 182,004 | 183,074 | 183,716 |
| Adj. R^2^ | 0.7576 | 0.7617 | 0.7680 | 0.7737 | 0.7771 | 0.7830 | 0.7845 | 0.7867 | 0.7889 | 0.7905 | 0.7957 | 0.7982 |
| EV b/w 500m and 1,500m | -0.0048 | -0.0057 | -0.0051 | -0.0055 | -0.0054 | -0.0062 | -0.0040 | -0.0028 | -0.0025 | -0.0025 | -0.0013 | -0.0016 |
|  | (0.0060) | (0.0047) | (0.0041) | (0.0039) | (0.0041) | (0.0039) | (0.0038) | (0.0035) | (0.0032) | (0.0032) | (0.0029) | (0.0024) |
| N | 102,500 | 102,500 | 102,500 | 102,500 | 102,500 | 102,500 | 102,500 | 102,500 | 102,500 | 102,500 | 102,500 | 102,500 |
| Adj. R^2^ | 0.8049 | 0.8106 | 0.8139 | 0.8158 | 0.8173 | 0.8187 | 0.8203 | 0.8220 | 0.8231 | 0.8244 | 0.8298 | 0.8346 |
| EV larger than 1,500m | -0.0019 | -0.0026 | 0.0009 | 0.0021 | 0.0021 | 0.0034 | 0.0012 | 0.0012 | 0.0006 | -0.0008 | -0.0010 | -0.0008 |
|  | (0.0044) | (0.0041) | (0.0039) | (0.0035) | (0.0030) | (0.0030) | (0.0029) | (0.0028) | (0.0028) | (0.0027) | (0.0025) | (0.0023) |
| N | 99,250 | 99,250 | 99,250 | 99,250 | 99,250 | 99,250 | 99,250 | 99,250 | 99,250 | 99,250 | 99,250 | 99,250 |
| Adj. R^2^ | 0.7425 | 0.7589 | 0.7723 | 0.7810 | 0.7878 | 0.7918 | 0.7960 | 0.7996 | 0.8022 | 0.8062 | 0.8133 | 0.8217 |
| Panel B: EVI (enhanced vegetation index) | | | | | | | | | | | | |
| EV less than 500m | -0.0119*** | -0.0147*** | -0.0141*** | -0.0101*** | -0.0103*** | -0.0082*** | -0.0059** | -0.0035 | 0.0134 | -0.0042* | -0.0010 | -0.0016 |
|  | (0.0033) | (0.0035) | (0.0035) | (0.0033) | (0.0030) | (0.0028) | (0.0029) | (0.0027) | (0.0094) | (0.0024) | (0.0020) | (0.0017) |
| N | 169,583 | 173,760 | 176,253 | 178,094 | 179,064 | 180,220 | 181,148 | 181,148 | 181,551 | 182,004 | 183,074 | 183,716 |
| Adj. R^2^ | 0.7528 | 0.7598 | 0.7667 | 0.7738 | 0.7794 | 0.7855 | 0.7871 | 0.7895 | 0.5622 | 0.7940 | 0.8007 | 0.8052 |
| EV b/w 500m and 1,500m | -0.0059 | -0.0060* | -0.0055* | -0.0053* | -0.0049* | -0.0045 | -0.0035 | -0.0031 | -0.0029 | -0.0026 | -0.0019 | -0.0017 |
|  | (0.0042) | (0.0034) | (0.0029) | (0.0028) | (0.0029) | (0.0027) | (0.0025) | (0.0023) | (0.0110) | (0.0018) | (0.0017) | (0.0013) |
| N | 102,500 | 102,500 | 102,500 | 102,500 | 102,500 | 102,500 | 102,500 | 102,500 | 102,500 | 102,500 | 102,500 | 102,500 |
| Adj. R^2^ | 0.7698 | 0.7783 | 0.7830 | 0.7869 | 0.7910 | 0.7940 | 0.7963 | 0.7984 | 0.5779 | 0.8019 | 0.8088 | 0.8144 |
| EV larger than 1,500m | -0.0002 | -0.0004 | 0.0006 | 0.0010 | 0.0006 | 0.0015 | 0.0008 | 0.0008 | 0.0030 | 0.0004 | -0.0001 | -0.0002 |
|  | (0.0025) | (0.0022) | (0.0020) | (0.0019) | (0.0017) | (0.0017) | (0.0016) | (0.0015) | (0.0112) | (0.0015) | (0.0013) | (0.0012) |
| N | 99,250 | 99,250 | 99,250 | 99,250 | 99,250 | 99,250 | 99,250 | 99,250 | 99,197 | 99,250 | 99,250 | 99,250 |
| Adj. R^2^ | 0.7633 | 0.7782 | 0.7899 | 0.7987 | 0.8045 | 0.8077 | 0.8108 | 0.8139 | 0.5670 | 0.8193 | 0.8282 | 0.8378 |
| Panel C: FPAR (fraction of photosynthetically active radiation) | | | | | | | | | | | | |
| EV less than 500m | -1.4696*** | -1.5593*** | -1.4235*** | -1.2792*** | -1.0642*** | -0.8388*** | -0.5503** | -0.3825 | -0.3391 | -0.2177 | -0.1727 | -0.1594 |
|  | (0.4170) | (0.3759) | (0.3523) | (0.3153) | (0.3006) | (0.2940) | (0.2750) | (0.2641) | (0.2514) | (0.2641) | (0.2260) | (0.2021) |
| N | 167,154 | 171,284 | 174,032 | 175,710 | 177,566 | 178,970 | 179,898 | 180,148 | 180,790 | 181,504 | 182,574 | 183,716 |
| Adj. R^2^ | 0.7229 | 0.7338 | 0.7405 | 0.7439 | 0.7466 | 0.7495 | 0.7505 | 0.7520 | 0.7537 | 0.7540 | 0.7588 | 0.7629 |
| EV b/w 500m and 1,500m | -0.1034 | -0.2536 | -0.4372 | -0.1350 | -0.1904 | -0.0806 | -0.0631 | -0.0676 | 0.0270 | 0.0945 | -0.1434 | -0.1886 |
|  | (0.3346) | (0.3224) | (0.3434) | (0.2655) | (0.2534) | (0.2661) | (0.2547) | (0.2467) | (0.2544) | (0.3088) | (0.2256) | (0.2058) |
| N | 90,250 | 91,000 | 91,750 | 92,500 | 93,000 | 94,000 | 93,500 | 94,500 | 95,250 | 96,750 | 97,750 | 100,500 |
| Adj. R^2^ | 0.7391 | 0.7503 | 0.7542 | 0.7601 | 0.7607 | 0.7605 | 0.7634 | 0.7653 | 0.7664 | 0.7672 | 0.7740 | 0.7789 |
| EV larger than 1,500m | -0.6193 | -0.2648 | -0.0279 | 0.4287 | 0.1520 | 0.3163 | 0.3298 | 0.3271 | 0.2372 | -0.0142 | 0.0679 | 0.0984 |
|  | (0.5654) | (0.4888) | (0.4917) | (0.3934) | (0.3483) | (0.3436) | (0.3441) | (0.3419) | (0.3171) | (0.2793) | (0.2541) | (0.2373) |
| N | 93,750 | 94,000 | 94,250 | 94,750 | 94,750 | 94,500 | 95,000 | 95,250 | 95,500 | 95,750 | 97,500 | 97,750 |
| Adj. R^2^ | 0.6506 | 0.6842 | 0.6944 | 0.7133 | 0.7157 | 0.7166 | 0.7234 | 0.7238 | 0.7284 | 0.7322 | 0.7436 | 0.7534 |
| Panel D: LAI (leaf area index, %) | | | | | | | | | | | | |
| EV less than 500m | -0.0502*** | -0.0567*** | -0.0502*** | -0.0433*** | -0.0359*** | -0.0350*** | -0.0298*** | -0.0247** | -0.0163* | -0.0060 | -0.0128 | -0.0091 |
|  | (0.0176) | (0.0149) | (0.0158) | (0.0116) | (0.0118) | (0.0127) | (0.0102) | (0.0100) | (0.0098) | (0.0127) | (0.0093) | (0.0089) |
| N | 167,154 | 171,284 | 174,032 | 175,710 | 177,566 | 178,970 | 179,898 | 180,148 | 180,790 | 181,504 | 182,574 | 183,716 |
| Adj. R^2^ | 0.6871 | 0.6995 | 0.7040 | 0.7153 | 0.7160 | 0.7199 | 0.7244 | 0.7259 | 0.7279 | 0.7228 | 0.7334 | 0.7401 |
| EV b/w 500m and 1,500m | -0.0105 | -0.0141 | -0.0258 | -0.0096 | -0.0159 | -0.0045 | -0.0055 | -0.0064 | -0.0024 | -0.0040 | -0.0059 | -0.0078 |
|  | (0.0145) | (0.0131) | (0.0160) | (0.0099) | (0.0098) | (0.0092) | (0.0092) | (0.0088) | (0.0090) | (0.0100) | (0.0081) | (0.0071) |
| N | 90,250 | 91,000 | 91,750 | 92,500 | 93,000 | 94,000 | 93,500 | 94,500 | 95,250 | 96,750 | 97,750 | 100,500 |
| Adj. R^2^ | 0.6541 | 0.6765 | 0.6809 | 0.6888 | 0.6802 | 0.6743 | 0.6784 | 0.6818 | 0.6853 | 0.6889 | 0.6977 | 0.7008 |
| EV larger than 1,500m | -0.0561 | -0.0103 | -0.0014 | 0.0396 | 0.0107 | 0.0262 | 0.0361 | 0.0370 | 0.0268 | 0.0055 | 0.0221 | 0.0237* |
|  | (0.0464) | (0.0395) | (0.0383) | (0.0281) | (0.0243) | (0.0237) | (0.0245) | (0.0252) | (0.0226) | (0.0180) | (0.0162) | (0.0134) |
| N | 93,750 | 94,000 | 94,250 | 94,750 | 94,750 | 94,500 | 95,000 | 95,250 | 95,500 | 95,750 | 97,500 | 97,750 |
| Adj. R^2^ | 0.6568 | 0.7355 | 0.7397 | 0.7784 | 0.7828 | 0.7776 | 0.7872 | 0.7829 | 0.7928 | 0.7989 | 0.8148 | 0.8297 |
| Panel E: GPP (gross primary product, g*C/m^2^) | | | | | | | | | | | | |
| EV less than 500m | -0.7436*** | -0.7887*** | -0.7749*** | -0.7449*** | -0.5544*** | -0.5411*** | -0.3862** | -0.1725 | -0.2178 | -0.2200 | -0.0762 | -0.0598 |
|  | (0.2479) | (0.2165) | (0.2102) | (0.1977) | (0.1699) | (0.1834) | (0.1681) | (0.1734) | (0.1499) | (0.1425) | (0.1358) | (0.1258) |
| N | 167,817 | 171,964 | 174,724 | 176,409 | 178,273 | 179,683 | 180,615 | 180,866 | 181,511 | 182,228 | 183,303 | 184,450 |
| Adj. R^2^ | 0.7580 | 0.7657 | 0.7723 | 0.7745 | 0.7784 | 0.7805 | 0.7806 | 0.7825 | 0.7847 | 0.7848 | 0.7878 | 0.7918 |
| EV b/w 500m and 1,500m | 0.0881 | -0.0387 | -0.1536 | -0.0257 | -0.0872 | -0.1072 | -0.0296 | -0.0378 | 0.0122 | 0.0821 | -0.0061 | -0.0414 |
|  | (0.1939) | (0.1861) | (0.1941) | (0.1640) | (0.1581) | (0.1649) | (0.1592) | (0.1570) | (0.1548) | (0.1722) | (0.1430) | (0.1302) |
| N | 90,611 | 91,364 | 92,117 | 92,870 | 93,372 | 94,376 | 93,874 | 94,878 | 95,631 | 97,137 | 98,141 | 100,902 |
| Adj. R^2^ | 0.7482 | 0.7527 | 0.7565 | 0.7606 | 0.7633 | 0.7645 | 0.7678 | 0.7679 | 0.7679 | 0.7677 | 0.7716 | 0.7720 |
| EV larger than 1,500m | 0.0445 | -0.0778 | 0.0856 | 0.1501 | 0.0894 | 0.0918 | 0.1009 | 0.0539 | 0.0325 | -0.0487 | -0.0472 | -0.0572 |
|  | (0.2836) | (0.2626) | (0.2467) | (0.2086) | (0.1906) | (0.1829) | (0.1774) | (0.1680) | (0.1571) | (0.1464) | (0.1312) | (0.1209) |
| N | 94,125 | 94,376 | 94,627 | 95,129 | 95,129 | 94,878 | 95,380 | 95,631 | 95,882 | 96,133 | 97,890 | 98,141 |
| Adj. R^2^ | 0.6948 | 0.7097 | 0.7224 | 0.7306 | 0.7374 | 0.7406 | 0.7426 | 0.7458 | 0.7482 | 0.7521 | 0.7606 | 0.7689 |
| Panel F: NP (net photosynthesis, g*C/m^2^) | | | | | | | | | | | | |
| EV less than 500m | -0.0166*** | -0.0160*** | -0.0149*** | -0.0153*** | -0.0115*** | -0.0071* | -0.0032 | -0.0002 | -0.0007 | -0.0013 | -0.0003 | 0.0001 |
|  | (0.0060) | (0.0054) | (0.0049) | (0.0045) | (0.0040) | (0.0039) | (0.0041) | (0.0040) | (0.0040) | (0.0035) | (0.0033) | (0.0031) |
| N | 168,444 | 172,596 | 175,380 | 177,108 | 178,980 | 180,396 | 181,332 | 181,584 | 182,232 | 182,916 | 184,032 | 185,184 |
| Adj. R^2^ | 0.9442 | 0.9471 | 0.9611 | 0.9654 | 0.9712 | 0.9727 | 0.9720 | 0.9734 | 0.9738 | 0.9759 | 0.9756 | 0.9816 |
| EV b/w 500m and 1,500m | 0.0053 | 0.0026 | -0.0009 | 0.0018 | -0.0010 | -0.0020 | 0.0001 | -0.0002 | 0.0007 | 0.0030 | 0.0004 | -0.0013 |
|  | (0.0048) | (0.0046) | (0.0051) | (0.0043) | (0.0042) | (0.0045) | (0.0043) | (0.0044) | (0.0042) | (0.0044) | (0.0040) | (0.0037) |
| N | 90,972 | 91,728 | 92,484 | 93,240 | 93,744 | 94,752 | 94,248 | 95,256 | 96,012 | 97,524 | 98,532 | 101,304 |
| Adj. R^2^ | 0.9850 | 0.9866 | 0.9850 | 0.9854 | 0.9863 | 0.9866 | 0.9871 | 0.9866 | 0.9863 | 0.9871 | 0.9884 | 0.9902 |
| EV larger than 1,500m | 0.0057 | 0.0003 | 0.0038 | 0.0062 | 0.0041 | 0.0045 | 0.0047 | 0.0036 | 0.0030 | 0.0005 | -0.0004 | -0.0012 |
|  | (0.0072) | (0.0067) | (0.0063) | (0.0057) | (0.0052) | (0.0051) | (0.0051) | (0.0047) | (0.0042) | (0.0039) | (0.0037) | (0.0032) |
| N | 94,500 | 94,752 | 95,004 | 95,508 | 95,508 | 95,256 | 95,760 | 96,012 | 96,264 | 96,516 | 98,280 | 98,532 |
| Adj. R^2^ | 0.9695 | 0.9739 | 0.9780 | 0.9823 | 0.9864 | 0.9867 | 0.9877 | 0.9898 | 0.9912 | 0.9926 | 0.9933 | 0.9948 |
| Panel G: NPP (net primary productivity, kg*C/m^2^) | | | | | | | | | | | | |
| EV less than 500m | -0.3770** | -0.3619** | -0.3829*** | -0.3830*** | -0.2731** | -0.2706** | -0.1628 | -0.0302 | -0.0612 | -0.0745 | 0.0236 | 0.0236 |
|  | (0.1646) | (0.1423) | (0.1385) | (0.1352) | (0.1158) | (0.1270) | (0.1139) | (0.1156) | (0.1017) | (0.0986) | (0.0929) | (0.0862) |
| N | 167,817 | 171,964 | 174,724 | 176,409 | 178,273 | 179,683 | 180,615 | 180,866 | 181,511 | 182,228 | 183,303 | 184,450 |
| Adj. R^2^ | 0.6548 | 0.6659 | 0.6738 | 0.6746 | 0.6792 | 0.6795 | 0.6786 | 0.6804 | 0.6809 | 0.6813 | 0.6819 | 0.6839 |
| EV b/w 500m and 1,500m | 0.1217 | 0.0492 | -0.0445 | 0.0405 | -0.0219 | -0.0490 | 0.0085 | 0.0068 | 0.0351 | 0.0977 | 0.0323 | 0.0010 |
|  | (0.1320) | (0.1271) | (0.1363) | (0.1164) | (0.1125) | (0.1193) | (0.1152) | (0.1165) | (0.1125) | (0.1208) | (0.1066) | (0.0986) |
| N | 90,611 | 91,364 | 92,117 | 92,870 | 93,372 | 94,376 | 93,874 | 94,878 | 95,631 | 97,137 | 98,141 | 100,902 |
| Adj. R^2^ | 0.7065 | 0.7113 | 0.7176 | 0.7228 | 0.7260 | 0.7274 | 0.7308 | 0.7301 | 0.7295 | 0.7301 | 0.7320 | 0.7305 |
| EV larger than 1,500m | 0.0424 | -0.0910 | -0.0043 | 0.0661 | 0.0270 | 0.0286 | 0.0348 | 0.0034 | -0.0105 | -0.0675 | -0.0870 | -0.1057 |
|  | (0.1857) | (0.1701) | (0.1619) | (0.1436) | (0.1326) | (0.1295) | (0.1269) | (0.1184) | (0.1067) | (0.0992) | (0.0916) | (0.0824) |
| N | 94,125 | 94,376 | 94,627 | 95,129 | 95,129 | 94,878 | 95,380 | 95,631 | 95,882 | 96,133 | 97,890 | 98,141 |
| Adj. R^2^ | 0.6776 | 0.6914 | 0.7038 | 0.7104 | 0.7163 | 0.7194 | 0.7214 | 0.7247 | 0.7277 | 0.7310 | 0.7393 | 0.7480 |
| Panel H: PTC (percentage of tree cover, %) | | | | | | | | | | | | |
| EV less than 500m | 0.1358 | -0.1418 | 0.0217 | 0.1116 | 0.2134 | -0.1820 | -0.0651 | 0.1371 | -0.0583 | -0.1994 | -0.0551 | 0.0976 |
|  | (0.5545) | (0.4634) | (0.4069) | (0.3570) | (0.3431) | (0.3410) | (0.3064) | (0.2970) | (0.2838) | (0.2832) | (0.2490) | (0.2220) |
| N | 171,348 | 175,848 | 177,984 | 179,616 | 180,492 | 181,656 | 182,592 | 182,592 | 183,240 | 183,456 | 184,536 | 185,184 |
| Adj. R^2^ | 0.8797 | 0.8848 | 0.8963 | 0.9094 | 0.9088 | 0.9125 | 0.9110 | 0.9150 | 0.9197 | 0.9225 | 0.9235 | 0.9428 |
| EV b/w 500m and 1,500m | 0.3150 | 0.1247 | 0.2076 | 0.0543 | 0.0184 | -0.1446 | -0.0297 | 0.0362 | 0.0429 | -0.0286 | -0.0805 | -0.1381 |
|  | (0.3622) | (0.3199) | (0.2565) | (0.2165) | (0.2155) | (0.2020) | (0.2283) | (0.1914) | (0.1882) | (0.2165) | (0.1872) | (0.1511) |
| N | 103,320 | 103,320 | 103,320 | 103,320 | 103,320 | 103,320 | 103,320 | 103,320 | 103,320 | 103,320 | 103,320 | 103,320 |
| Adj. R^2^ | 0.9837 | 0.9826 | 0.9852 | 0.9888 | 0.9904 | 0.9904 | 0.9894 | 0.9893 | 0.9906 | 0.9901 | 0.9919 | 0.9923 |
| EV larger than 1,500m | -0.5037 | -0.4170 | 0.2963 | 0.4529 | 0.4096 | 0.3278 | 0.0652 | -0.0232 | -0.3990 | -0.5436 | 0.0618 | 0.2201 |
|  | (0.9227) | (0.7642) | (0.6768) | (0.5545) | (0.4591) | (0.4117) | (0.4027) | (0.4416) | (0.4287) | (0.3643) | (0.3021) | (0.2951) |
| N | 100,044 | 100,044 | 100,044 | 100,044 | 100,044 | 100,044 | 100,044 | 100,044 | 100,044 | 100,044 | 100,044 | 100,044 |
| Adj. R^2^ | 0.8577 | 0.8875 | 0.9114 | 0.9261 | 0.9470 | 0.9528 | 0.9575 | 0.9603 | 0.9624 | 0.9710 | 0.9762 | 0.9807 |
| Panel I: PNTV (percentage of non-tree vegetation, %) | | | | | | | | | | | | |
| EV less than 500m | -1.7545** | -2.4195*** | -2.2231*** | -1.0446 | -1.9306*** | -1.1886** | -1.0539** | -0.8695* | -1.1179** | -0.7047 | -0.3656 | -0.7576** |
|  | (0.7124) | (0.6774) | (0.6372) | (1.2163) | (0.5376) | (0.5250) | (0.5036) | (0.5030) | (0.4602) | (0.4582) | (0.3787) | (0.3083) |
| N | 171,348 | 175,848 | 177,984 | 177,036 | 180,492 | 181,656 | 182,592 | 182,592 | 183,240 | 183,456 | 184,536 | 185,184 |
| Adj. R^2^ | 0.7668 | 0.8154 | 0.8383 | 0.5250 | 0.8663 | 0.8777 | 0.8836 | 0.8901 | 0.9001 | 0.9053 | 0.9182 | 0.9357 |
| EV b/w 500m and 1,500m | -0.7442 | -0.6982 | -0.5718 | -1.3188 | -0.4975 | -0.5399 | -0.3832 | -0.2295 | -0.2112 | -0.0418 | 0.0656 | 0.1221 |
|  | (1.4842) | (1.2897) | (1.1006) | (1.2304) | (1.0911) | (1.0745) | (1.0390) | (0.9711) | (0.9114) | (0.8101) | (0.6958) | (0.5167) |
| N | 103,320 | 103,320 | 103,320 | 89,208 | 103,320 | 103,320 | 103,320 | 103,320 | 103,320 | 103,320 | 103,320 | 103,320 |
| Adj. R^2^ | 0.8596 | 0.8823 | 0.8922 | 0.6756 | 0.9021 | 0.9028 | 0.9063 | 0.9123 | 0.9152 | 0.9176 | 0.9292 | 0.9390 |
| EV larger than 1,500m | 0.0860 | 0.1926 | 0.1494 | -0.7777 | -0.1371 | -0.1797 | -0.1436 | -0.2759 | 0.0170 | 0.2598 | -0.3310 | -0.6380** |
|  | (0.9174) | (0.7052) | (0.5859) | (1.0215) | (0.5397) | (0.4714) | (0.4440) | (0.4218) | (0.3835) | (0.3587) | (0.3278) | (0.2691) |
| N | 100,044 | 100,044 | 100,044 | 96,516 | 100,044 | 100,044 | 100,044 | 100,044 | 100,044 | 100,044 | 100,044 | 100,044 |
| Adj. R^2^ | 0.7858 | 0.8456 | 0.8818 | 0.6372 | 0.9110 | 0.9197 | 0.9251 | 0.9304 | 0.9365 | 0.9409 | 0.9544 | 0.9686 |
| Panel J: PNV (percentage of non-vegetation, %) | | | | | | | | | | | | |
| EV less than 500m | 1.6187** | 2.5613*** | 2.2015*** | 1.4015** | 1.7172*** | 1.3706*** | 1.1190** | 0.7324 | 1.1762*** | 0.9041** | 0.4207 | 0.6600** |
|  | (0.6758) | (0.6821) | (0.6681) | (0.5928) | (0.5289) | (0.5100) | (0.4783) | (0.4787) | (0.4272) | (0.4353) | (0.3784) | (0.3191) |
| N | 171,348 | 175,848 | 177,984 | 179,616 | 180,492 | 181,656 | 182,592 | 182,592 | 183,240 | 183,456 | 184,536 | 185,184 |
| Adj. R^2^ | 0.8632 | 0.8889 | 0.9019 | 0.9138 | 0.9209 | 0.9280 | 0.9325 | 0.9374 | 0.9435 | 0.9468 | 0.9559 | 0.9645 |
| EV b/w 500m and 1,500m | 0.4292 | 0.5735 | 0.3642 | 0.1231 | 0.4791 | 0.6846 | 0.4130 | 0.1933 | 0.1683 | 0.0704 | 0.0148 | 0.0160 |
|  | (1.5458) | (1.3236) | (1.1183) | (1.0526) | (1.0874) | (1.0675) | (1.0456) | (0.9683) | (0.9027) | (0.8151) | (0.7013) | (0.5316) |
| N | 103,320 | 103,320 | 103,320 | 103,320 | 103,320 | 103,320 | 103,320 | 103,320 | 103,320 | 103,320 | 103,320 | 103,320 |
| Adj. R^2^ | 0.9231 | 0.9364 | 0.9402 | 0.9450 | 0.9464 | 0.9466 | 0.9480 | 0.9508 | 0.9521 | 0.9533 | 0.9593 | 0.9647 |
| EV larger than 1,500m | 0.4177 | 0.2244 | -0.4457 | -0.4258 | -0.2725 | -0.1481 | 0.0784 | 0.2991 | 0.3820 | 0.2839 | 0.2691 | 0.4178 |
|  | (0.7255) | (0.6058) | (0.5632) | (0.5602) | (0.4899) | (0.4934) | (0.4927) | (0.5099) | (0.5099) | (0.4669) | (0.3889) | (0.3316) |
| N | 100,044 | 100,044 | 100,044 | 100,044 | 100,044 | 100,044 | 100,044 | 100,044 | 100,044 | 100,044 | 100,044 | 100,044 |
| Adj. R^2^ | 0.9362 | 0.9479 | 0.9563 | 0.9628 | 0.9687 | 0.9690 | 0.9711 | 0.9736 | 0.9749 | 0.9772 | 0.9838 | 0.9877 |
|  |  |  |  |  |  |  |  |  |  |  |  |  |
| Controls | × | × | × | × | × | × | × | × | × | × | × | × |
| Year×County FE | × | × | × | × | × | × | × | × | × | × | × | × |
| Month FE | × | × | × | × | × | × | × | × | × | × | × | × |
| Terrain FE | × | × | × | × | × | × | × | × | × | × | × | × |

Note: The symbols ∗, ∗∗, and ∗∗∗ indicate significance levels at 10%, 5%, and 1%, respectively. "FE" stands for "fixed effects". Clustered standard errors at the wind farm level are shown in parentheses. Each panel depicts a single regression at the plant-year level, utilizing a buffer difference model to estimate the causal effect of wind farms on plant diversity indicators. The buffer model controls for the spatial dependence of observations, allowing for more accurate estimates of the treatment effect.

**Supplementary Table 8. Heterogeneous effects of land type of wind farm on plant diversity by distance.**

|  | (1) | (2) | (3) | (4) | (5) | (6) | (7) | (8) | (9) | (10) | (11) | (12) |
| --- | --- | --- | --- | --- | --- | --- | --- | --- | --- | --- | --- | --- |
|  | 0-1 km | 1-2 km | 2-3 km | 3-4 km | 4-5 km | 5-6 km | 6-7 km | 7-8 km | 8-9 km | 9-10 km | 10-15 km | 15-20 km |
| Panel A: NDVI (normalized difference vegetation index) | | | | | | | | | | | | |
| Plain | -0.0141* | -0.0201** | -0.0216** | -0.0211** | -0.0220*** | -0.0235*** | -0.0204*** | -0.0162** | -0.0163*** | -0.0154*** | -0.0099** | -0.0098** |
|  | (0.0075) | (0.0091) | (0.0090) | (0.0089) | (0.0080) | (0.0071) | (0.0068) | (0.0065) | (0.0060) | (0.0058) | (0.0048) | (0.0042) |
| N | 97,796 | 99,254 | 99,964 | 100,874 | 101,666 | 102,548 | 102,786 | 102,786 | 102,786 | 102,786 | 103,000 | 103,000 |
| Adj. R^2^ | 0.7174 | 0.7218 | 0.7304 | 0.7368 | 0.7428 | 0.7510 | 0.7538 | 0.7561 | 0.7602 | 0.7637 | 0.7710 | 0.7801 |
| Terrace | -0.0122** | -0.0128** | -0.0100** | -0.0046 | -0.0024 | 0.0011 | -0.0092 | -0.0039 | -0.0022 | 0.0002 | 0.0032 | 0.0052 |
|  | (0.0058) | (0.0055) | (0.0048) | (0.0046) | (0.0048) | (0.0047) | (0.0114) | (0.0088) | (0.0078) | (0.0053) | (0.0045) | (0.0039) |
| N | 66,692 | 67,144 | 67,369 | 67,584 | 67,798 | 67,798 | 68,036 | 68,036 | 68,250 | 68,250 | 68,250 | 68,250 |
| Adj. R^2^ | 0.7659 | 0.7720 | 0.7786 | 0.7824 | 0.7834 | 0.7854 | 0.7849 | 0.7892 | 0.7916 | 0.7940 | 0.7980 | 0.8040 |
| Hill | -0.0005 | -0.0060 | -0.0099** | -0.0096** | -0.0091** | -0.0083** | -0.0073* | -0.0071* | -0.0068* | -0.0079** | -0.0042 | -0.0037 |
|  | (0.0045) | (0.0041) | (0.0040) | (0.0038) | (0.0038) | (0.0037) | (0.0037) | (0.0038) | (0.0038) | (0.0038) | (0.0030) | (0.0028) |
| N | 95,004 | 96,074 | 96,930 | 97,156 | 97,120 | 97,180 | 97,394 | 97,394 | 97,608 | 97,608 | 97,822 | 98,036 |
| Adj. R^2^ | 0.8099 | 0.8101 | 0.8099 | 0.8118 | 0.8135 | 0.8132 | 0.8133 | 0.8156 | 0.8149 | 0.8149 | 0.8197 | 0.8197 |
| Low relief mountain | 0.0011 | -0.0011 | -0.0007 | 0.0015 | 0.0006 | -0.0002 | -0.0017 | -0.0026 | -0.0014 | -0.0007 | -0.0045 | -0.0092** |
|  | (0.0080) | (0.0072) | (0.0076) | (0.0074) | (0.0063) | (0.0063) | (0.0061) | (0.0058) | (0.0056) | (0.0054) | (0.0043) | (0.0042) |
| N | 55,002 | 55,216 | 55,216 | 55,216 | 55,216 | 55,216 | 55,216 | 55,216 | 55,430 | 55,430 | 55,858 | 56,072 |
| Adj. R^2^ | 0.7448 | 0.7513 | 0.7553 | 0.7571 | 0.7639 | 0.7682 | 0.7692 | 0.7706 | 0.7712 | 0.7719 | 0.7772 | 0.7826 |
| Intermediate relief mountain | -0.0014 | 0.0028 | 0.0069 | 0.0085 | 0.0089 | 0.0075 | 0.0059 | 0.0032 | 0.0025 | 0.0035 | 0.0043 | 0.0029 |
|  | (0.0091) | (0.0079) | (0.0062) | (0.0057) | (0.0056) | (0.0056) | (0.0060) | (0.0064) | (0.0056) | (0.0049) | (0.0039) | (0.0035) |
| N | 48,822 | 48,822 | 48,822 | 48,822 | 48,822 | 48,822 | 48,822 | 48,822 | 48,822 | 49,036 | 49,036 | 49,036 |
| Adj. R^2^ | 0.5885 | 0.6143 | 0.6406 | 0.6528 | 0.6596 | 0.6628 | 0.6671 | 0.6706 | 0.6759 | 0.6813 | 0.6927 | 0.7041 |
| Panel B: EVI (enhanced vegetation index) | | | | | | | | | | | | |
| Plain | -0.0091* | -0.0128** | -0.0147*** | -0.0150*** | -0.0149*** | -0.0151*** | -0.0130*** | -0.0103** | 0.0065 | -0.0106*** | -0.0065** | -0.0057** |
|  | (0.0052) | (0.0058) | (0.0056) | (0.0056) | (0.0050) | (0.0044) | (0.0042) | (0.0041) | (0.0137) | (0.0037) | (0.0029) | (0.0026) |
| N | 97,796 | 99,254 | 99,964 | 100,874 | 101,666 | 102,548 | 102,786 | 102,786 | 102,786 | 102,786 | 103,000 | 103,000 |
| Adj. R^2^ | 0.6950 | 0.7015 | 0.7110 | 0.7181 | 0.7249 | 0.7326 | 0.7362 | 0.7380 | 0.5310 | 0.7449 | 0.7531 | 0.7629 |
| Terrace | -0.0111** | -0.0112** | -0.0085** | -0.0044 | -0.0027 | -0.0006 | -0.0082 | -0.0044 | 0.0179 | -0.0004 | 0.0024 | 0.0036 |
|  | (0.0047) | (0.0043) | (0.0038) | (0.0037) | (0.0039) | (0.0036) | (0.0084) | (0.0065) | (0.0156) | (0.0039) | (0.0032) | (0.0027) |
| N | 66,692 | 67,144 | 67,369 | 67,584 | 67,798 | 67,798 | 68,036 | 68,036 | 68,011 | 68,250 | 68,250 | 68,250 |
| Adj. R^2^ | 0.7491 | 0.7580 | 0.7659 | 0.7697 | 0.7716 | 0.7745 | 0.7736 | 0.7777 | 0.5784 | 0.7825 | 0.7870 | 0.7923 |
| Hill | -0.0028 | -0.0052** | -0.0073*** | -0.0063*** | -0.0055** | -0.0044** | -0.0037* | -0.0041* | 0.0039 | -0.0042* | -0.0015 | -0.0017 |
|  | (0.0022) | (0.0022) | (0.0023) | (0.0023) | (0.0022) | (0.0021) | (0.0023) | (0.0024) | (0.0139) | (0.0023) | (0.0018) | (0.0017) |
| N | 95,004 | 96,074 | 96,930 | 97,156 | 97,120 | 97,180 | 97,394 | 97,394 | 97,608 | 97,608 | 97,822 | 98,036 |
| Adj. R^2^ | 0.8029 | 0.8036 | 0.8026 | 0.8054 | 0.8084 | 0.8081 | 0.8081 | 0.8116 | 0.6173 | 0.8122 | 0.8186 | 0.8206 |
| Low relief mountain | -0.0012 | -0.0011 | -0.0014 | -0.0003 | -0.0010 | -0.0012 | -0.0018 | -0.0021 | 0.0080 | -0.0011 | -0.0028 | -0.0045** |
|  | (0.0043) | (0.0040) | (0.0040) | (0.0037) | (0.0031) | (0.0031) | (0.0031) | (0.0030) | (0.0162) | (0.0028) | (0.0021) | (0.0022) |
| N | 55,002 | 55,216 | 55,216 | 55,216 | 55,216 | 55,216 | 55,216 | 55,216 | 55,430 | 55,430 | 55,858 | 56,072 |
| Adj. R^2^ | 0.8018 | 0.8077 | 0.8100 | 0.8132 | 0.8195 | 0.8238 | 0.8246 | 0.8253 | 0.7151 | 0.8253 | 0.8316 | 0.8361 |
| Intermediate relief mountain | -0.0022 | -0.0001 | 0.0012 | 0.0007 | 0.0001 | 0.0010 | 0.0013 | 0.0008 | -0.0277** | 0.0015 | 0.0018 | 0.0013 |
|  | (0.0047) | (0.0037) | (0.0030) | (0.0028) | (0.0029) | (0.0029) | (0.0031) | (0.0032) | (0.0131) | (0.0026) | (0.0020) | (0.0018) |
| N | 48,822 | 48,822 | 48,822 | 48,822 | 48,822 | 48,822 | 48,822 | 48,822 | 48,769 | 49,036 | 49,036 | 49,036 |
| Adj. R^2^ | 0.7247 | 0.7456 | 0.7627 | 0.7713 | 0.7770 | 0.7785 | 0.7809 | 0.7851 | 0.6873 | 0.7911 | 0.8004 | 0.8097 |
| Panel C: FPAR (fraction of photosynthetically active radiation) | | | | | | | | | | | | |
| Plain | -0.8509* | -1.1783** | -1.4034*** | -1.4859*** | -1.2056*** | -1.1967*** | -1.0168** | -0.8409** | -0.7594* | -0.7556* | -0.7553** | -0.5862* |
|  | (0.5085) | (0.5508) | (0.5317) | (0.5145) | (0.4532) | (0.4248) | (0.4263) | (0.3986) | (0.3927) | (0.3947) | (0.3188) | (0.2989) |
| N | 85,656 | 88,026 | 89,264 | 90,252 | 92,168 | 93,798 | 94,286 | 94,786 | 95,536 | 97,536 | 99,000 | 100,750 |
| Adj. R^2^ | 0.6861 | 0.6971 | 0.7055 | 0.7098 | 0.7133 | 0.7171 | 0.7176 | 0.7217 | 0.7240 | 0.7252 | 0.7325 | 0.7409 |
| Terrace | -1.4826** | -1.0217** | -0.6969** | -0.3597 | -0.2206 | 0.3660 | -0.1726 | -0.1336 | 0.0966 | 0.3606 | 0.4236 | 0.3019 |
|  | (0.5725) | (0.4263) | (0.3452) | (0.3316) | (0.3635) | (0.3595) | (0.6687) | (0.5621) | (0.5019) | (0.3722) | (0.3077) | (0.3422) |
| N | 61,192 | 61,644 | 62,358 | 63,584 | 63,548 | 63,798 | 63,786 | 64,536 | 65,000 | 65,250 | 66,750 | 67,750 |
| Adj. R^2^ | 0.7348 | 0.7474 | 0.7498 | 0.7541 | 0.7557 | 0.7590 | 0.7626 | 0.7603 | 0.7608 | 0.7595 | 0.7625 | 0.7692 |
| Hill | -0.0505 | -0.3316 | -0.5526 | -0.5224 | -0.4748 | -0.4018 | -0.3774 | -0.3781 | -0.2720 | -0.2415 | 0.0420 | -0.0517 |
|  | (0.4413) | (0.3825) | (0.3580) | (0.3396) | (0.3283) | (0.3215) | (0.3142) | (0.3194) | (0.3270) | (0.3303) | (0.2790) | (0.2425) |
| N | 92,754 | 93,824 | 94,680 | 94,906 | 95,370 | 95,430 | 95,644 | 95,894 | 96,108 | 96,108 | 96,322 | 97,286 |
| Adj. R^2^ | 0.7665 | 0.7745 | 0.7743 | 0.7774 | 0.7782 | 0.7771 | 0.7772 | 0.7774 | 0.7803 | 0.7811 | 0.7852 | 0.7847 |
| Low relief mountain | -0.1469 | -0.0637 | -0.4566 | 0.1476 | 0.2165 | 0.0976 | -0.1548 | -0.0507 | 0.0155 | 0.0283 | -0.1910 | -0.6961* |
|  | (0.7446) | (0.6404) | (0.7369) | (0.6373) | (0.5400) | (0.5451) | (0.5855) | (0.5532) | (0.5373) | (0.4970) | (0.4113) | (0.3755) |
| N | 54,752 | 55,216 | 55,216 | 55,216 | 55,216 | 55,216 | 55,216 | 55,216 | 55,430 | 55,430 | 55,858 | 56,072 |
| Adj. R^2^ | 0.6950 | 0.6984 | 0.7020 | 0.7086 | 0.7086 | 0.7114 | 0.7143 | 0.7135 | 0.7117 | 0.7117 | 0.7155 | 0.7238 |
| Intermediate relief mountain | -0.5985 | -0.0929 | -0.2134 | 0.6451 | 0.0792 | 0.3906 | 0.2605 | 0.3616 | 0.0480 | 0.0402 | 0.2999 | 0.1467 |
|  | (1.1788) | (0.8060) | (0.7086) | (0.6216) | (0.5447) | (0.6371) | (0.7535) | (0.7705) | (0.5600) | (0.4994) | (0.3881) | (0.3457) |
| N | 48,822 | 48,822 | 48,822 | 48,822 | 48,822 | 48,822 | 48,822 | 48,822 | 48,822 | 49,036 | 49,036 | 49,036 |
| Adj. R^2^ | 0.5444 | 0.5776 | 0.6020 | 0.6167 | 0.6224 | 0.6156 | 0.6157 | 0.6170 | 0.6233 | 0.6272 | 0.6351 | 0.6436 |
| Panel D: LAI (leaf area index, %) | | | | | | | | | | | | |
| Plain | -0.0244 | -0.0357** | -0.0290* | -0.0414*** | -0.0356*** | -0.0264* | -0.0271** | -0.0243* | -0.0175 | -0.0182 | -0.0197** | -0.0157* |
|  | (0.0153) | (0.0158) | (0.0165) | (0.0142) | (0.0127) | (0.0158) | (0.0123) | (0.0126) | (0.0116) | (0.0113) | (0.0092) | (0.0088) |
| N | 85,656 | 88,026 | 89,264 | 90,252 | 92,168 | 93,798 | 94,286 | 94,786 | 95,536 | 97,536 | 99,000 | 100,750 |
| Adj. R^2^ | 0.5638 | 0.5791 | 0.5897 | 0.5945 | 0.5994 | 0.6026 | 0.6006 | 0.6070 | 0.6090 | 0.6215 | 0.6132 | 0.6214 |
| Terrace | -0.0276 | -0.0204 | -0.0111 | -0.0014 | 0.0018 | 0.0170 | 0.0061 | -0.0002 | 0.0053 | 0.0162 | 0.0214* | 0.0153 |
|  | (0.0194) | (0.0153) | (0.0128) | (0.0135) | (0.0146) | (0.0133) | (0.0227) | (0.0197) | (0.0190) | (0.0142) | (0.0117) | (0.0130) |
| N | 61,192 | 61,644 | 62,358 | 63,584 | 63,548 | 63,798 | 63,786 | 64,536 | 65,000 | 65,250 | 66,750 | 67,750 |
| Adj. R^2^ | 0.6270 | 0.6493 | 0.6372 | 0.6608 | 0.6594 | 0.6764 | 0.6912 | 0.6712 | 0.6765 | 0.6670 | 0.6715 | 0.6760 |
| Hill | 0.0046 | -0.0137 | -0.0189 | -0.0153 | -0.0135 | -0.0065 | -0.0107 | -0.0102 | -0.0030 | -0.0002 | 0.0045 | -0.0014 |
|  | (0.0187) | (0.0167) | (0.0148) | (0.0137) | (0.0136) | (0.0134) | (0.0128) | (0.0123) | (0.0135) | (0.0136) | (0.0112) | (0.0093) |
| N | 92,754 | 93,824 | 94,680 | 94,906 | 95,370 | 95,430 | 95,644 | 95,894 | 96,108 | 96,108 | 96,322 | 97,286 |
| Adj. R^2^ | 0.7107 | 0.7352 | 0.7183 | 0.7314 | 0.7275 | 0.7217 | 0.7218 | 0.7212 | 0.7292 | 0.7280 | 0.7353 | 0.7410 |
| Low relief mountain | -0.0241 | -0.0069 | -0.0602 | -0.0017 | 0.0124 | 0.0077 | -0.0098 | 0.0080 | 0.0046 | 0.0057 | -0.0041 | -0.0253 |
|  | (0.0418) | (0.0302) | (0.0497) | (0.0318) | (0.0252) | (0.0253) | (0.0306) | (0.0284) | (0.0285) | (0.0249) | (0.0202) | (0.0175) |
| N | 54,752 | 55,216 | 55,216 | 55,216 | 55,216 | 55,216 | 55,216 | 55,216 | 55,430 | 55,430 | 55,858 | 56,072 |
| Adj. R^2^ | 0.7365 | 0.7295 | 0.7248 | 0.7487 | 0.7384 | 0.7443 | 0.7543 | 0.7471 | 0.7418 | 0.7431 | 0.7564 | 0.7680 |
| Intermediate relief mountain | -0.0771 | -0.0299 | -0.0500 | 0.0450 | -0.0212 | 0.0131 | 0.0070 | 0.0346 | 0.0030 | -0.0028 | 0.0249 | 0.0117 |
|  | (0.0966) | (0.0669) | (0.0518) | (0.0411) | (0.0323) | (0.0436) | (0.0511) | (0.0542) | (0.0368) | (0.0320) | (0.0258) | (0.0208) |
| N | 48,822 | 48,822 | 48,822 | 48,822 | 48,822 | 48,822 | 48,822 | 48,822 | 48,822 | 49,036 | 49,036 | 49,036 |
| Adj. R^2^ | 0.5970 | 0.6681 | 0.7023 | 0.7289 | 0.7359 | 0.7218 | 0.7193 | 0.7189 | 0.7306 | 0.7349 | 0.7469 | 0.7595 |
| Panel E: GPP (gross primary product, g*C/m^2^) | | | | | | | | | | | | |
| Plain | -0.4764* | -0.6817** | -0.8148*** | -0.8392*** | -0.6232** | -0.7160*** | -0.4923** | -0.4353** | -0.4579** | -0.3637* | -0.3935** | -0.2420 |
|  | (0.2683) | (0.2906) | (0.2866) | (0.2893) | (0.2414) | (0.2329) | (0.2263) | (0.2025) | (0.2046) | (0.1995) | (0.1636) | (0.1565) |
| N | 85,998 | 88,377 | 89,620 | 90,612 | 92,536 | 94,173 | 94,663 | 95,165 | 95,918 | 97,926 | 99,396 | 101,153 |
| Adj. R^2^ | 0.7150 | 0.7227 | 0.7321 | 0.7359 | 0.7409 | 0.7441 | 0.7455 | 0.7497 | 0.7515 | 0.7502 | 0.7562 | 0.7606 |
| Terrace | -0.6229* | -0.4172 | -0.1682 | -0.0407 | -0.0053 | 0.3498 | -0.1563 | -0.1247 | 0.1046 | 0.2851 | 0.4209* | 0.3273 |
|  | (0.3638) | (0.2924) | (0.2243) | (0.2132) | (0.2468) | (0.2497) | (0.5090) | (0.5132) | (0.3819) | (0.2470) | (0.2287) | (0.2274) |
| N | 61,436 | 61,890 | 62,607 | 63,838 | 63,802 | 64,053 | 64,041 | 64,794 | 65,260 | 65,511 | 67,017 | 68,021 |
| Adj. R^2^ | 0.7646 | 0.7749 | 0.7781 | 0.7782 | 0.7798 | 0.7790 | 0.7799 | 0.7781 | 0.7786 | 0.7776 | 0.7758 | 0.7772 |
| Hill | 0.1256 | -0.0176 | -0.1990 | -0.1770 | -0.1056 | -0.0856 | -0.0787 | -0.0682 | -0.0241 | -0.0511 | 0.1168 | 0.0839 |
|  | (0.2367) | (0.2079) | (0.1984) | (0.1841) | (0.1781) | (0.1694) | (0.1589) | (0.1619) | (0.1642) | (0.1682) | (0.1622) | (0.1392) |
| N | 93,123 | 94,198 | 95,058 | 95,285 | 95,751 | 95,811 | 96,026 | 96,277 | 96,492 | 96,492 | 96,707 | 97,675 |
| Adj. R^2^ | 0.7862 | 0.7913 | 0.7940 | 0.7943 | 0.7963 | 0.7982 | 0.7986 | 0.7985 | 0.7994 | 0.7999 | 0.8031 | 0.8020 |
| Low relief mountain | 0.2871 | -0.0298 | -0.1273 | -0.1400 | -0.1496 | -0.2940 | -0.3071 | -0.3134 | -0.2297 | -0.2583 | -0.3476* | -0.7175*** |
|  | (0.4633) | (0.4108) | (0.4104) | (0.3824) | (0.3344) | (0.3126) | (0.3191) | (0.3110) | (0.2810) | (0.2539) | (0.2079) | (0.2147) |
| N | 54,970 | 55,436 | 55,436 | 55,436 | 55,436 | 55,436 | 55,436 | 55,436 | 55,651 | 55,651 | 56,081 | 56,296 |
| Adj. R^2^ | 0.7398 | 0.7451 | 0.7503 | 0.7532 | 0.7582 | 0.7617 | 0.7627 | 0.7630 | 0.7629 | 0.7639 | 0.7669 | 0.7743 |
| Intermediate relief mountain | -0.2778 | -0.3073 | -0.1339 | -0.0013 | -0.0073 | -0.0839 | -0.0698 | -0.2262 | -0.2570 | -0.1385 | -0.0523 | -0.1676 |
|  | (0.5152) | (0.4442) | (0.3394) | (0.3020) | (0.2923) | (0.3260) | (0.3448) | (0.3348) | (0.2740) | (0.2403) | (0.2069) | (0.1868) |
| N | 49,017 | 49,017 | 49,017 | 49,017 | 49,017 | 49,017 | 49,017 | 49,017 | 49,017 | 49,232 | 49,232 | 49,232 |
| Adj. R^2^ | 0.6445 | 0.6603 | 0.6792 | 0.6875 | 0.6935 | 0.6959 | 0.6971 | 0.7003 | 0.7036 | 0.7086 | 0.7138 | 0.7216 |
| Panel F: NP (net photosynthesis, g*C/m^2^) | | | | | | | | | | | | |
| Plain | -0.2650* | -0.3690** | -0.4721*** | -0.4822*** | -0.3535** | -0.4486*** | -0.2964** | -0.2771** | -0.2927** | -0.2220* | -0.2359** | -0.1306 |
|  | (0.1600) | (0.1726) | (0.1690) | (0.1759) | (0.1559) | (0.1549) | (0.1462) | (0.1274) | (0.1284) | (0.1267) | (0.1034) | (0.0997) |
| N | 85,998 | 88,377 | 89,620 | 90,612 | 92,536 | 94,173 | 94,663 | 95,165 | 95,918 | 97,926 | 99,396 | 101,153 |
| Adj. R^2^ | 0.6301 | 0.6408 | 0.6515 | 0.6572 | 0.6650 | 0.6689 | 0.6679 | 0.6709 | 0.6728 | 0.6728 | 0.6796 | 0.6825 |
| Terrace | -0.3722 | -0.2270 | -0.0580 | 0.0293 | 0.0339 | 0.3024 | -0.0442 | 0.0239 | 0.1804 | 0.2772 | 0.3329* | 0.2469 |
|  | (0.2566) | (0.2064) | (0.1671) | (0.1595) | (0.1784) | (0.1865) | (0.3172) | (0.3166) | (0.2300) | (0.1700) | (0.1709) | (0.1612) |
| N | 61,436 | 61,890 | 62,607 | 63,838 | 63,802 | 64,053 | 64,041 | 64,794 | 65,260 | 65,511 | 67,017 | 68,021 |
| Adj. R^2^ | 0.7293 | 0.7394 | 0.7411 | 0.7386 | 0.7387 | 0.7358 | 0.7365 | 0.7336 | 0.7329 | 0.7327 | 0.7305 | 0.7269 |
| Hill | 0.1459 | 0.0760 | -0.0603 | -0.0616 | -0.0473 | -0.0391 | -0.0243 | -0.0262 | -0.0112 | -0.0377 | 0.0384 | 0.0309 |
|  | (0.1504) | (0.1395) | (0.1310) | (0.1256) | (0.1219) | (0.1163) | (0.1110) | (0.1128) | (0.1165) | (0.1198) | (0.1198) | (0.1002) |
| N | 93,123 | 94,198 | 95,058 | 95,285 | 95,751 | 95,811 | 96,026 | 96,277 | 96,492 | 96,492 | 96,707 | 97,675 |
| Adj. R^2^ | 0.7173 | 0.7244 | 0.7276 | 0.7267 | 0.7292 | 0.7307 | 0.7317 | 0.7311 | 0.7298 | 0.7299 | 0.7319 | 0.7317 |
| Low relief mountain | 0.2232 | -0.0680 | -0.1387 | -0.1320 | -0.1319 | -0.2347 | -0.2233 | -0.2393 | -0.2093 | -0.2501 | -0.2991** | -0.5544*** |
|  | (0.3528) | (0.3019) | (0.2996) | (0.2734) | (0.2423) | (0.2242) | (0.2312) | (0.2247) | (0.1869) | (0.1652) | (0.1337) | (0.1415) |
| N | 54,970 | 55,436 | 55,436 | 55,436 | 55,436 | 55,436 | 55,436 | 55,436 | 55,651 | 55,651 | 56,081 | 56,296 |
| Adj. R^2^ | 0.6599 | 0.6720 | 0.6805 | 0.6845 | 0.6867 | 0.6888 | 0.6894 | 0.6902 | 0.6899 | 0.6924 | 0.6917 | 0.6968 |
| Intermediate relief mountain | -0.1161 | -0.2122 | -0.1198 | 0.0025 | 0.0206 | -0.0422 | -0.0550 | -0.1551 | -0.1937 | -0.1332 | -0.0618 | -0.1399 |
|  | (0.3267) | (0.2789) | (0.2248) | (0.2119) | (0.2059) | (0.2277) | (0.2386) | (0.2288) | (0.1843) | (0.1617) | (0.1452) | (0.1374) |
| N | 49,017 | 49,017 | 49,017 | 49,017 | 49,017 | 49,017 | 49,017 | 49,017 | 49,017 | 49,232 | 49,232 | 49,232 |
| Adj. R^2^ | 0.5711 | 0.5849 | 0.6057 | 0.6146 | 0.6215 | 0.6253 | 0.6272 | 0.6316 | 0.6350 | 0.6388 | 0.6432 | 0.6520 |
| Panel G: NPP (net primary productivity, kg*C/m^2^) | | | | | | | | | | | | |
| Plain | -0.0112* | -0.0143** | -0.0188*** | -0.0189*** | -0.0154*** | -0.0155*** | -0.0109** | -0.0093** | -0.0097** | -0.0088* | -0.0100*** | -0.0070** |
|  | (0.0059) | (0.0062) | (0.0060) | (0.0060) | (0.0054) | (0.0050) | (0.0053) | (0.0047) | (0.0044) | (0.0045) | (0.0036) | (0.0035) |
| N | 86,340 | 88,728 | 89,976 | 90,972 | 92,904 | 94,548 | 95,040 | 95,544 | 96,300 | 98,316 | 99,792 | 101,556 |
| Adj. R^2^ | 0.9207 | 0.9298 | 0.9421 | 0.9400 | 0.9460 | 0.9545 | 0.9528 | 0.9568 | 0.9582 | 0.9596 | 0.9711 | 0.9772 |
| Terrace | -0.0167* | -0.0094 | -0.0046 | -0.0012 | -0.0002 | 0.0092 | 0.0007 | 0.0067 | 0.0077 | 0.0108* | 0.0132** | 0.0093* |
|  | (0.0095) | (0.0077) | (0.0065) | (0.0060) | (0.0062) | (0.0067) | (0.0089) | (0.0066) | (0.0067) | (0.0057) | (0.0059) | (0.0055) |
| N | 61,680 | 62,136 | 62,856 | 64,092 | 64,056 | 64,308 | 64,296 | 65,052 | 65,520 | 65,772 | 67,284 | 68,292 |
| Adj. R^2^ | 0.9638 | 0.9763 | 0.9814 | 0.9835 | 0.9834 | 0.9824 | 0.9829 | 0.9846 | 0.9854 | 0.9863 | 0.9882 | 0.9908 |
| Hill | 0.0021 | 0.0006 | -0.0043 | -0.0044 | -0.0040 | -0.0040 | -0.0033 | -0.0037 | -0.0036 | -0.0043 | -0.0014 | -0.0009 |
|  | (0.0053) | (0.0049) | (0.0046) | (0.0043) | (0.0043) | (0.0040) | (0.0039) | (0.0039) | (0.0041) | (0.0042) | (0.0042) | (0.0035) |
| N | 93,492 | 94,572 | 95,436 | 95,664 | 96,132 | 96,192 | 96,408 | 96,660 | 96,876 | 96,876 | 97,092 | 98,064 |
| Adj. R^2^ | 0.9840 | 0.9826 | 0.9842 | 0.9843 | 0.9864 | 0.9864 | 0.9867 | 0.9871 | 0.9871 | 0.9870 | 0.9870 | 0.9874 |
| Low relief mountain | 0.0157 | 0.0025 | 0.0004 | 0.0025 | 0.0036 | 0.0011 | 0.0017 | 0.0007 | 0.0018 | 0.0007 | -0.0027 | -0.0116*** |
|  | (0.0131) | (0.0108) | (0.0111) | (0.0098) | (0.0087) | (0.0083) | (0.0086) | (0.0084) | (0.0070) | (0.0059) | (0.0046) | (0.0042) |
| N | 55,188 | 55,656 | 55,656 | 55,656 | 55,656 | 55,656 | 55,656 | 55,656 | 55,872 | 55,872 | 56,304 | 56,520 |
| Adj. R^2^ | 0.9860 | 0.9885 | 0.9853 | 0.9870 | 0.9895 | 0.9910 | 0.9926 | 0.9932 | 0.9942 | 0.9948 | 0.9916 | 0.9965 |
| Intermediate relief mountain | -0.0018 | -0.0062 | -0.0028 | 0.0032 | 0.0036 | 0.0010 | 0.0017 | -0.0021 | -0.0038 | -0.0016 | 0.0024 | -0.0013 |
|  | (0.0121) | (0.0105) | (0.0087) | (0.0084) | (0.0081) | (0.0088) | (0.0092) | (0.0084) | (0.0067) | (0.0058) | (0.0053) | (0.0050) |
| N | 49,212 | 49,212 | 49,212 | 49,212 | 49,212 | 49,212 | 49,212 | 49,212 | 49,212 | 49,392 | 49,428 | 49,428 |
| Adj. R^2^ | 0.9652 | 0.9733 | 0.9829 | 0.9871 | 0.9874 | 0.9851 | 0.9850 | 0.9871 | 0.9896 | 0.9923 | 0.9931 | 0.9934 |
| Panel H: PTC (percentage of tree cover, %) | | | | | | | | | | | | |
| Plain | -0.5731*** | -0.3279* | -0.2324 | 0.0001 | -0.0457 | -0.1646 | -0.1328 | 0.0003 | 0.0520 | 0.0648 | 0.0262 | -0.0292 |
|  | (0.2172) | (0.1809) | (0.1856) | (0.1856) | (0.1531) | (0.1487) | (0.1557) | (0.1612) | (0.1717) | (0.1554) | (0.1417) | (0.1458) |
| N | 99,024 | 100,752 | 101,100 | 101,784 | 102,480 | 103,368 | 103,608 | 103,608 | 103,608 | 103,608 | 103,824 | 103,824 |
| Adj. R^2^ | 0.9213 | 0.9424 | 0.9435 | 0.9493 | 0.9580 | 0.9593 | 0.9549 | 0.9559 | 0.9554 | 0.9578 | 0.9645 | 0.9585 |
| Terrace | 0.5646 | 0.4932 | 0.4491* | 0.2809 | 0.2862 | 0.2798 | 0.3631 | 0.3584 | 0.3253 | 0.2926 | 0.4246 | 0.2909 |
|  | (0.4250) | (0.3162) | (0.2549) | (0.2298) | (0.2427) | (0.2638) | (0.2766) | (0.2859) | (0.2926) | (0.2651) | (0.2920) | (0.2788) |
| N | 67,224 | 67,680 | 67,896 | 68,124 | 68,340 | 68,340 | 68,580 | 68,580 | 68,796 | 68,796 | 68,796 | 68,796 |
| Adj. R^2^ | 0.8966 | 0.9323 | 0.9460 | 0.9602 | 0.9514 | 0.9429 | 0.9381 | 0.9383 | 0.9339 | 0.9341 | 0.9490 | 0.9653 |
| Hill | 0.5954 | 0.0737 | -0.2344 | -0.3149 | -0.4556 | -0.5314 | -0.5312 | -0.3518 | -0.4289 | -0.4235 | -0.4689 | -0.4747 |
|  | (0.7989) | (0.7019) | (0.5677) | (0.5675) | (0.5768) | (0.5686) | (0.5450) | (0.5304) | (0.5177) | (0.5000) | (0.4292) | (0.3588) |
| N | 95,760 | 96,840 | 97,704 | 97,932 | 97,896 | 97,956 | 98,172 | 98,172 | 98,388 | 98,388 | 98,604 | 98,820 |
| Adj. R^2^ | 0.8999 | 0.8965 | 0.9022 | 0.9050 | 0.9043 | 0.9003 | 0.8994 | 0.9091 | 0.9152 | 0.9191 | 0.9214 | 0.9417 |
| Low relief mountain | 0.4625 | -0.1849 | 0.4042 | 0.7211 | 0.8846 | 0.7717 | 0.5356 | 0.2631 | 0.5659 | 0.4851 | 0.0483 | -0.8362 |
|  | (0.9845) | (0.9322) | (0.9987) | (0.9923) | (0.7685) | (0.8120) | (0.7895) | (0.7658) | (0.7422) | (0.6725) | (0.6184) | (0.6067) |
| N | 55,440 | 55,656 | 55,656 | 55,656 | 55,656 | 55,656 | 55,656 | 55,656 | 55,872 | 55,872 | 56,304 | 56,520 |
| Adj. R^2^ | 0.9746 | 0.9696 | 0.9679 | 0.9662 | 0.9744 | 0.9726 | 0.9760 | 0.9786 | 0.9800 | 0.9830 | 0.9757 | 0.9874 |
| Intermediate relief mountain | -1.2209 | -0.5464 | 0.2956 | 1.1565 | 1.2259 | 0.3298 | -0.0083 | -0.5024 | -0.6596 | -0.5909 | 0.3509 | 0.1890 |
|  | (2.0739) | (1.6589) | (1.2270) | (0.9603) | (0.7906) | (0.7405) | (0.8356) | (0.9210) | (0.8164) | (0.6495) | (0.5944) | (0.5038) |
| N | 49,212 | 49,212 | 49,212 | 49,212 | 49,212 | 49,212 | 49,212 | 49,212 | 49,212 | 49,428 | 49,428 | 49,428 |
| Adj. R^2^ | 0.8446 | 0.8805 | 0.9210 | 0.9450 | 0.9558 | 0.9562 | 0.9537 | 0.9531 | 0.9596 | 0.9671 | 0.9733 | 0.9812 |
| Panel I: PNTV (percentage of non-tree vegetation, %) | | | | | | | | | | | | |
| Plain | -2.4767** | -2.8335** | -2.7123** | -4.4809*** | -2.5494** | -2.8022*** | -2.4975*** | -2.2514** | -2.6347*** | -2.4264*** | -1.6943*** | -1.3727** |
|  | (1.2162) | (1.1851) | (1.1494) | (1.4203) | (1.0081) | (0.9630) | (0.9484) | (0.9265) | (0.8328) | (0.7646) | (0.6198) | (0.5534) |
| N | 99,024 | 100,752 | 101,100 | 90,444 | 102,480 | 103,368 | 103,608 | 103,608 | 103,608 | 103,608 | 103,824 | 103,824 |
| Adj. R^2^ | 0.8615 | 0.8778 | 0.8879 | 0.5859 | 0.9042 | 0.9073 | 0.9089 | 0.9140 | 0.9220 | 0.9257 | 0.9336 | 0.9472 |
| Terrace | 0.4591 | 0.6091 | 0.9810 | -1.4204 | 1.4242 | 1.6204 | 1.2289 | 1.4083 | 1.6275 | 1.6601 | 1.4943 | 1.6353* |
|  | (1.4251) | (1.2829) | (1.3599) | (2.2437) | (1.4367) | (1.3679) | (1.3880) | (1.4270) | (1.4476) | (1.4442) | (1.2271) | (0.8818) |
| N | 67,224 | 67,680 | 67,896 | 63,780 | 68,340 | 68,340 | 68,580 | 68,580 | 68,796 | 68,796 | 68,796 | 68,796 |
| Adj. R^2^ | 0.8997 | 0.9276 | 0.9320 | 0.7047 | 0.9418 | 0.9447 | 0.9458 | 0.9483 | 0.9483 | 0.9479 | 0.9589 | 0.9686 |
| Hill | -0.6484 | -0.5145 | -0.0054 | -1.5665* | 0.0989 | 0.3007 | 0.2533 | -0.0006 | 0.1697 | 0.2249 | 0.8856 | 0.7509 |
|  | (0.8430) | (0.7855) | (0.7255) | (0.9337) | (0.7340) | (0.7139) | (0.7245) | (0.6963) | (0.6854) | (0.6726) | (0.5981) | (0.5613) |
| N | 95,760 | 96,840 | 97,704 | 94,656 | 97,896 | 97,956 | 98,172 | 98,172 | 98,388 | 98,388 | 98,604 | 98,820 |
| Adj. R^2^ | 0.8349 | 0.8672 | 0.8816 | 0.6606 | 0.8925 | 0.8916 | 0.8964 | 0.9040 | 0.9056 | 0.9074 | 0.9235 | 0.9372 |
| Low relief mountain | 0.2445 | 0.0939 | -0.3273 | -1.5966 | -0.5073 | -1.1308* | -1.1686** | -0.8100 | -0.2371 | 0.2540 | 0.2000 | 0.1288 |
|  | (1.1126) | (0.8289) | (0.8103) | (1.4014) | (0.6929) | (0.6446) | (0.5679) | (0.5514) | (0.5519) | (0.5484) | (0.4490) | (0.4025) |
| N | 55,440 | 55,656 | 55,656 | 54,396 | 55,656 | 55,656 | 55,656 | 55,656 | 55,872 | 55,872 | 56,304 | 56,520 |
| Adj. R^2^ | 0.9124 | 0.9275 | 0.9349 | 0.8072 | 0.9451 | 0.9488 | 0.9557 | 0.9613 | 0.9648 | 0.9685 | 0.9691 | 0.9791 |
| Intermediate relief mountain | 1.6583 | 1.0744 | 0.6888 | 3.6592*** | -0.9649 | -0.3560 | -0.2371 | -0.2340 | -0.3812 | -0.2046 | -0.9154** | -0.7121** |
|  | (1.5927) | (1.1731) | (0.8833) | (1.4006) | (0.7131) | (0.6473) | (0.5884) | (0.5360) | (0.5031) | (0.5058) | (0.4631) | (0.3398) |
| N | 49,212 | 49,212 | 49,212 | 49,212 | 49,212 | 49,212 | 49,212 | 49,212 | 49,212 | 49,428 | 49,428 | 49,428 |
| Adj. R^2^ | 0.7612 | 0.8263 | 0.8768 | 0.7454 | 0.9062 | 0.9101 | 0.9152 | 0.9268 | 0.9385 | 0.9396 | 0.9522 | 0.9701 |
| Panel J: PNV (percentage of non-vegetation, %) | | | | | | | | | | | | |
| Plain | 3.0498** | 3.1614** | 2.9447** | 2.4819** | 2.5951** | 2.9667*** | 2.6303*** | 2.2510** | 2.5828*** | 2.3616*** | 1.6682*** | 1.4019** |
|  | (1.2266) | (1.2225) | (1.1990) | (1.1407) | (1.0390) | (0.9728) | (0.9493) | (0.9227) | (0.8330) | (0.7600) | (0.6381) | (0.5783) |
| N | 99,024 | 100,752 | 101,100 | 101,784 | 102,480 | 103,368 | 103,608 | 103,608 | 103,608 | 103,608 | 103,824 | 103,824 |
| Adj. R^2^ | 0.8866 | 0.8988 | 0.9079 | 0.9153 | 0.9232 | 0.9265 | 0.9280 | 0.9310 | 0.9363 | 0.9396 | 0.9468 | 0.9585 |
| Terrace | -1.0237 | -1.1024 | -1.4300 | -1.6443 | -1.7103 | -1.9002 | -1.5920 | -1.7667 | -1.9528 | -1.9527 | -1.9189 | -1.9262** |
|  | (1.3051) | (1.2152) | (1.3351) | (1.4148) | (1.4154) | (1.3403) | (1.3546) | (1.3983) | (1.4268) | (1.4164) | (1.1950) | (0.8305) |
| N | 67,224 | 67,680 | 67,896 | 68,124 | 68,340 | 68,340 | 68,580 | 68,580 | 68,796 | 68,796 | 68,796 | 68,796 |
| Adj. R^2^ | 0.9311 | 0.9509 | 0.9540 | 0.9577 | 0.9618 | 0.9641 | 0.9648 | 0.9674 | 0.9669 | 0.9671 | 0.9747 | 0.9821 |
| Hill | 0.0530 | 0.4408 | 0.2398 | 0.1276 | 0.3567 | 0.2307 | 0.2779 | 0.3524 | 0.2592 | 0.1987 | -0.4167 | -0.2763 |
|  | (0.7716) | (0.6701) | (0.6631) | (0.5773) | (0.5758) | (0.5456) | (0.5130) | (0.4717) | (0.4812) | (0.4914) | (0.4556) | (0.4393) |
| N | 95,760 | 96,840 | 97,704 | 97,932 | 97,896 | 97,956 | 98,172 | 98,172 | 98,388 | 98,388 | 98,604 | 98,820 |
| Adj. R^2^ | 0.9252 | 0.9388 | 0.9440 | 0.9518 | 0.9521 | 0.9525 | 0.9568 | 0.9604 | 0.9625 | 0.9640 | 0.9714 | 0.9745 |
| Low relief mountain | -0.7069 | 0.0911 | -0.0769 | -0.5592 | -0.3774 | 0.3591 | 0.6330 | 0.5469 | -0.3289 | -0.7391 | -0.2483 | 0.7074 |
|  | (1.5727) | (1.2371) | (1.2257) | (1.1891) | (1.0246) | (0.9973) | (0.9041) | (0.9150) | (0.8996) | (0.8291) | (0.6637) | (0.6477) |
| N | 55,440 | 55,656 | 55,656 | 55,656 | 55,656 | 55,656 | 55,656 | 55,656 | 55,872 | 55,872 | 56,304 | 56,520 |
| Adj. R^2^ | 0.9385 | 0.9501 | 0.9536 | 0.9550 | 0.9607 | 0.9629 | 0.9690 | 0.9709 | 0.9735 | 0.9778 | 0.9842 | 0.9876 |
| Intermediate relief mountain | -0.4373 | -0.5279 | -0.9844 | -0.3327 | -0.2610 | 0.0262 | 0.2454 | 0.7365 | 1.0408 | 0.7954 | 0.5645 | 0.5231 |
|  | (1.0441) | (1.0370) | (0.9796) | (0.9569) | (0.9167) | (0.8134) | (0.8311) | (0.9469) | (0.8405) | (0.7066) | (0.5536) | (0.4856) |
| N | 49,212 | 49,212 | 49,212 | 49,212 | 49,212 | 49,212 | 49,212 | 49,212 | 49,212 | 49,428 | 49,428 | 49,428 |
| Adj. R^2^ | 0.8986 | 0.9108 | 0.9255 | 0.9410 | 0.9505 | 0.9504 | 0.9511 | 0.9509 | 0.9572 | 0.9641 | 0.9756 | 0.9813 |
|  |  |  |  |  |  |  |  |  |  |  |  |  |
| Controls | × | × | × | × | × | × | × | × | × | × | × | × |
| Year×County FE | × | × | × | × | × | × | × | × | × | × | × | × |
| Month FE | × | × | × | × | × | × | × | × | × | × | × | × |

Note: The symbols ∗, ∗∗, and ∗∗∗ indicate significance levels at 10%, 5%, and 1%, respectively. "FE" stands for "fixed effects". Clustered standard errors at the wind farm level are shown in parentheses. Each panel depicts a single regression at the plant-year level, utilizing a buffer difference model to estimate the causal effect of wind farms on plant diversity indicators. The buffer model controls for the spatial dependence of observations, allowing for more accurate estimates of the treatment effect.
